# Comparison of segmentectomy guided by thin-slice CT or 3D CT simulation: A retrospective study

**DOI:** 10.1097/MD.0000000000043693

**Published:** 2025-08-01

**Authors:** Lei Shan, Leiming Zhou, Weiquan Zhang, Peichao Li, Bo Cong, Xiaogang Zhao, Yunpeng Zhao

**Affiliations:** a Department of Thoracic Surgery, The Second Hospital of Shandong University, Jinan, Shandong Province, China; b Key Laboratory of Thoracic Cancer, Shandong University, Jinan, Shandong Province, China; c Department of Thoracic Surgery, People's Hospital of Laoling, Laoling of Dezhou, Shandong Province, China.

**Keywords:** lung cancer, segmentectomy, thin- slice CT, three-dimensional simulation (3D simulation), video-assisted thoracoscopic surgery (VATS)

## Abstract

We first introduce how to read and understand thin-slice computed tomography (CT) images to guide segmentectomy and compare it with guidance by three-dimensional (3D) CT simulation. We retrospectively reviewed 111 patients who underwent video-assisted thoracic surgery segmentectomy from September 2019 to September 2021. They were divided into the CT group (55 cases) and 3D simulation group (56 cases). The scientific, comprehensive reading method of thin-slice CT images was introduced in detail and clinical parameters were compared between the 2 groups. CT group had shorter preoperative hospitalized preparation time (median: 3.0 vs 3.0 days, interquartile range: 2.0–3.0 vs 3.0–4.0, *P* = .002). CT group had shorter preoperative decision time (median: 1.0 vs 2.0 days, interquartile range: 1.0–2.0 vs 2.0–3.0, *P* < .001). There was no significant difference in the number of times the CT images or 3D simulation needed to be referenced during surgery (*P* = .233) between the 2 groups. The scientific reading and understanding of thin-slice CT images may provide good guidance in most cases of video-assisted thoracic surgery segmentectomy, constituting high-efficiency thoracic surgery to treat lung glass ground nodule with enhanced recovery after surgery.

## 1. Introduction

The detection of lung nodules has increased in recent years due to the widespread use of computed tomography (CT).^[1]^ In particular, subsolid nodules, including pure ground-glass nodules and part-solid ground-glass nodules have been focused.^[2]^ These lesions are also referred to as ground-glass opacities (GGOs) in many documents. Very small non-small-cell lung cancer lesions containing GGO components are usually associated with a good survival rate and are rarely associated with vascular invasion or lymph node metastasis; therefore, interest in defining the role of sublobar resection, such as segmentectomy, in the treatment of these GGO lesions has been raised. Anatomical partial lobectomy (APL) is a concept in oncology based on the anatomy of pulmonary segments and subsegments.^[3]^ APL is defined as anatomical sublobular resection, including single segmentectomy, extended segmentectomy, and combined subsegmentectomy.

Segmentectomy is usually guided by three-dimensional (3D) simulation technology, which has been reported to be effective for lobectomy or segmentectomy,^[4–6]^ regardless of whether small lung nodules are marked before surgery.^[7]^ The use of a 3D-printed reconstructed model for thoracoscopic APL in stage I lung cancer has also been reported.^[8]^ However, mastering the skills related to 3D simulation involves a long learning curve and requires time. Furthermore, data acquired from contrast-enhanced CT are considered necessary when reconstructing 3D pulmonary vascular images.^[9]^ We supposed that the careful and scientific reading of thin-slice CT images may provide precise information to guide segmentectomy after adequate training for learning and practice. In addition, nonenhanced CT data was sufficient. This approach may obviously shorten the preparation time and the hospital stay, increase efficiency and help surgeons achieve a deeper understanding of the pulmonary anatomy. Here, we will present in detail the method to read and understand thin-slice CT images to guide segmentectomy and then compare it with guidance by 3D simulation.

## 2. Patients and methods

### 2.1. Patients

We retrospectively analyzed the records of 111 patients who underwent thoracoscopic segmentectomy for small subsolid pulmonary nodules at the Second Hospital of Shandong University from September 2019 to September 2021. The patients were all clinically diagnosed with peripheral T1aN0M0 stage IA lung cancer. This study was approved by the Ethics Committee and Medical Administration Division of the Second Hospital of Shandong University. Written informed consent was obtained from each of the enrolled patients. All methods performed in our study were conducted in accordance with the relevant guidelines and regulations.

Our selection criteria for video-assisted thoracic surgery (VATS) segmentectomy in the present study were according to the National Comprehensive Cancer Network guideline as follows: (I) peripheral GGO suspicious for malignancy; (II) lesion < 2 cm in diameter and meeting at least one of the following criteria: (i) pure AIS histology; (ii) ≥50% ground-glass appearance on CT; and (iii) a long doubling time (≥400 days) confirmed by radiologic surveillance.

### 2.2. Reading of thin-slice CT images

Nonenhanced CT or contrast-enhanced CT images could be used, and the slice thickness could be 0.625 mm, 1.0 mm or 1.25 mm. The nomenclature adopted is an accordance with that in a previous publication.^[10]^

The reading, decision-making and planning methods are summarized as follows. The correct reading principles of thin-layer CT can be found in File S1, Supplemental Digital Content, https://links.lww.com/MD/P587.

**Figure 1.**
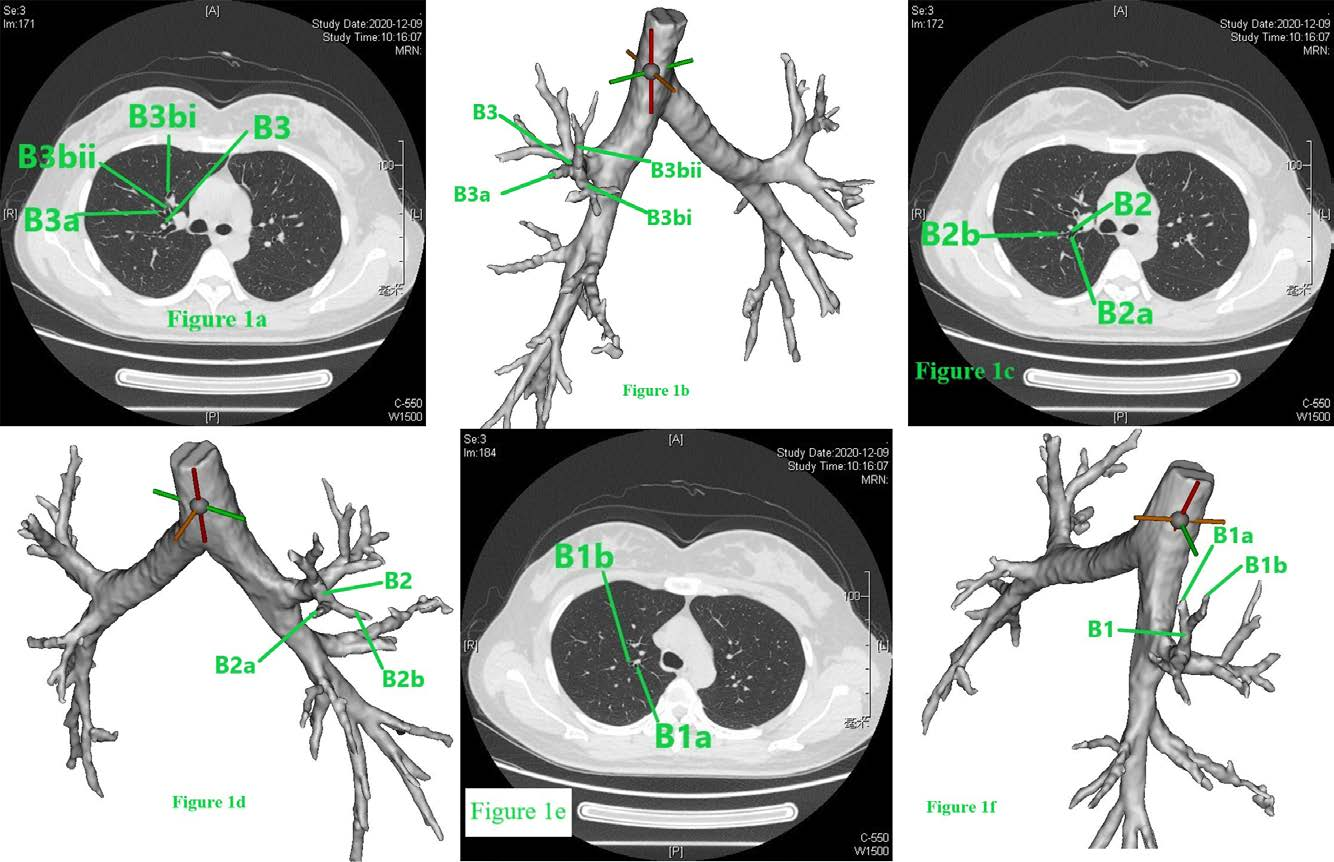
(A–F) The definition of the bronchus.

**Figure 2.**
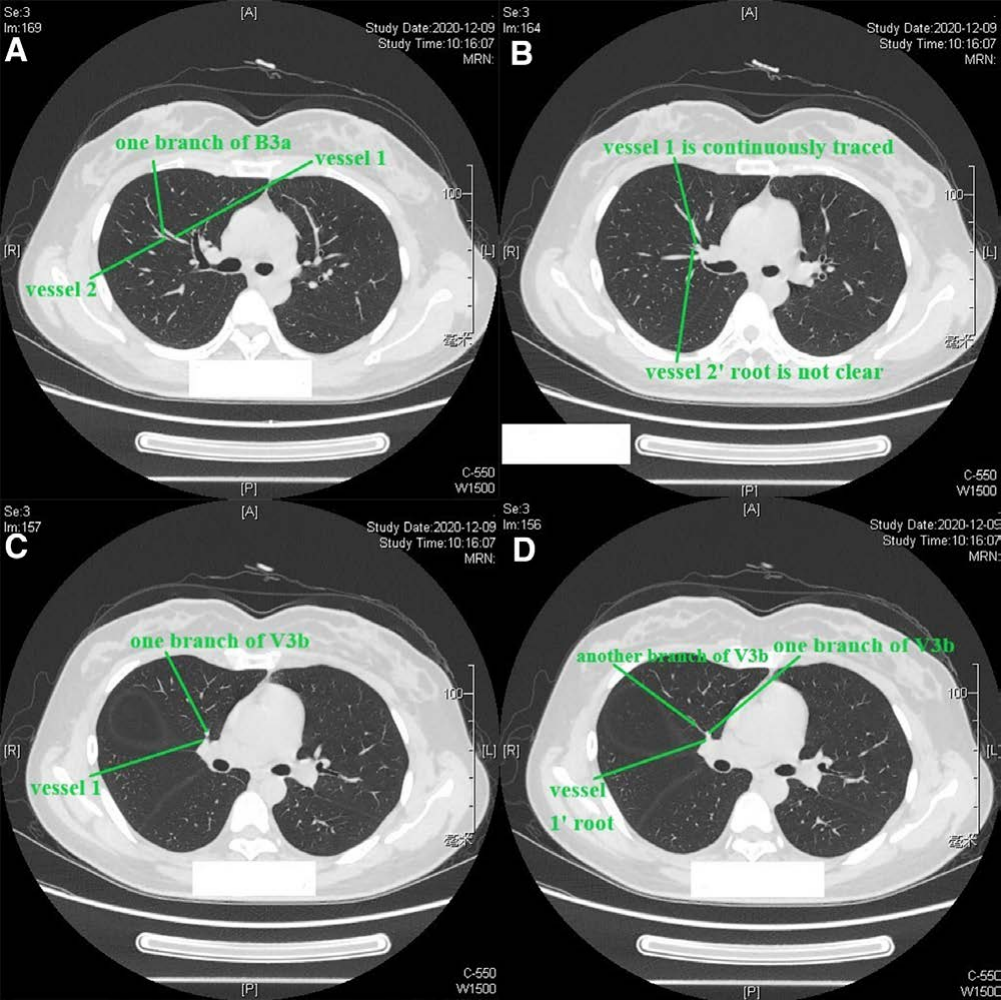
(A–D) One method to discriminate the pulmonary artery and vein.

**Figure 3.**
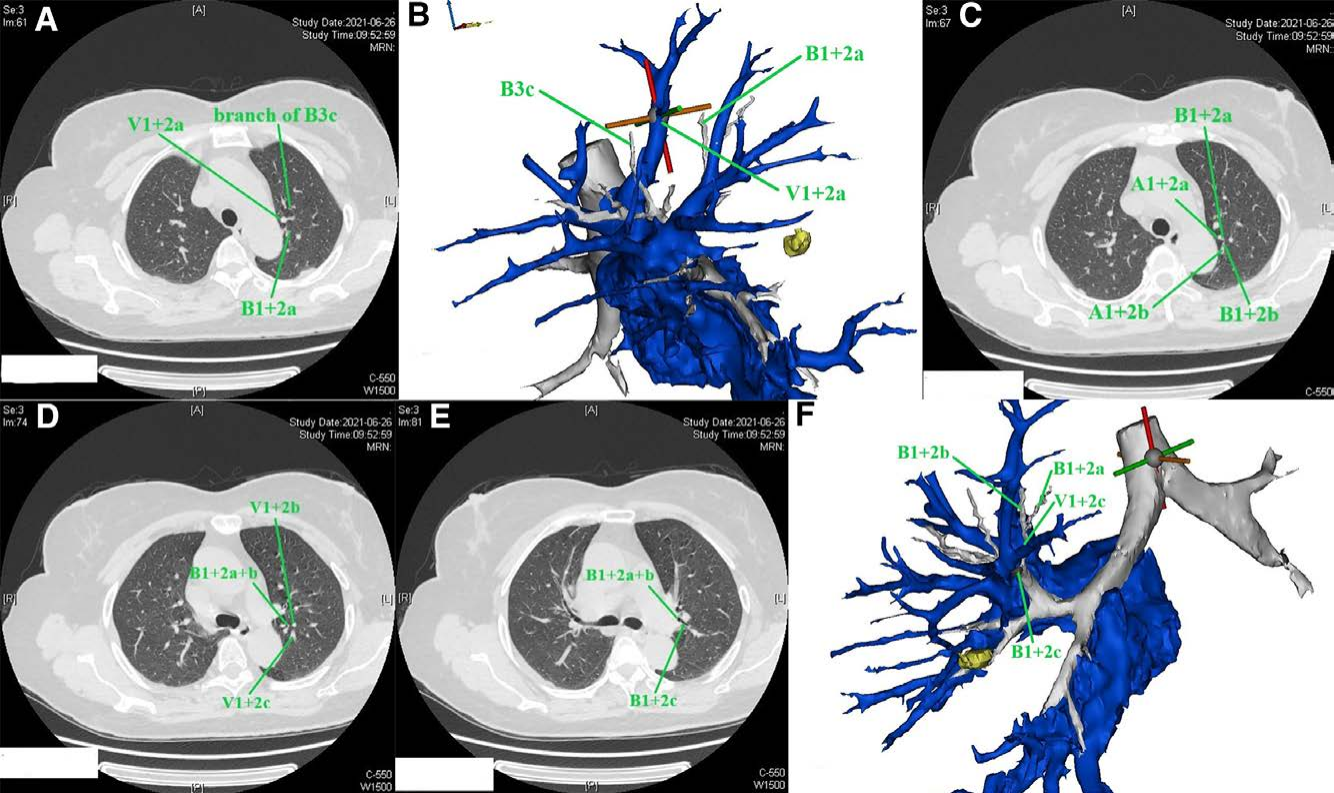
(A–F) The identification of pulmonary vein.

**Figure 4.**
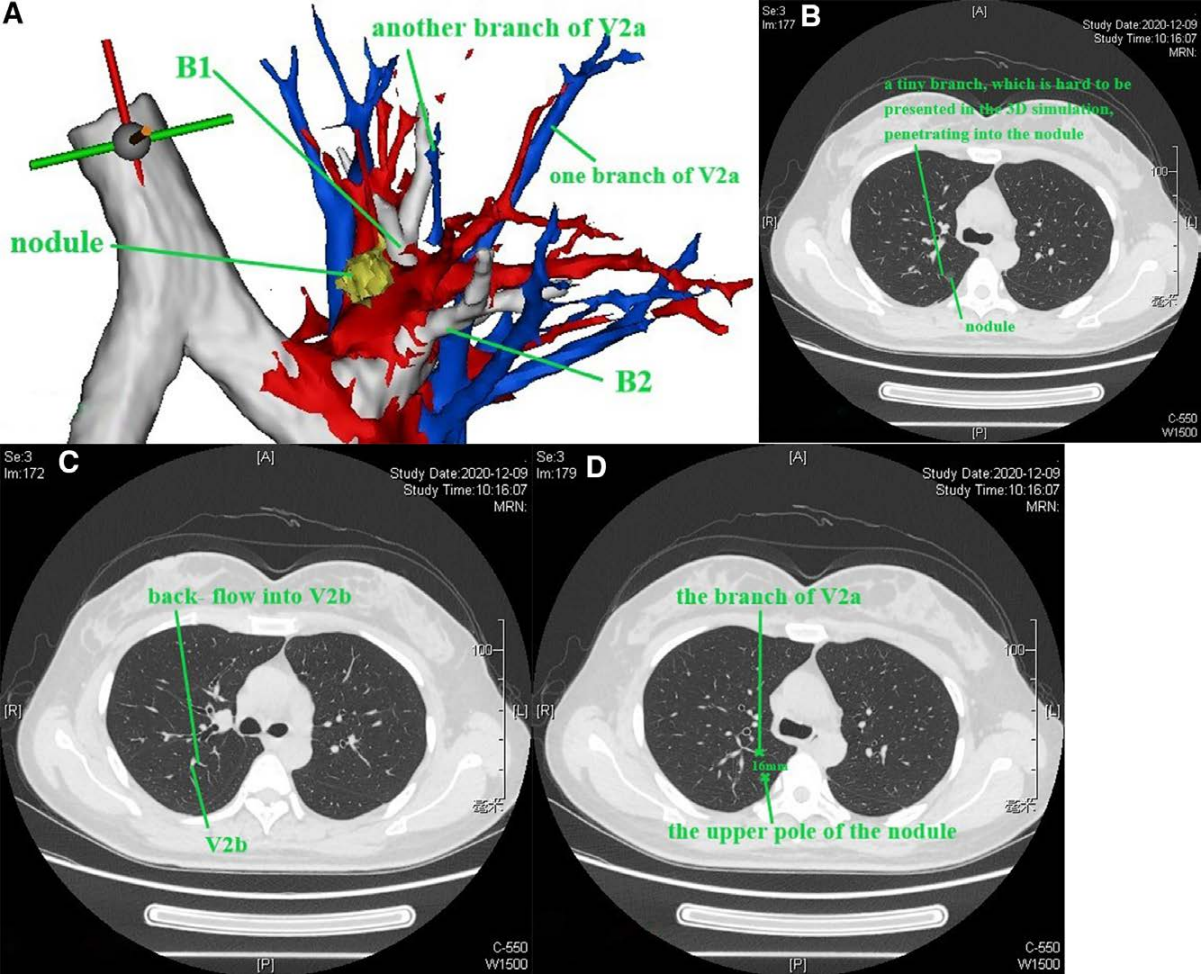
(A–D) One method to determine the location of the nodule and evaluate the resection range.

**Figure 5.**
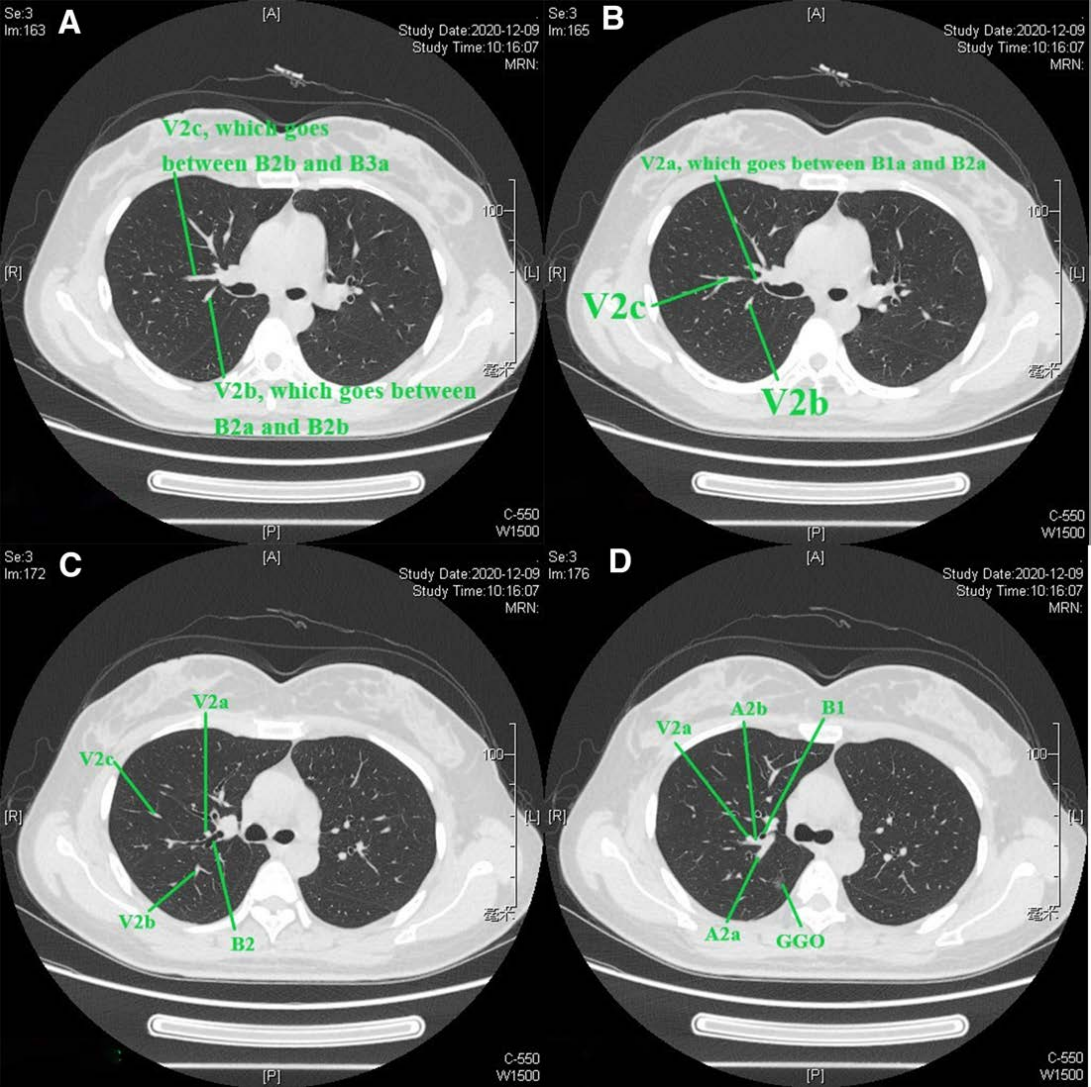
(A–D) To identify the adjacent relationship of pulmonary structures related to the target segment for surgical planning.

1. The bronchus, pulmonary artery, and pulmonary vein should be identified. For the right upper lobe, the reading direction of CT images was first from caudad to cephalad (Figure S1, Supplemental Digital content, https://links.lww.com/MD/P584). After identification of the bronchus of the right middle lobe, we scanned the thin-slice CT (1.25 mm) images cephalad. The bronchus ventralis was identified as B3, and it could be divided into B3a (Rm. lateralis) and B3b (Rm. medialis); B3b (Rm. medialis) could be divided into B3bi and B3bii (Fig. 1A), as presented in the 3D simulation shown in Figure 1B. Then, scanning cephalad was continued uninterrupted. The bronchus dorsalis was identified as B2, and it could be divided into B2a (Rm. dorsalis) and B2b (Rm. horizontalis) (Fig. 1C), as presented in the 3D simulation in Figure 1D. The bronchus apicalis was identified as B1, and it could be divided into B1a (Rm. apicalis proprius) and B1b (Rm. ventralis) (Fig. 1E), as presented in the 3D simulation in Figure 1F. In our opinion, the definition of the bronchus should follow the “common trunk” principle for the purposes of unification and simplification; for example, B3bii was not defined as B1b in this case. We followed this principle in all cases. It should be noted that the nomenclature does not concern the resection range, which is based on the location of the nodule and on achieving a sufficient surgical margin. Then, the segmental artery, subsegmental artery and vein are identified. Discrimination of the pulmonary artery and pulmonary vein is a key point that can be difficult during the reading of CT images or reconstruction procedure of 3D simulations. The discrimination method applied was as follows (nonenhanced CT): (1) the most important characteristic of the pulmonary artery is that it follows the bronchus tightly, especially in the relatively peripheral part of the lung, while the vein follows a different course, going between or among the bronchi, especially in the relatively peripheral part of the lung; (2) sometimes, 2 vessels can be seen beside the bronchus, and continuous thin-slice CT images should be read carefully to find the root of the vessel and recognize the origin (Figure 2A–D; vessel 2 is the pulmonary vein, and vessel 1 is the pulmonary artery. Consecutive illustrations can be found in Figures S2, Supplemental Digital Content, https://links.lww.com/MD/P585). Alternatively, peripheral part of the vessels on CT images should be read, the vein will gradually leave the bronchus, while the artery will continue to follow the bronchus. In this book,^[10]^ veins are defined between segments; however, we cannot identify segments through direct observation during clinical practice, and the bronchus is always used instead of segments to define veins. For example, if a vein runs between B1a and B1b (on CT or 3D simulation), it is recognized as V1a. The branches and the vein that runs between them can be observed on the same CT image (V1 + 2a is between B1 + 2a and B3c, Fig. 3A and B). The branches and the vein that runs between them can be also observed on the longitudinal axis of the human body (V1 + 2c is between B1 + 2b and B1 + 2c), when the continuous reading direction is caudad, is that the main body of B1 + 2b can be observed (Fig. 3C), followed by the main body of V1 + 2c (Fig. 3D) and then the main body of B1 + 2c (Fig. 3E). The 3D anatomic relationship can also be observed on 3D simulation (Fig. 3F), and consecutive illustrations of this case can be found in Figures S3, Supplemental Digital Content, https://links.lww.com/MD/P586.
2. Then the location of the nodule and the resection range should be evaluated. Reading thin-slice CT images has advantages. Taking this case as an example, interestingly, there was confusion about the location of the nodule on 3D simulation (Fig. 4A), which could be resolved well by reading thin-slice CT images. There are 2 methods to determine the location: (1) if there are branches of intrasegmental pulmonary structures penetrating the nodule, the nodule is located in the corresponding segment; and (2) the location can be determined according to the intersegmental vein. Here, a tiny branch was observed penetrating the nodule (Fig. 4B); this branch was identified as a distal branch of V2b (intrasegmental vein of S2) (Fig. 4C), the nodule was confirmed to be located in S2. Regarding the resection range, the distance between the upper pole of the nodule and the V2a branch (intersegmental vein between S1 and S2) was 16 mm (Fig. 4D). If the nodule and the intersegmental vein are not present in the same section, the most vertical distance between the nodule and the peripheral branch of the intersegmental vein should be measured by counting the CT layers and multiplying by the slice thickness to evaluate resection margin.
3. Adjacent relationship of structures related to the target segment should be identified for surgical planning (Figures S2, Supplemental Digital Content, https://links.lww.com/MD/P585). The position of surrounding structures can be easily determined on the same CT image, while that of structures superior and inferior to the lesion can be recognized by reading the CT images continuously. For example, in this case, reading the CT images cephalad, first, V2b and V2c are observed (Fig. 5A), it can be determined that V2b runs posterior and toward the head (when the reading direction is cephalad, 1 vessel can be observed running peripherally, it is toward the head) and that V2c is located anterior to V2b, running toward the lateral chest wall and cephalad (Fig. 5B). The next important structure is B2, which also runs posterior and cephalad, and V2a is located anterior to B2 (Fig. 5C). As the reading procedure continues, A2a and A2b can be seen (no ascending A2 in this case), with A2a running posterior and cephalad and A2b running toward the lateral chest wall and caudad (when the reading direction is cephalad, 1 vessel can be observed running toward the pulmonary hilum, it is toward caudad). V2a and B1 are located anterior to A2b (Fig. 5D).

### 2.3. 3D CT simulation

At our institution, digital imaging and communications in medicine data of thin-slice (0.625 mm or 1.25 mm) CT images were imported into Mimics 21.0 (developed by Materialise Nv Co., Materialise’s interactive medical image control system, Kingdom of Belgium) or the InterOperation Thorax Planning system (Beijing Infervision Technology Co., Ltd.) for the 3D reconstruction of pulmonary structures. Contrast-enhanced CT was used for 3D simulation in the 3D simulation group. A sphere extending 2 cm outside the primary tumor can be used to evaluate the safe resection margin.

Nonenhanced CT can be used to finish the 3D reconstruction in only 15–30 minutes by another doctor in case the surgeon of CT group requires structural verification during surgery, meanwhile, a doctor (not the surgeon) can review the 3D simulation to check whether the surgical decision and plan are consistent with those made according to thin-slice CT.

### 2.4. Surgical technique

VATS segmentectomy was performed in all enrolled cases. CT-guided localization for lung nodules was performed before surgery for all cases. The target segmental artery and bronchus were isolated and divided according to preoperative plan and intraoperative anatomy. The hilar lymph nodes were then routinely obtained for cryosectioning and examination. The venous branches running into the target segment were divided according to preoperative plan or after the distal stump of the target segmental bronchus was lifted and denuded in the peripheral direction. The inflation–deflation line was indicated after the lung was reinflated with pure oxygen. Electrocautery was used to cut toward the inflation–deflation line, the peripheral parenchyma was divided with a stapler, and intersegmental veins were preserved. The location of the nodule was also taken in full consideration to guarantee enough margin. If the lesion was proven to be malignant, N1 and N2 lymph nodes were sampled or dissected.

### 2.5. Statistical analysis

Statistical analysis and graph drawing were performed with Stata 12.0 (StataCorp LP, College Station, TX). Variables were analyzed as the mean and standard deviation. ANOVA analysis was used for comparison when groups are more than 2. Mean values were compared using Student *t* test, and frequency distributions were compared using the chi-squared test or Fisher exact test. *P* values of < .05 were regarded as significant.

## 3. Results

### 3.1. Patient characteristics

Patient characteristics are shown in Table 1. There were 111 patients enrolled. Patients in the CT and 3D simulation groups had a median age of 56 and 57 years, respectively, with 31 (56.4%) and 33 (58.9%) females in the CT and 3D simulation groups, respectively. Most patients in both groups never smoked (81.8% in the CT group and 73.2% in the 3D simulation group). The surgical indication was most commonly part-solid nodules (80.0% in the CT group and 66.1% in the 3D simulation group), with a median maximum lesion diameter of 12.0 mm in the CT group and 9.5 mm in the 3D simulation group. There were no significant differences between the 2 groups in comorbidities or cancer history. No cases were converted to thoracotomy, and no intraoperative conversions to lobectomy.

**Table 1:** Preoperative clinicopathological factors of the patients.

Characteristics	CT group (n = 55)	3D-simulation group (n = 56)	*P* value
Age, years, mean ± SD	55.9 ± 11.5	55.7 ± 10.5	.922
Gender, n (%)			.851
Male	24 (43.6%)	23 (41.1%)	
Female	31 (56.4%)	33 (58.9%)	
Smoking status, n (%)			.360
Ever	10 (18.2%)	15 (26.8%)	
Never	45 (81.8%)	41 (73.2%)	
Maximum lesion diameter (mm), mean ± SD	12.7 ± 3.6	10.8 ± 4.0	.012
Leision composition on CT			.144
Part-solid nodules	44 (80.0%)	37 (66.1%)	
Pure ground-glass nodules	11 (20.0%)	19 (33.9%)	
Comorbidity			.681
Hypertension	19	12	
Diabetes mellitus	7	8	
Heart disease	2	4	
Brain vascular disease	3	4	
COPD	1	1	
Cancer history	2	2	.680
Preoperative hospitalized time, days, mean ± SD	2.8 ± 1.1	3.6 ± 1.5	.002
Preoperative decision time, days, mean ± SD	1.3 ± 0.5	2.3 ± 1.5	<.001

COPD = chronic obstructive pulmonary disease, SD = standard deviation.

### 3.2. Preparation time before surgery

The time between admission and operation was considered the preoperative hospitalized time. Table 1 also shows that in the CT group, a median of 3.0 days (interquartile range: 2.0–3.0 days) was required to accomplish preoperative hospitalized preparation, compared to 3.0 days (interquartile range: 2.0–3.0 days) in the 3D simulation group, and this difference was significant (*P* = .002). The time between admission and completing a surgical plan was considered the preoperative decision time. This time was significantly shorter in the CT group than the 3D simulation group (median: 1.0 vs 2.0 days, interquartile range: 1.0–2.0 vs 2.0–3.0, <.001).

### 3.3. Surgical range and cases

As shown in Table 2, the segmentectomy procedures were divided into noncomplex and complex segmentectomy procedures. Noncomplex segmentectomy was defined according to previous research,^[11–13]^ including LS1 + 2 + 3 (trisegmentectomy), LS4 + 5 (lingulectomy), S6, and S7 + 8 + 9 + 10 (basal segmentectomy). No cases had positive margin confirmed in the pathology reports.

**Table 2:** Types of thoracoscopic segmentectomy procedure in 2 groups.

	CT group (n = 55)	3D-simulation group (n = 56)	*P* value
Type of procedure			.413
Noncomplex segmentectomy	12	16	
S6	2	13	
LS1 + 2 + 3	7	1	
LS4 + 5	2	2	
LS8 + 9 + 10	1	0	
Complex segmentectomy	43	40	
RS1	5	7	
RS2	5	11	
RS3	2	3	
RS2b + 3a	1	2	
S10	4	0	
S9 + 10	3	0	
RS2 + 6	1	1	
S8 + 9	2	0	
S1 + 2	5	5	
S8	2	3	
RS1 + 3	3	3	
LS3	3	3	
RS4	1	0	
LS4 + 5 + 1 + 2c	1	0	
LS4 + 5 + 3a + 1 + 2c	1	0	
S10 + 6c	1	0	
S6 + 10c	1	0	
S9 + 10 + 6b + c	1	0	
RS2 + Sla	1	1	
RS1 + S2a	0	1	

Noncomplex segmentectomy includes: LS1 + 2 + 3 (trisegmentectomy), LS4 + 5 (lingulectomy), S6, and S7 + 8 + 9 + 10 (basal segmentectomies).

### 3.4. Intraoperative and postoperative patient characteristics

As Table 3 shows, the number of times the CT images or 3D simulation needed to be referenced during surgery did not differ significantly between the groups (*P* = .233). There was no significant difference in intraoperative blood loss, postoperative complications, or postoperative histology between the groups.

**Table 3:** Intraoperative and postoperative characters.

Characteristics	CT group (n = 55)	3D-simulation group (n = 56)	*P* value
Re-identification of CT or 3D-simulation during surgery			.233
No-need	33	37	
1 time	11	15	
Twice	7	2	
3 times	4	2	
Operation duration (min, mean ± SD)	97 ± 36.7	101.5 ± 47.4	.578
Intraoperative blood loss (mL)	51.8 ± 16.8	57.8 ± 30.3	.200
Postoperative complications n (%)	0.598		
Pneumonia	9 (16.4%)	5 (9.0%)	
Air leakage (≥5 days)	1 (1.8%)	2 (3.6%)	
Atelectasis	1 (1.8%)	1 (1.8%)	
Postoperative histology	0.106		
Invasive adenocarcinoma	28	20	
Noninvasive adenocarcinoma	27	36	

SD = standard deviation.

## 4. Discussion

Thoracoscopic segmentectomy was shown to be feasible several years ago, achieving oncological results equivalent to those of open segmentectomy^[14,15]^; after that, 3D CT simulation was confirmed to be helpful for anatomic segmentectomy or APL by VATS.^[4–7]^ The use of a 3D-printed model has also been confirmed to be helpful in thoracoscopic segmentectomy or APL^[8,16,17]^; however, not all patients can afford to have a 3D-printed model made. Some doctors even chose professional third parties to provide 3D CT simulations to save time and energy. Performing segmentectomy only according to the readily available 3D simulation had disadvantages, including difficulty achieving an essential understanding of the pulmonary anatomy, a lack of confidence when the actual operative anatomy did not match the 3D simulation well, and a lack of full consideration of lobe turnover or shielding of lung tissues or structures. Even so, 3D simulation was vital for inexperienced surgeons. In actuality, 3D simulations might have errors or omissions, and the original CT images are the most accurate data second only to the real anatomy of the patient.

Our study was retrospective, and the preoperative clinicopathological factors of the patients were not completely balanced. The nodule size was significantly greater in the CT group than in the 3D simulation group. This difference was due to differences in the surgical indication; however, most GGOs surgically removed in both groups were part-solid nodules and were larger than 1 cm, as determined on CT surveillance in our department. The preoperative hospitalization time, that is, the number of days from admission to surgery, and the preoperative decision time, that is, the number of days from admission to completion of the preliminary surgical plan, were significantly shorter in the CT group. Several reasons could have contributed to these differences: (1) the nonenhanced CT scan could be completed at any time; (2) if a patient with diabetes was still taking metformin, the enhanced CT scan might be delayed 48 hours; and (3) the procedure for obtaining data in digital imaging and communications in medicine format and performing 3D CT simulation still requires some time, especially in large medical centers with a high surgical volume.

Table 2 shows the types and positions of resected segments in the 2 groups. There was no significant difference between the 2 groups (*P* = .413). It indicates that complex segmentectomy can also be performed under guidance by thin-slice CT after the method had been mastered. As Table 3 shows, uniport segmentectomy could also be accomplished under the guidance of thin-slice CT, and the significant difference in the number of ports used during surgery was due to the habits of the surgeons in the 2 groups. All patients in both groups were managed according to the enhanced recovery after surgery (ERAS) protocol, and the significant difference in the duration of chest tube indwelling and postoperative hospital stay was due to the details of patient management in the 2 groups, which we will examine further in another study.

We believe that the use of thin-slice CT for guidance does not conflict with advancements in science and technology; instead, they promote each other. Initially, during the first period, surgeons performed segmentectomy relying only on the intraoperative anatomy, mistakes often occurred. Then, during the second period, the technique of 3D CT simulation was developed and confirmed to be helpful in creating a precise surgical plan for VATS segmentectomy for GGOs in the lung, as was the use of 3D-printed models. We consider there to be a third period: after adequate training on 3D CT simulation, VATS segmentectomy, and reading thin-slice CT images, most VATS segmentectomy procedures could be guided by the scientific reading of thin-slice CT images, and 3D CT simulation and 3D-printed models can be used in more complex segmentectomy cases or for communication with other doctors. Furthermore, thin-slice CT guidance and the ERAS protocol together constitute high-efficiency thoracic surgery for GGOs, and even for lobectomy, identifying anatomical variations before surgery without 3D CT simulation may reduce the risk and improve the efficiency of surgery. High-efficiency thoracic surgery is beneficial for shortening the hospital stay and saving medical resources.

This is the first introduction of this method for scientifically reading thin-slice CT images worldwide. We have several suggestions for how to learn to scientifically read and understand thin-slice CT images: (1) master lung segment anatomy; (2) shadow a surgeon who has mastered reading and understanding thin-slice CT images; (3) practice cross-referencing identifying structures between thin-slice CT images and 3D simulations before surgery, and then identify every vessel and bronchus carefully during surgery when the intraoperative anatomy is clear. The scientific reading and understanding of thin-slice CT images can make 3D simulations procedure easier and improving the overall accuracy (discrimination of the pulmonary artery and vein is easy, and identification of the intersegmental vein facilitates division of the intersegmental plane). Conversely, 3D simulations can help confirm segmental structures in thin-slice CT images. After a sufficient learning period, 1 can precisely understand the intraoperative anatomy based on thin-slice CT images.

There are some limitations to the present study. This was a retrospective study with few cases, and some clinical and surgical factors were not balanced between the 2 groups. Additionally, it is difficult to master the scientific reading and understanding of thin-slice CT images, which involves a learning curve.

## 5. Conclusion

The scientific reading and understanding of thin-slice CT images may provide good guidance for most VATS segmentectomy procedures and facilitate the reconstruction procedure of preoperative 3D simulations. This approach can shorten the preoperative preparation time and total hospital stay, constituting high-efficiency thoracic surgery to treat lung glass ground nodule along with the ERAS protocol.

## Acknowledgments

We thank Professor Lijie Tan, Qun Wang, Yaxing Shen, Yong Fang, Jun Yin, Yi Zhang, Renfeng Wang, from Zhongshan Hospital of Fudan University, for guidance of the technique. We also thank Yanfei Cao, from Shanxi Provincial People’s Hospital, Wei Zhang, from Hefei First People’s Hospital, for deep discussion of the technique.

## Author contributions

**Conceptualization:** Bo Cong, Xiaogang Zhao, Yunpeng Zhao.

**Data curation:** Leiming Zhou, Weiquan Zhang, Peichao Li.

**Investigation:** Leiming Zhou, Weiquan Zhang, Peichao Li.

**Methodology:** Lei Shan.

**Writing – original draft:** Lei Shan.

**Writing – review & editing:** Lei Shan, Xiaogang Zhao.

## Supplementary Material


