# Supplementary material for: Comparison of segmentectomy guided by thin-slice CT or 3D CT simulation: A retrospective study
Source: Medicine (Baltimore). 2025 Aug 1;104(31):e43693. doi: 10.1097/MD.0000000000043693 (PMC12323932; doi:10.1097/MD.0000000000043693)
Supplement: Supplementary file 1 [file medi-104-e43693-s001.doc]

# Notes of scientific reading

1. The reading of thin- slice CT must be consecutive and repeated, any structure couldn’t be identified correctly only according to one slice of CT.
2. The nomenclature may be controversial in some cases, no matter the reading of thin- slice CT or the identification of 3D simulation. In our opinion, the definition of the bronchus should follow the "common trunk" principle for the purposes of unification and simplification, the nomenclature of pulmonary artery and vein should be assured after the bronchus has been defined. Bronchus is the core of segment.
3. It should be noted that the nomenclature does not concern the resection range, which is based on the location of the nodule and on achieving a sufficient surgical margin. The name of pulmonary structures is for communication, if the communication is unhindered and clear, spend less time to argue on the nomenclature. If it is controversial, define the bronchus first, not the pulmonary artery of vein.
