# Supplementary material for: Comparison of segmentectomy guided by thin-slice CT or 3D CT simulation: A retrospective study
Source: Medicine (Baltimore). 2025 Aug 1;104(31):e43693. doi: 10.1097/MD.0000000000043693 (PMC12323932; doi:10.1097/MD.0000000000043693)
Supplement: Supplementary file 2 [file medi-104-e43693-s002.ppt]

## Slide 1
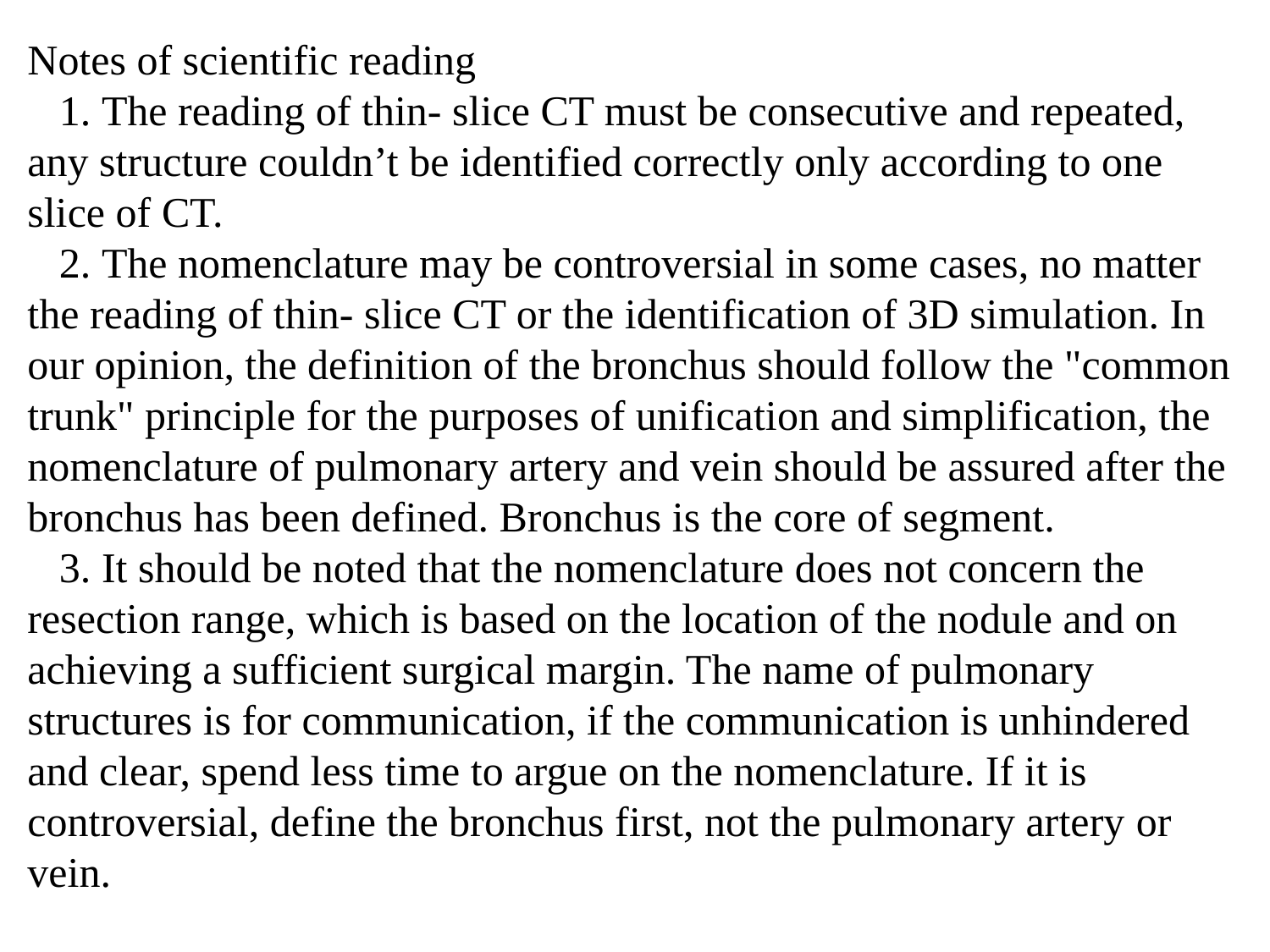

Notes of scientific reading
 1. The reading of thin- slice CT must be consecutive and repeated, any structure couldn’t be identified correctly only according to one slice of CT.
 2. The nomenclature may be controversial in some cases, no matter the reading of thin- slice CT or the identification of 3D simulation. In our opinion, the definition of the bronchus should follow the "common trunk" principle for the purposes of unification and simplification, the nomenclature of pulmonary artery and vein should be assured after the bronchus has been defined. Bronchus is the core of segment.
 3. It should be noted that the nomenclature does not concern the resection range, which is based on the location of the nodule and on achieving a sufficient surgical margin. The name of pulmonary structures is for communication, if the communication is unhindered and clear, spend less time to argue on the nomenclature. If it is controversial, define the bronchus first, not the pulmonary artery or vein.
#

## Slide 2
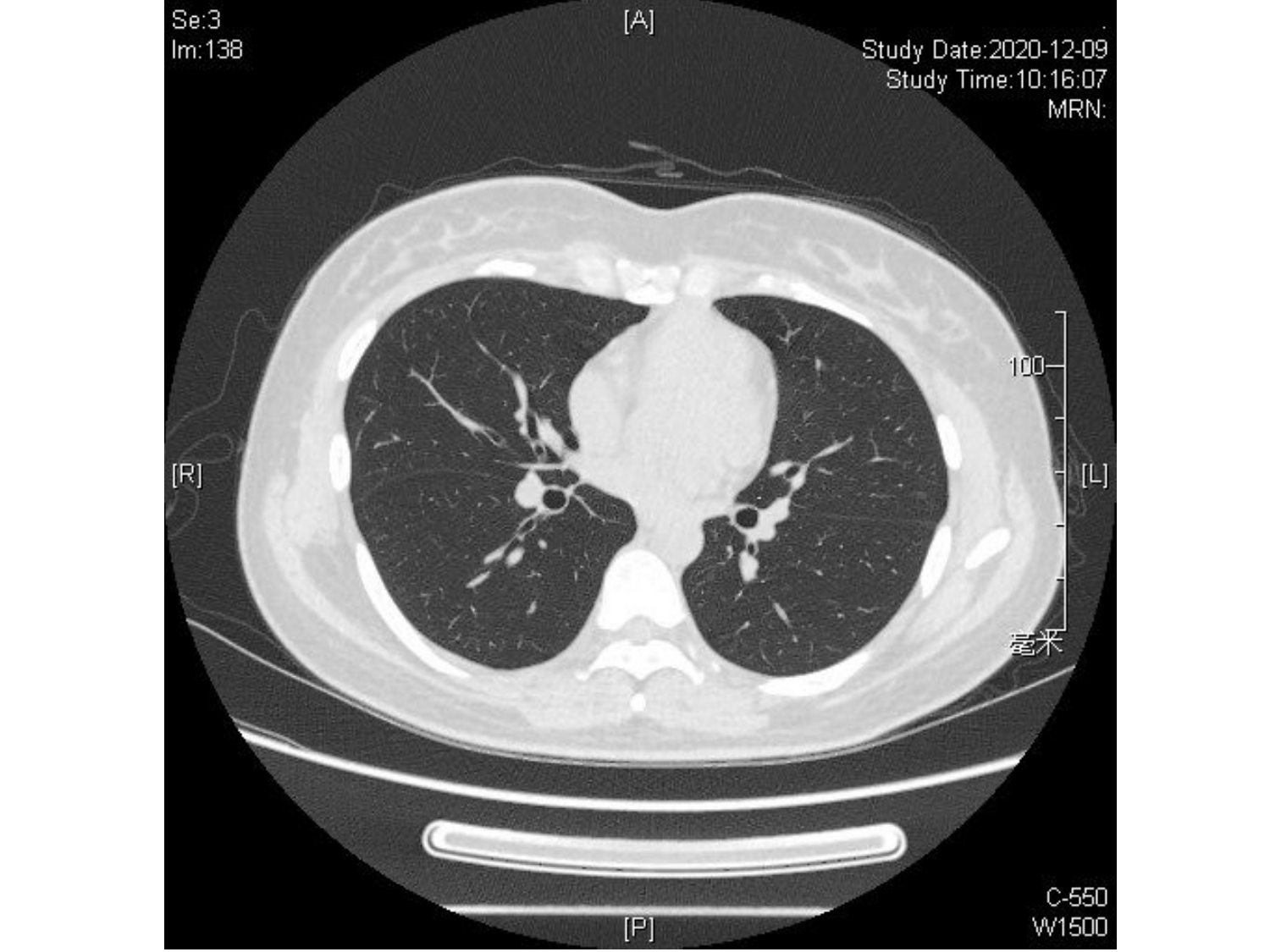

#

## Slide 3
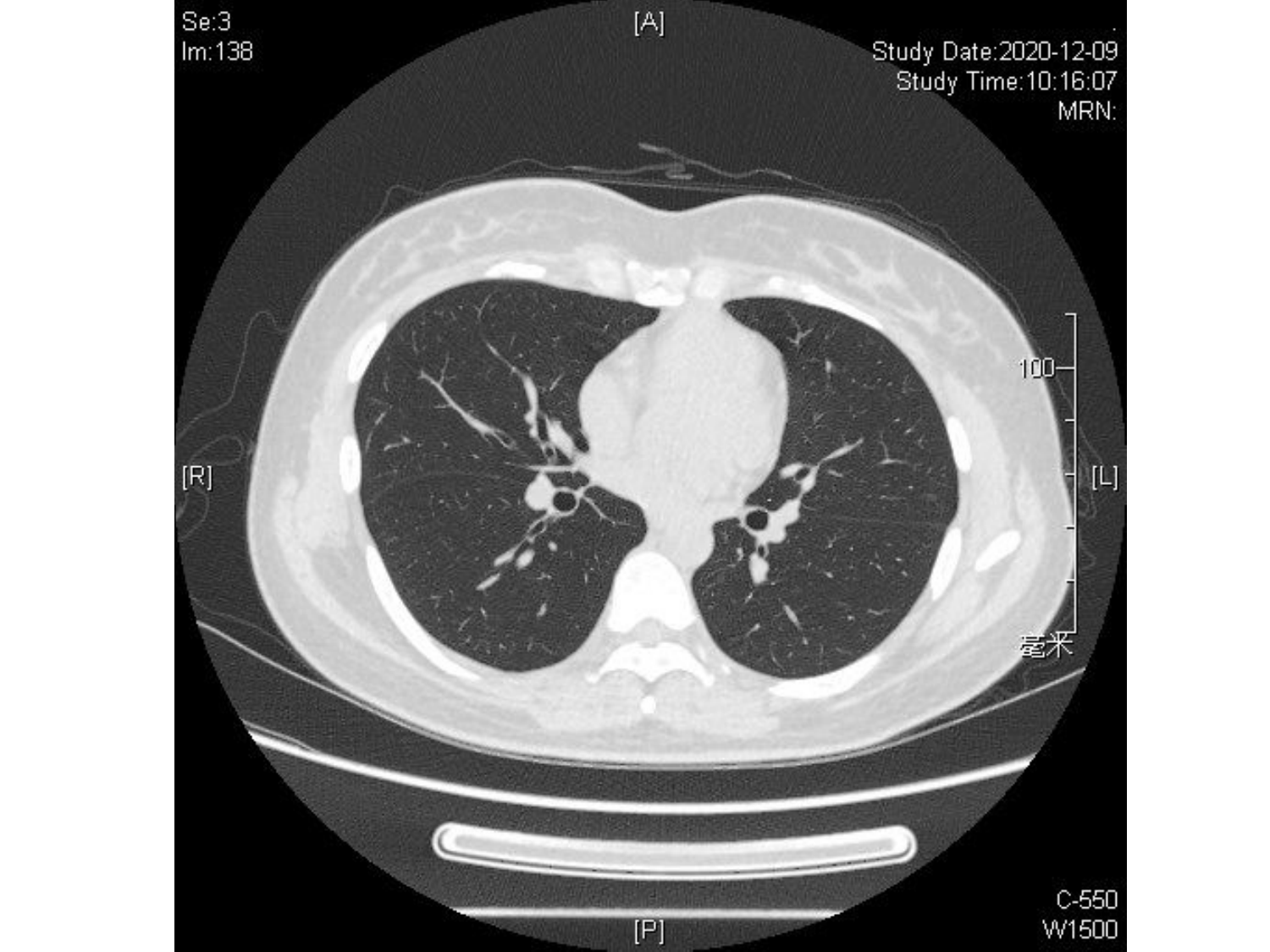

#

## Slide 4
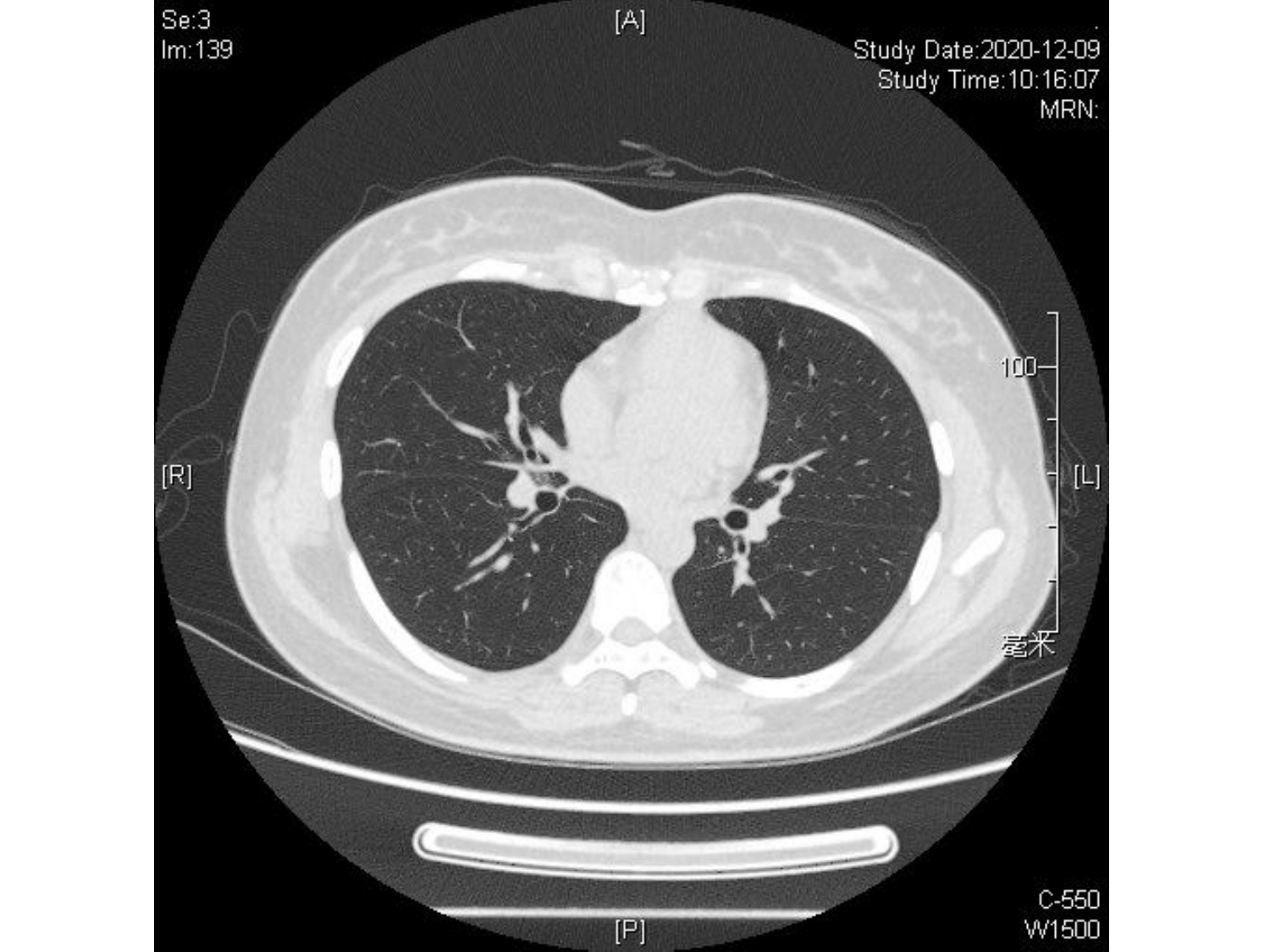

#

## Slide 5
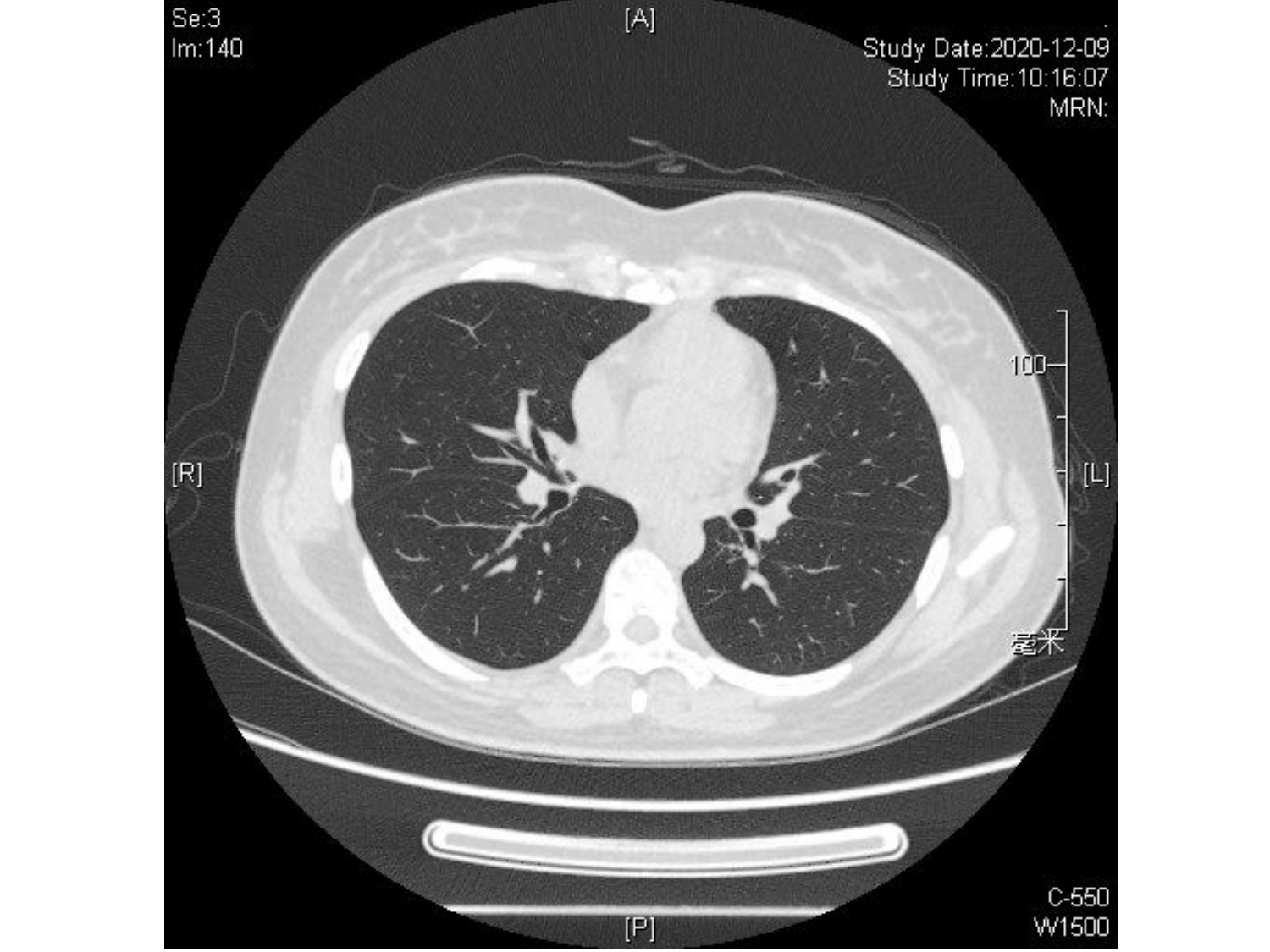

#

## Slide 6
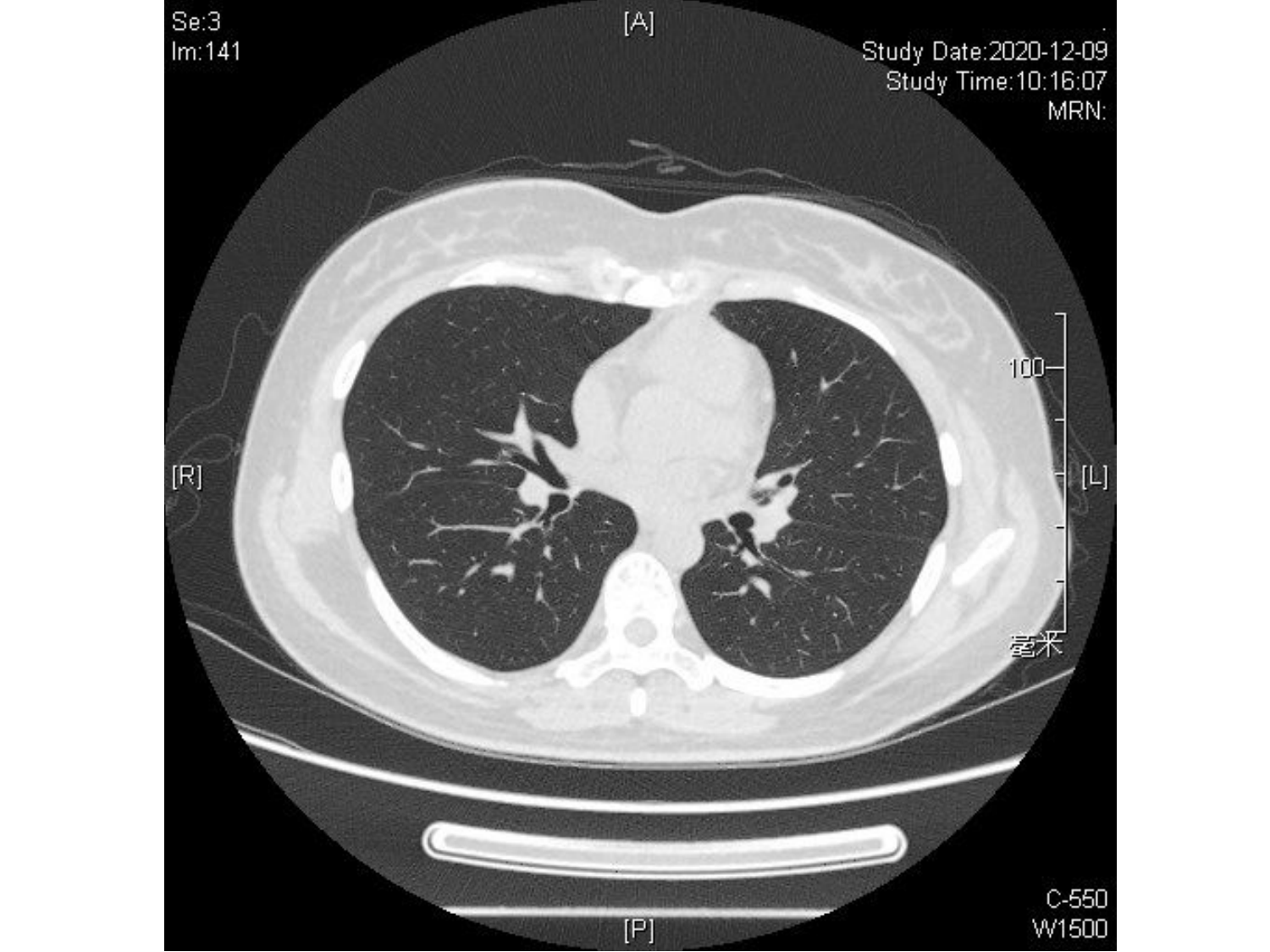

#

## Slide 7
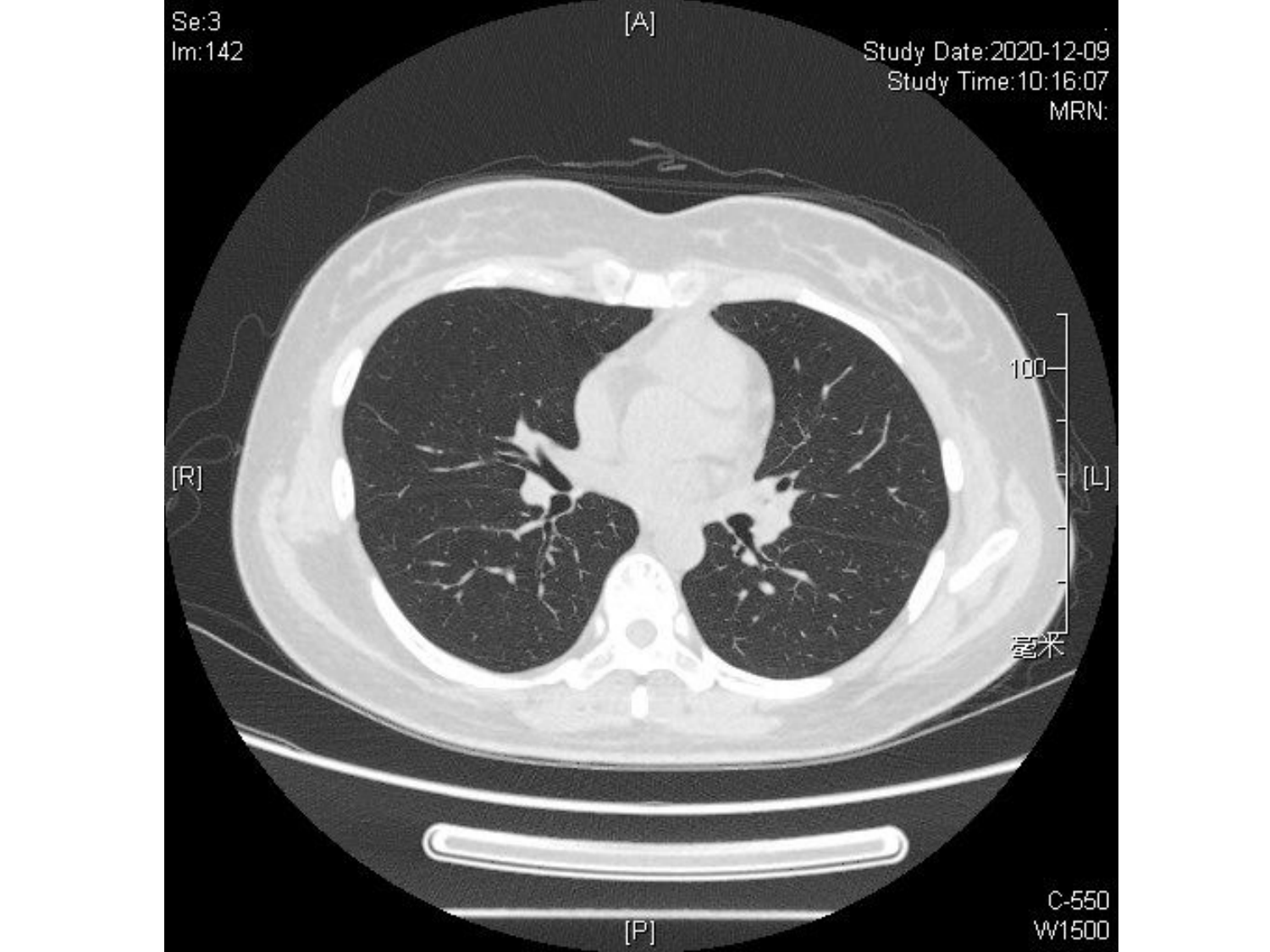

#

## Slide 8
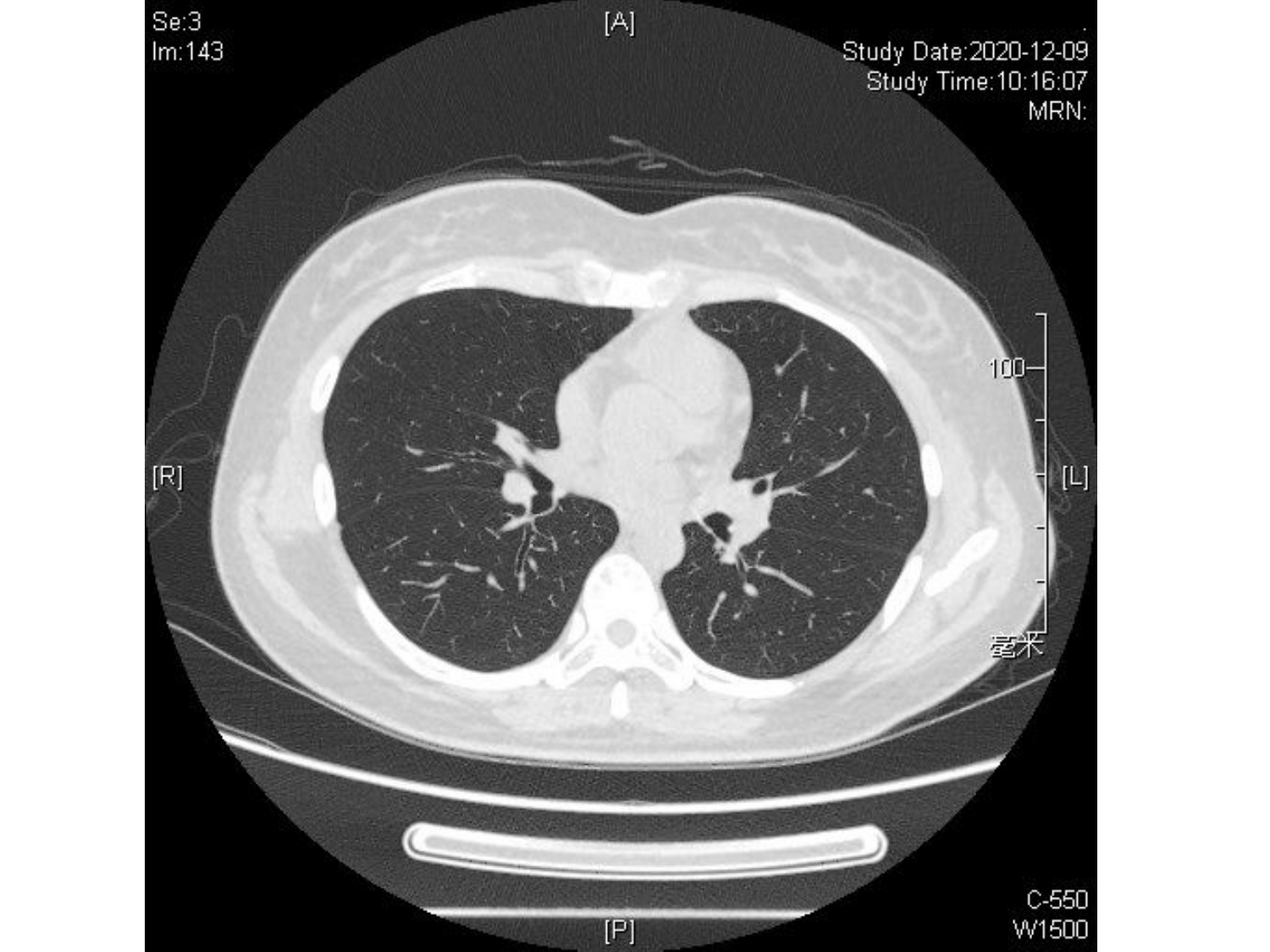

#

## Slide 9
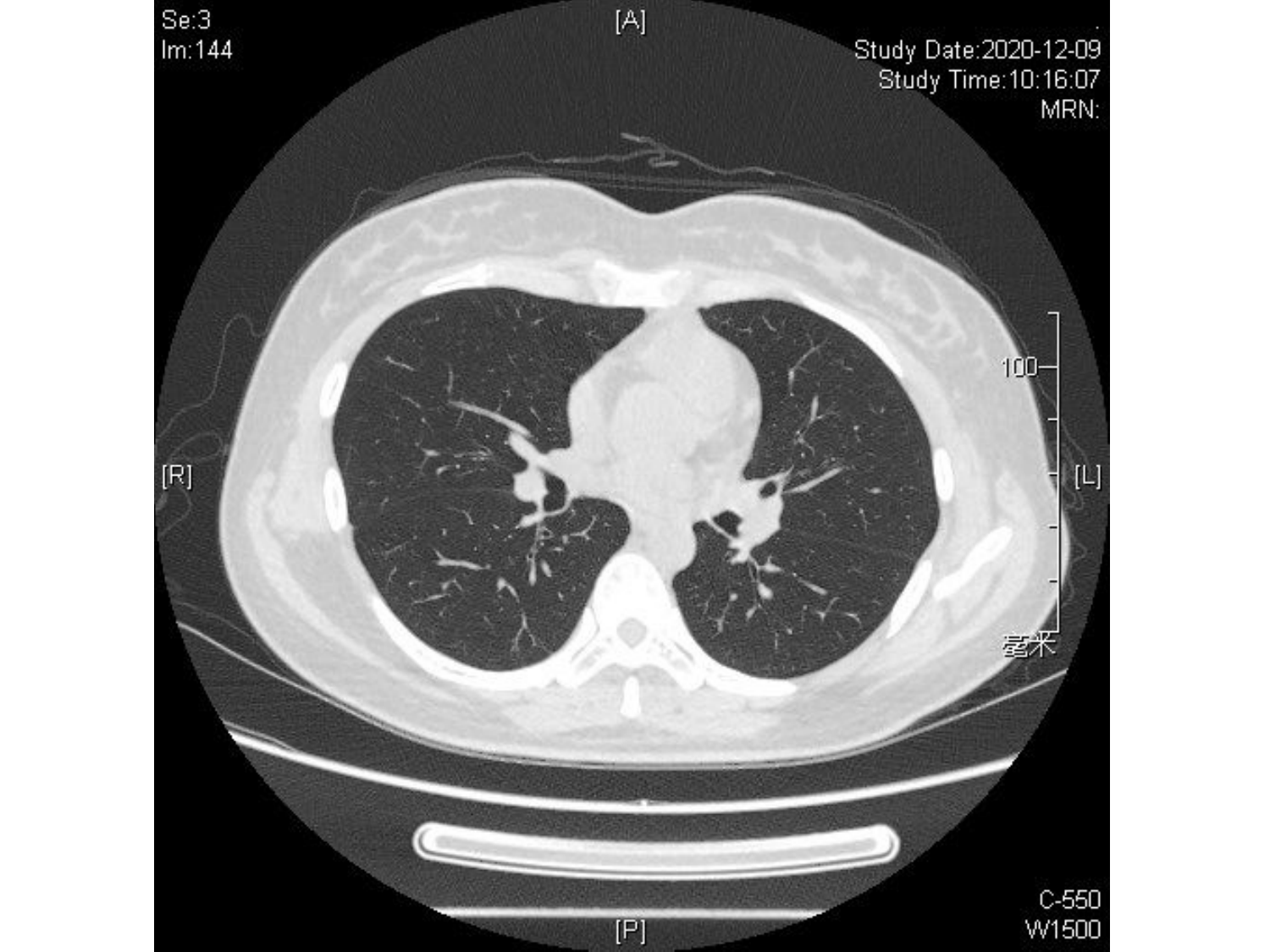

#

## Slide 10
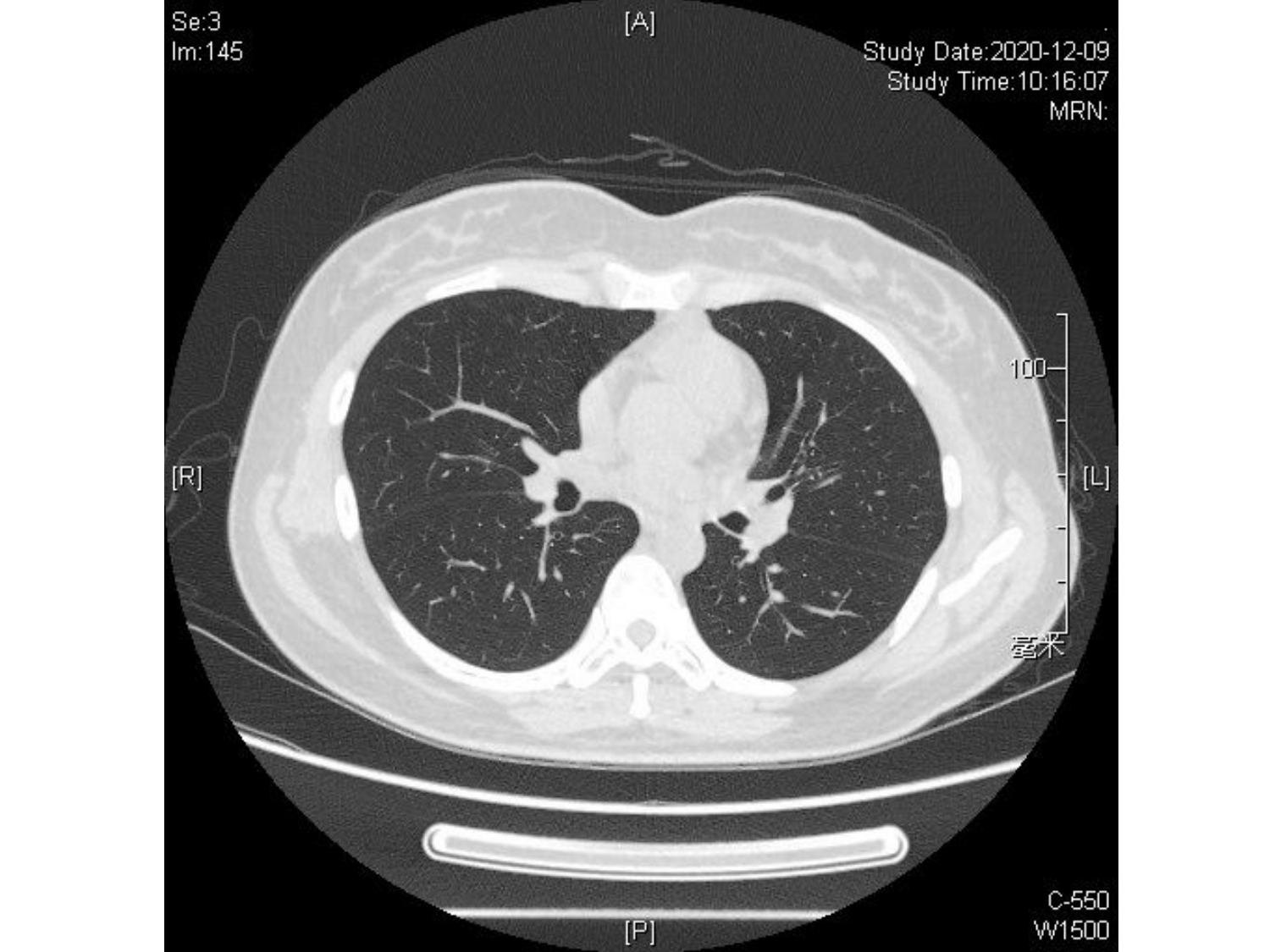

#

## Slide 11
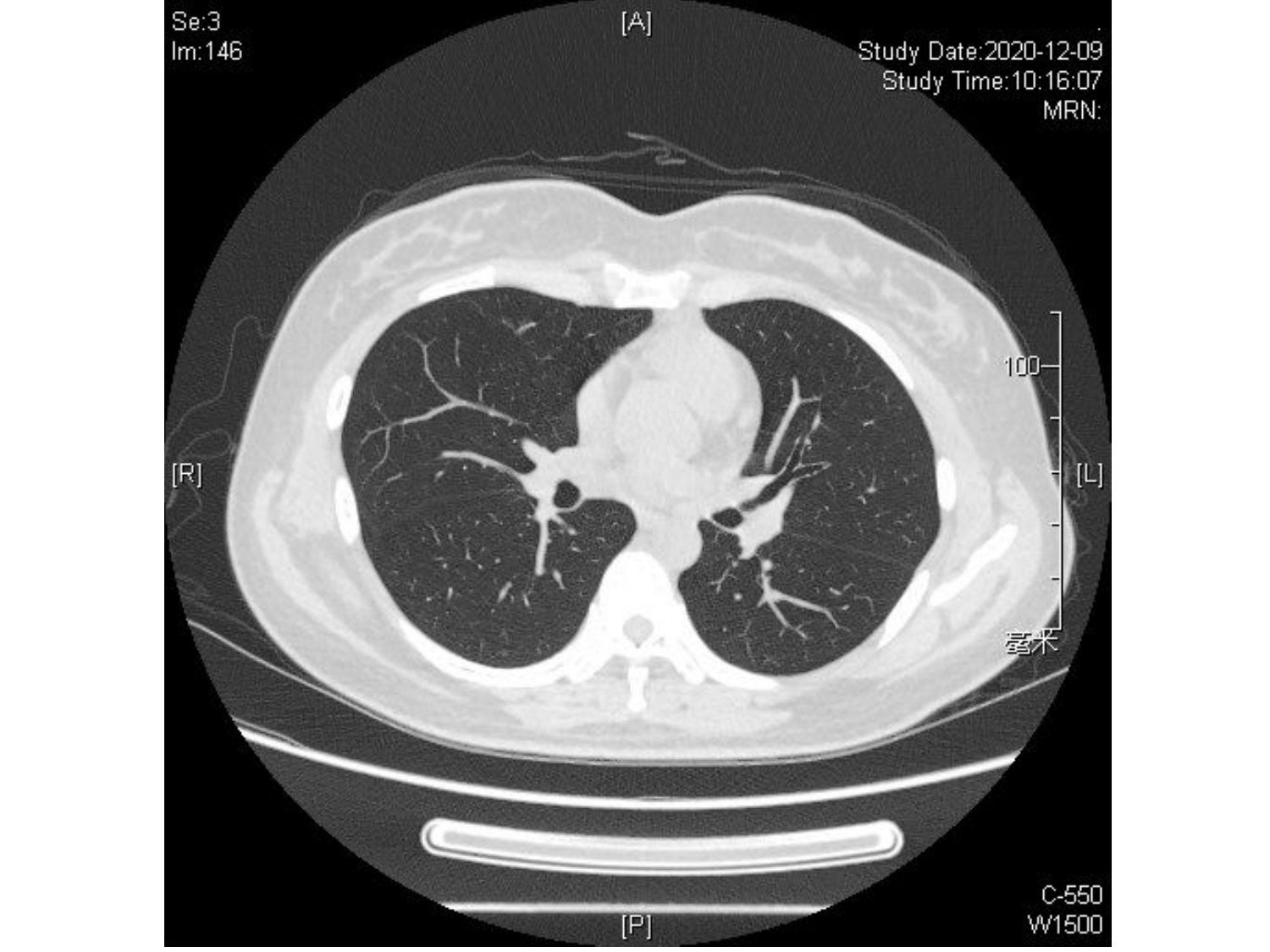

#

## Slide 12
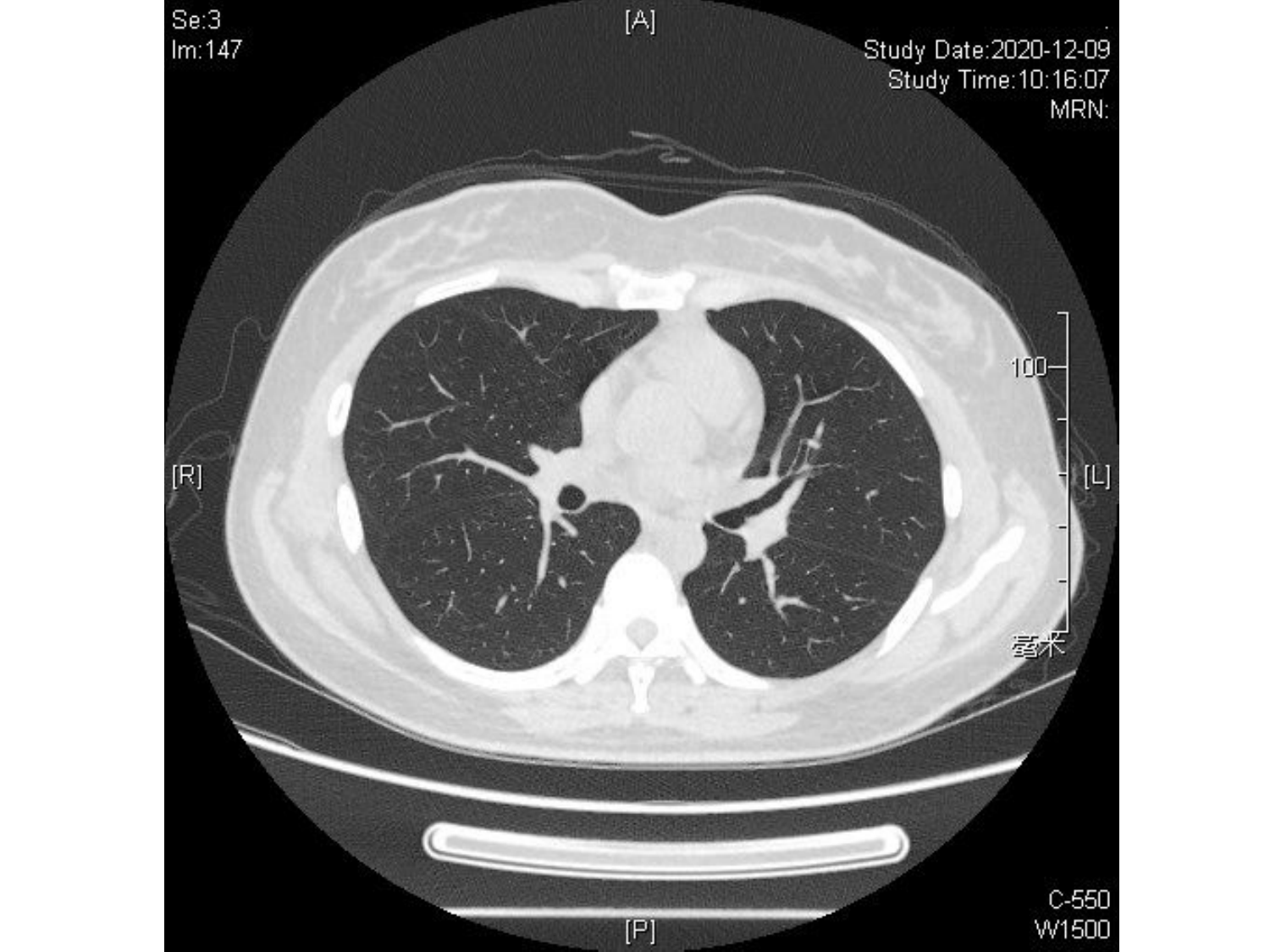

#

## Slide 13
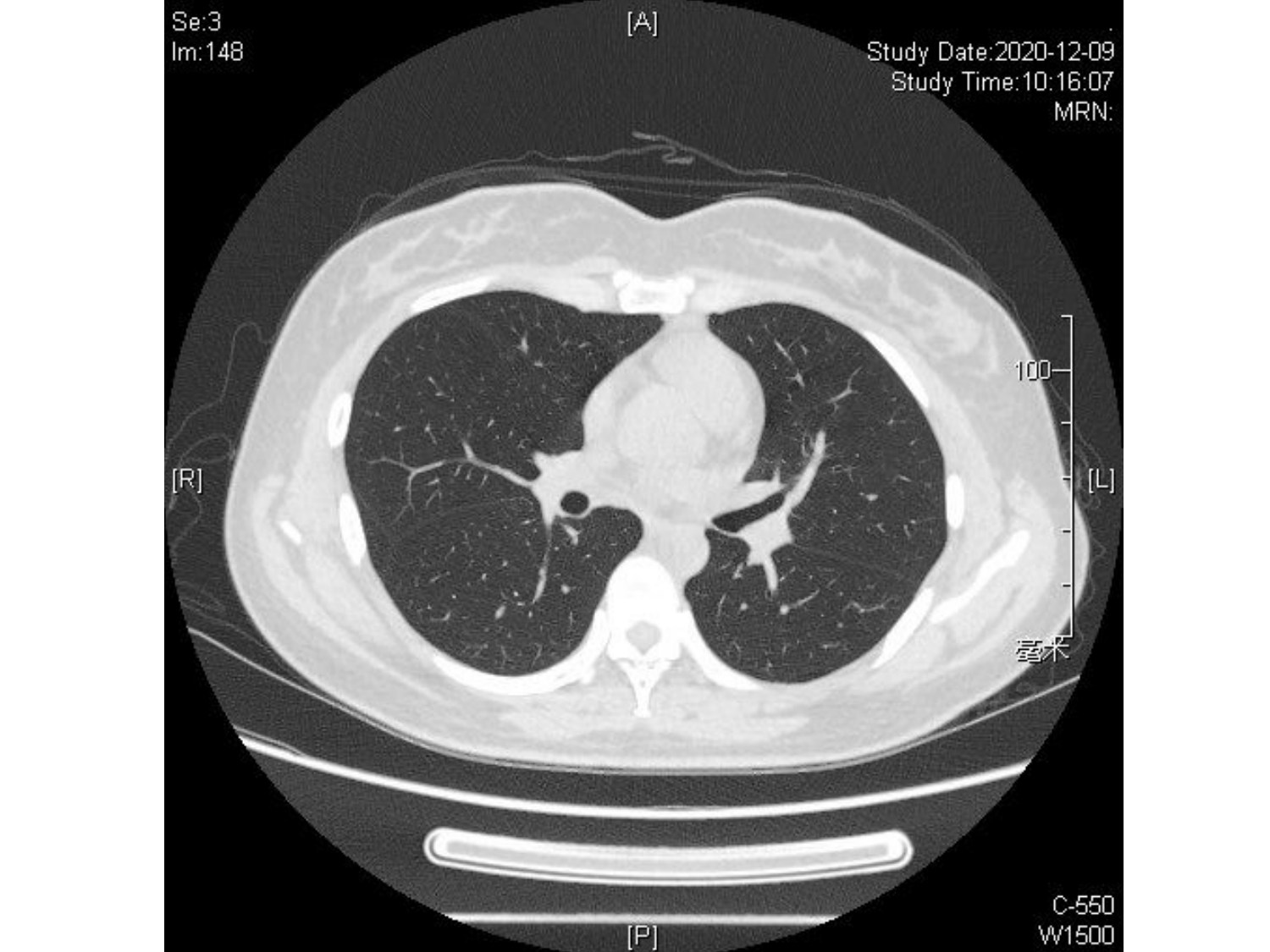

#

## Slide 14
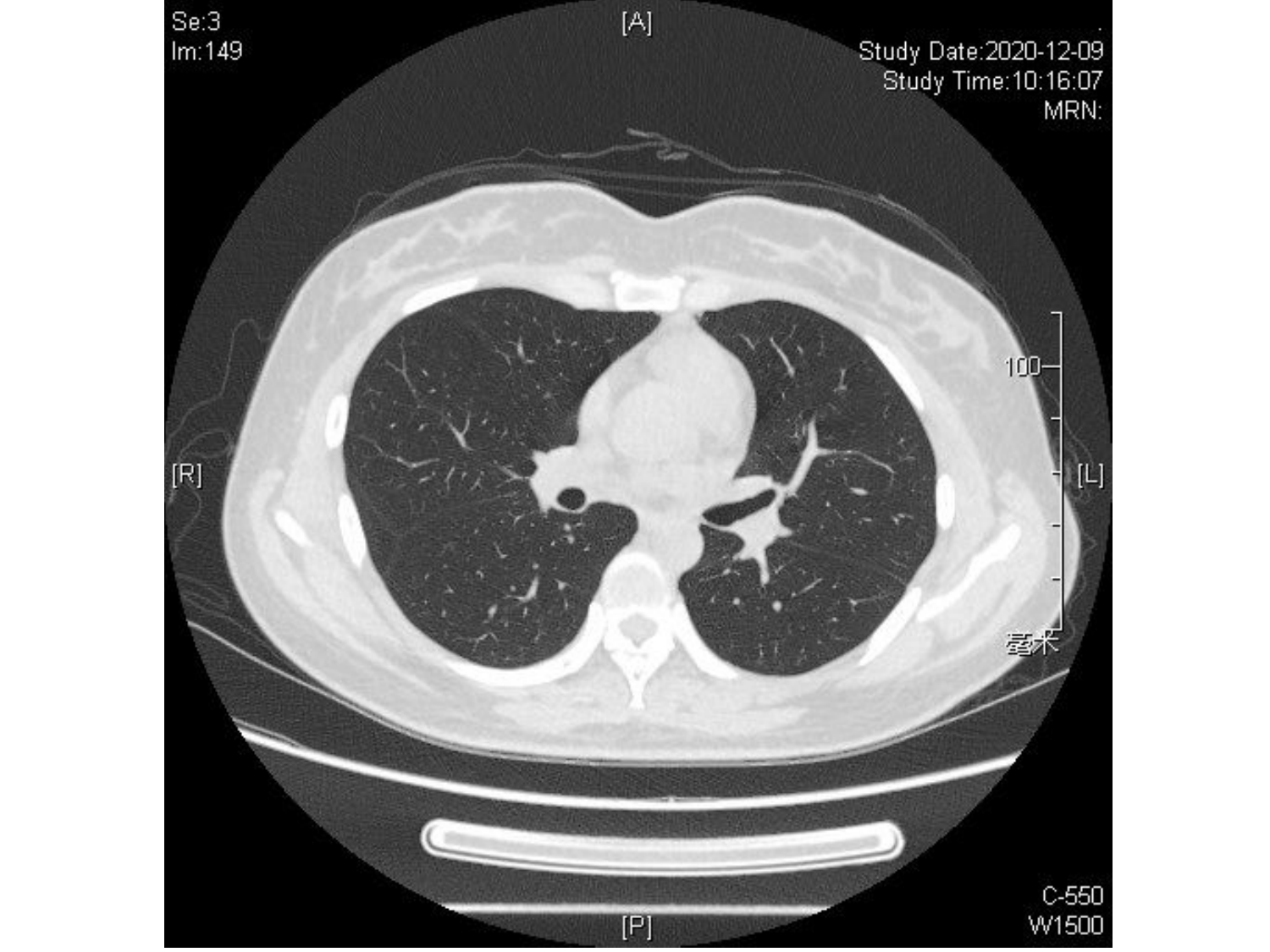

#

## Slide 15
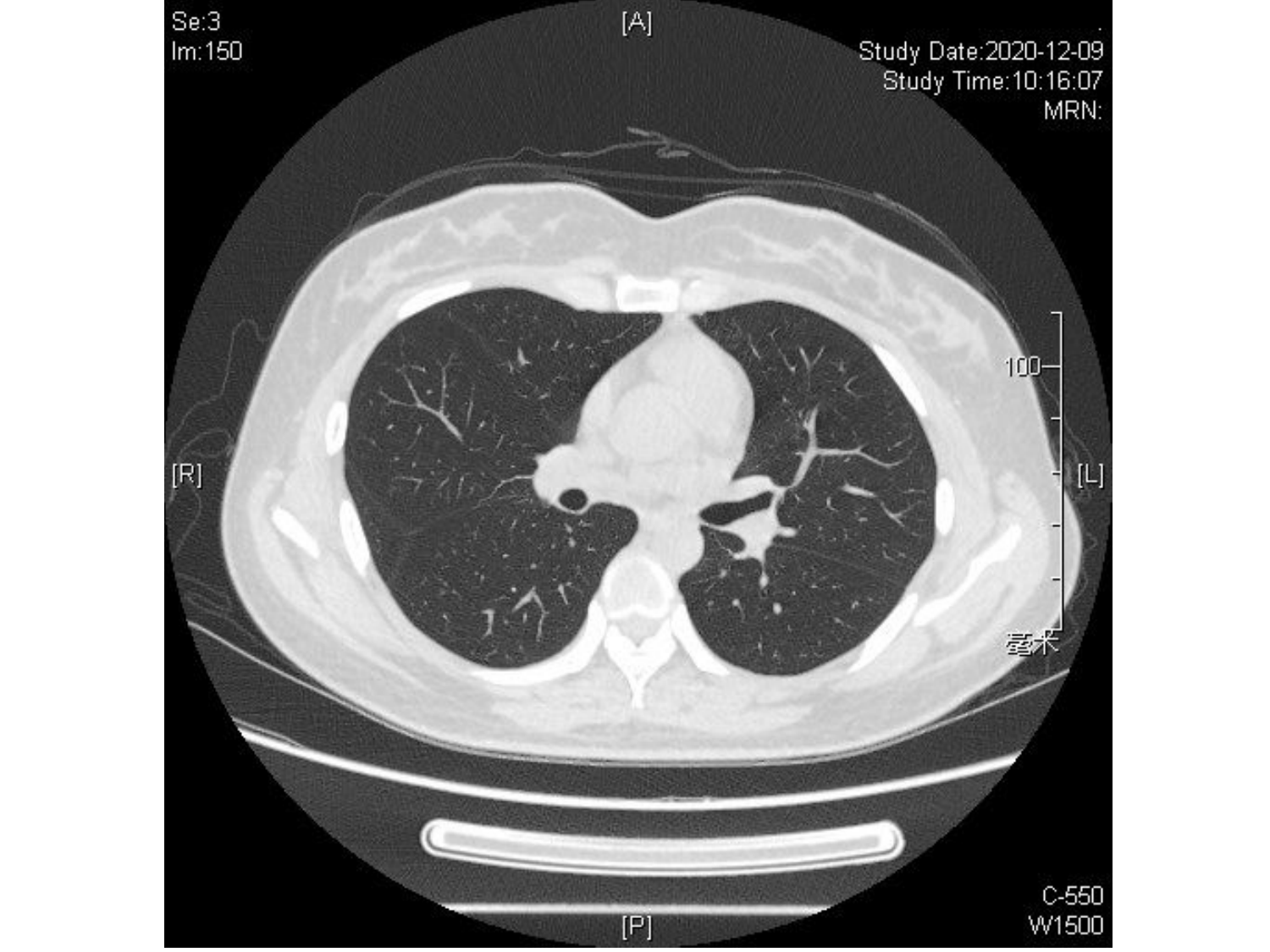

#

## Slide 16
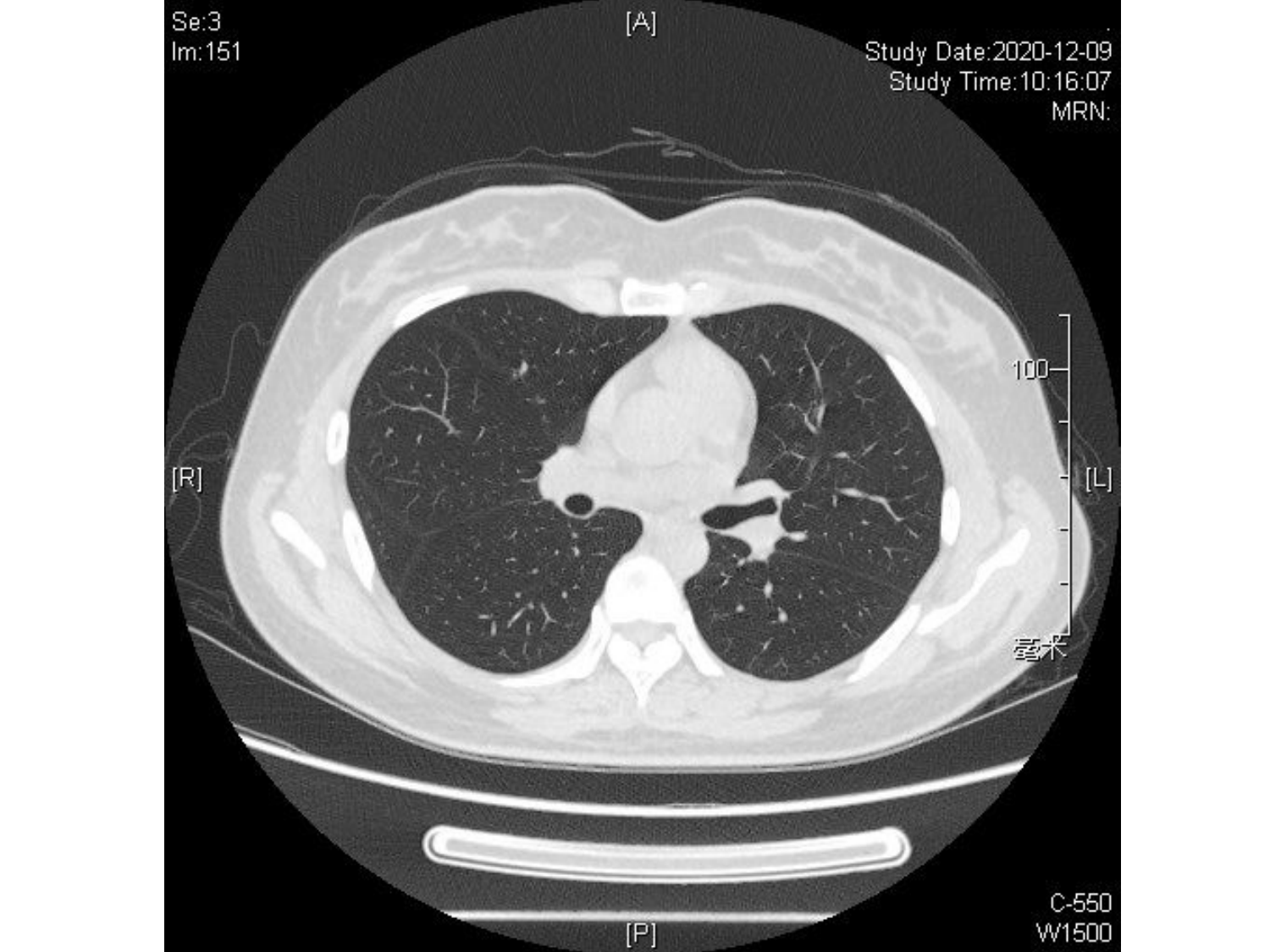

#

## Slide 17
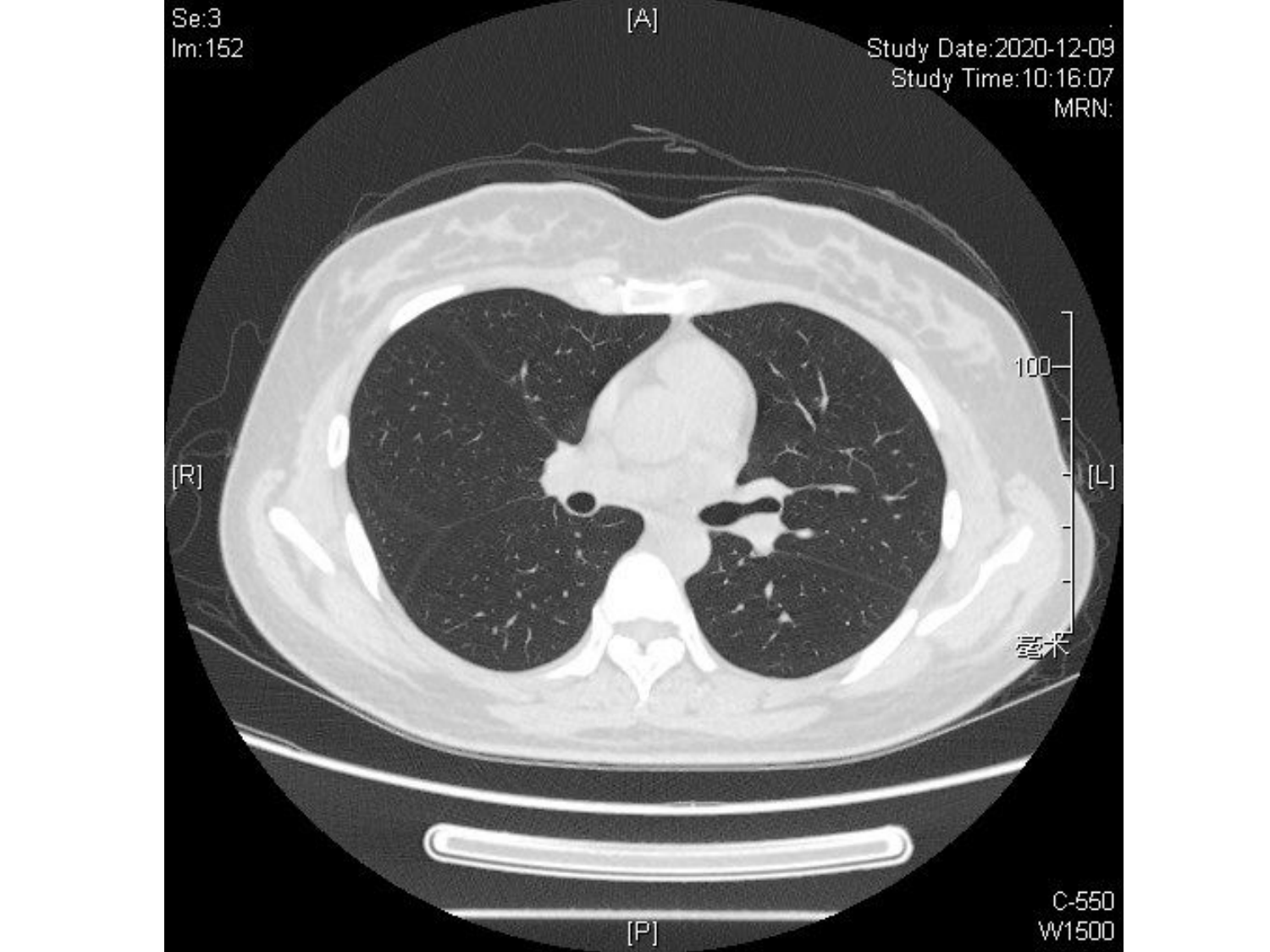

#

## Slide 18
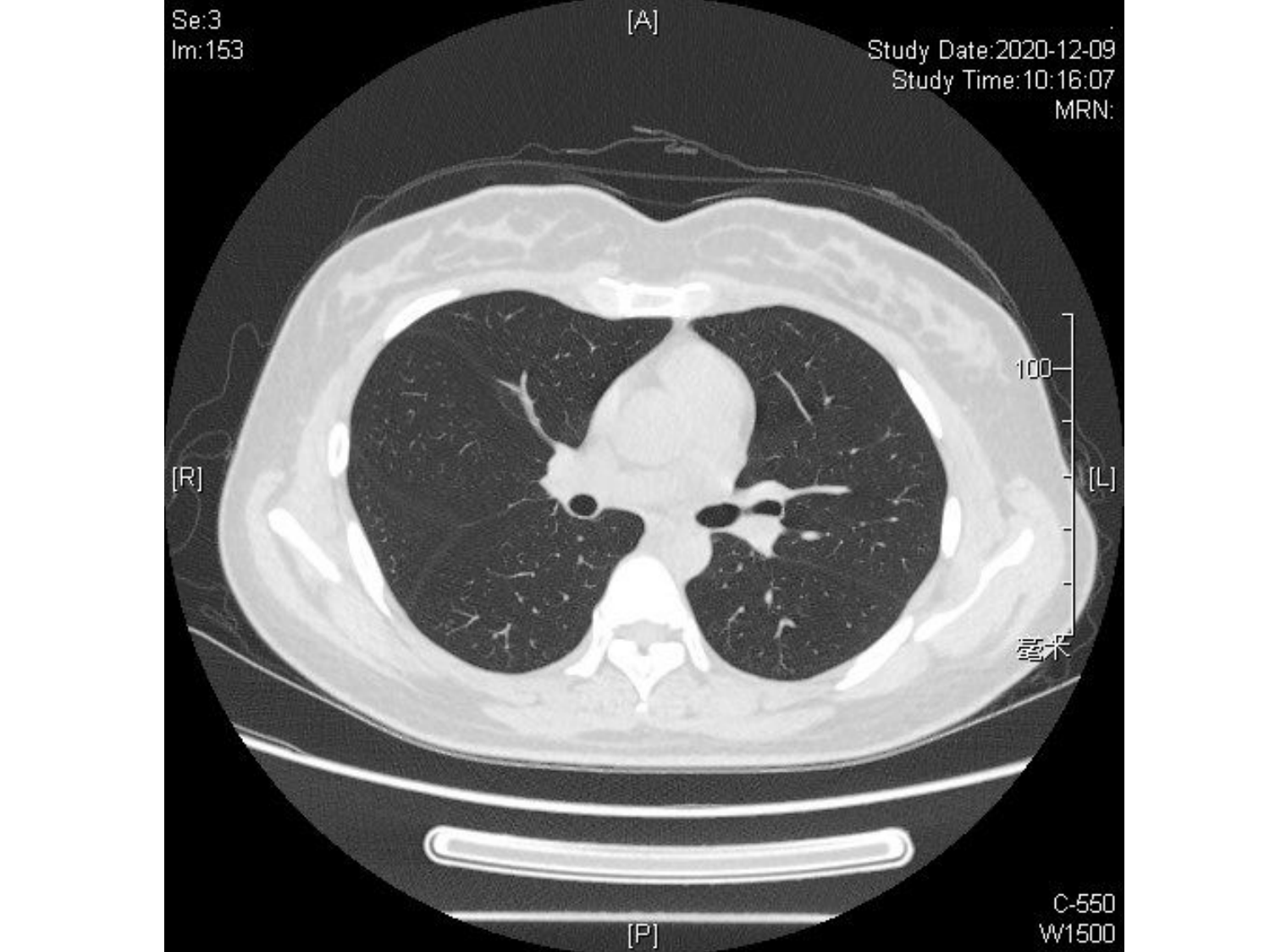

#

## Slide 19
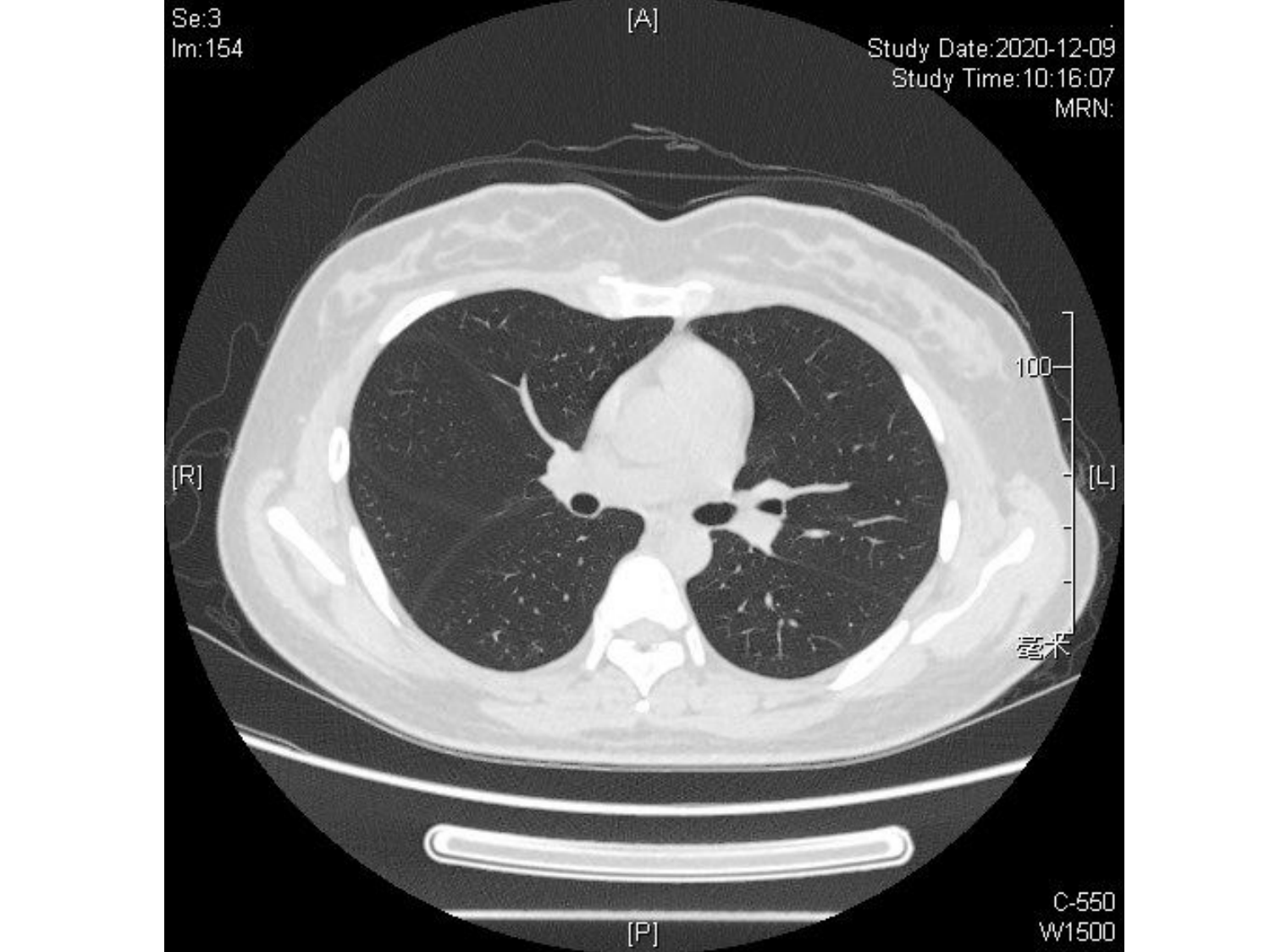

#

## Slide 20
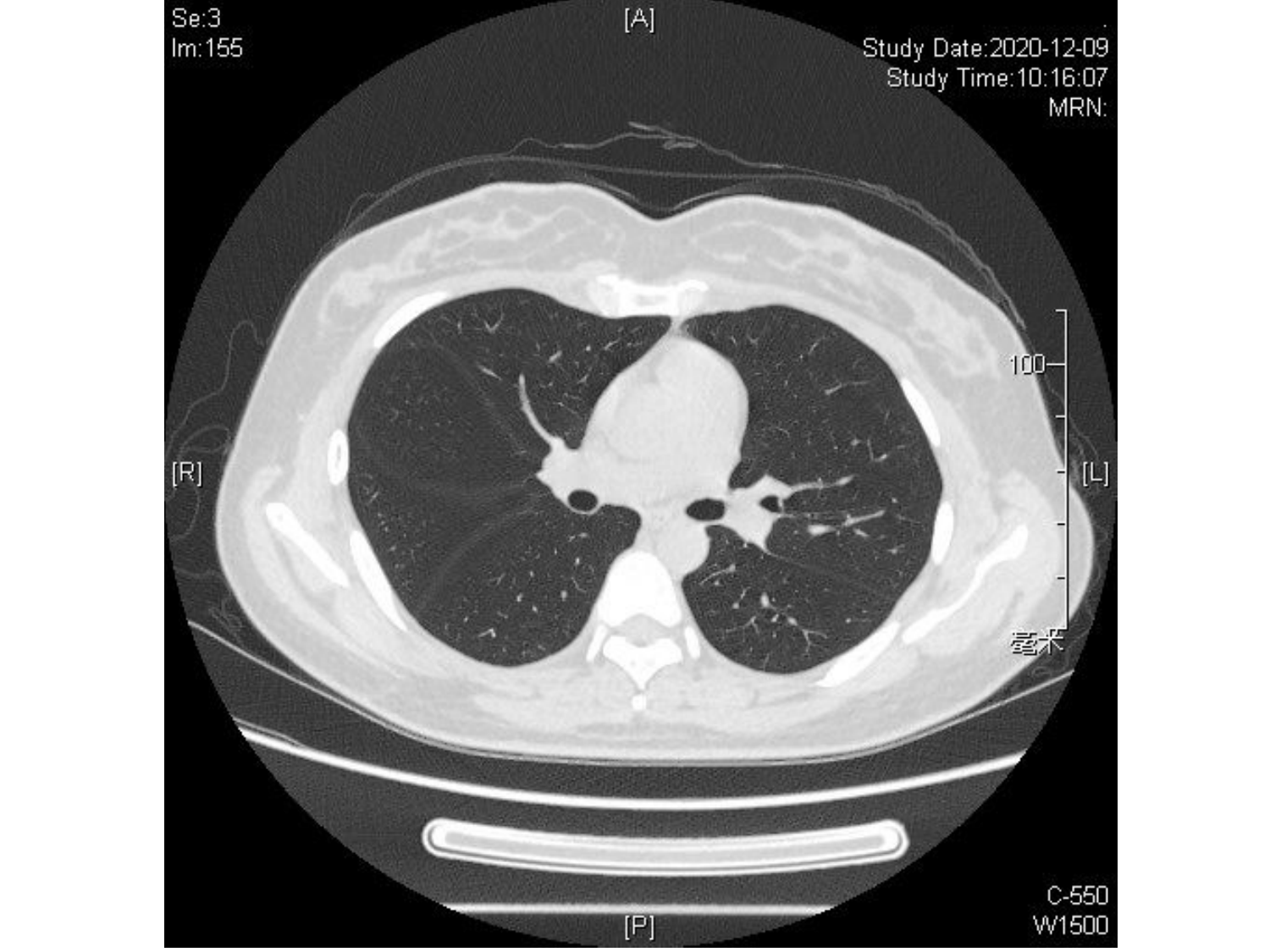

#

## Slide 21
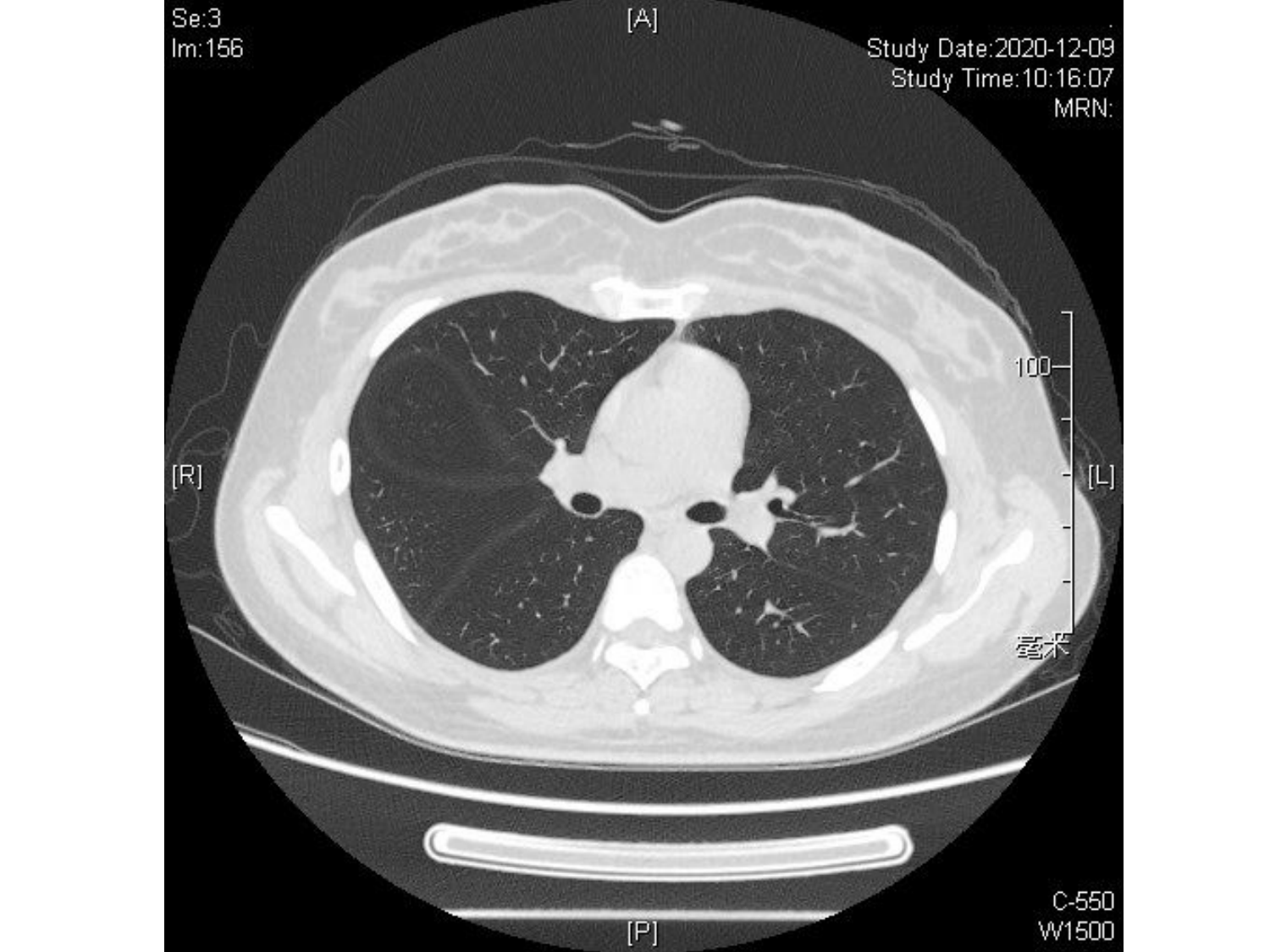

#

## Slide 22
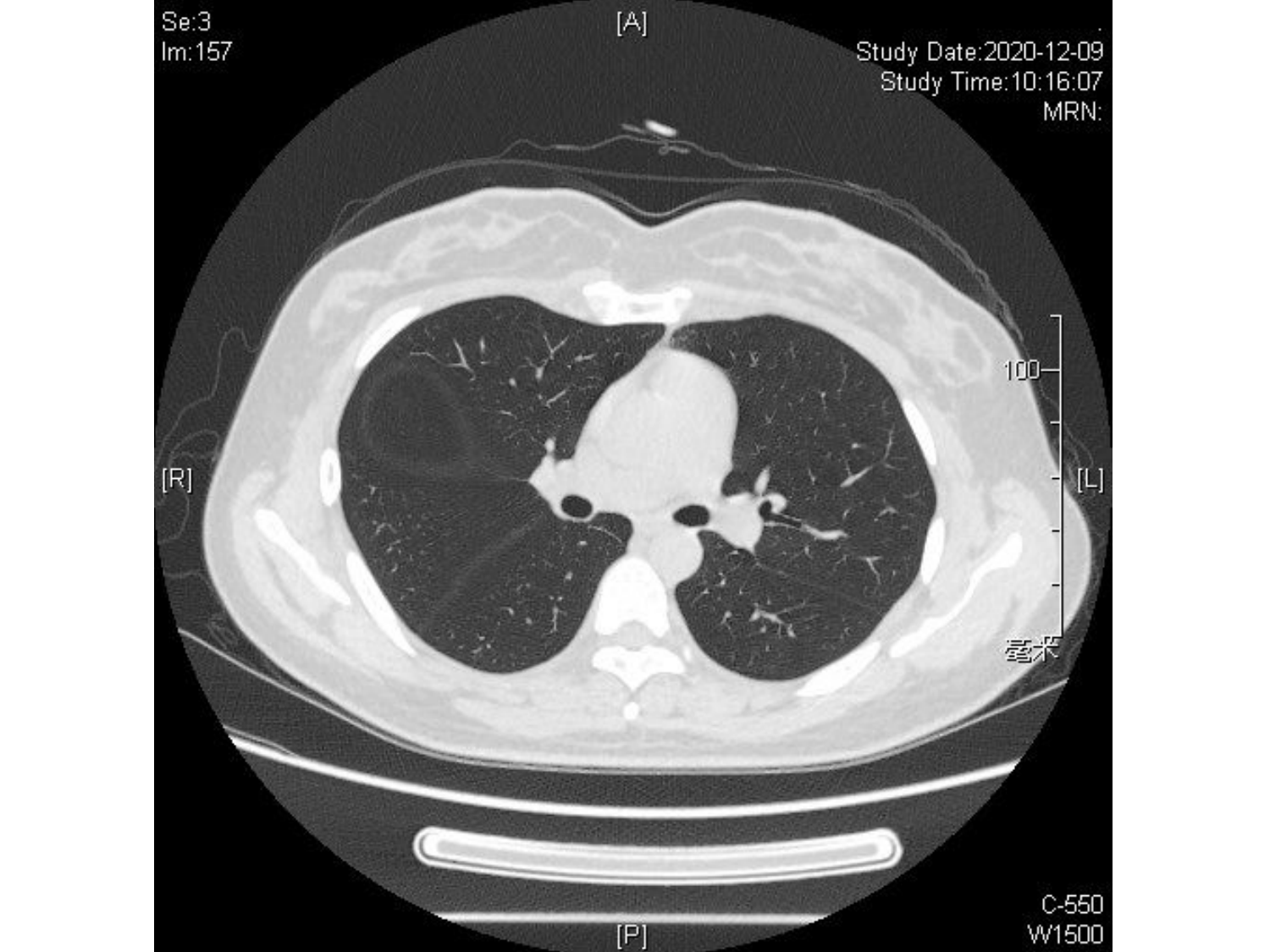

#

## Slide 23
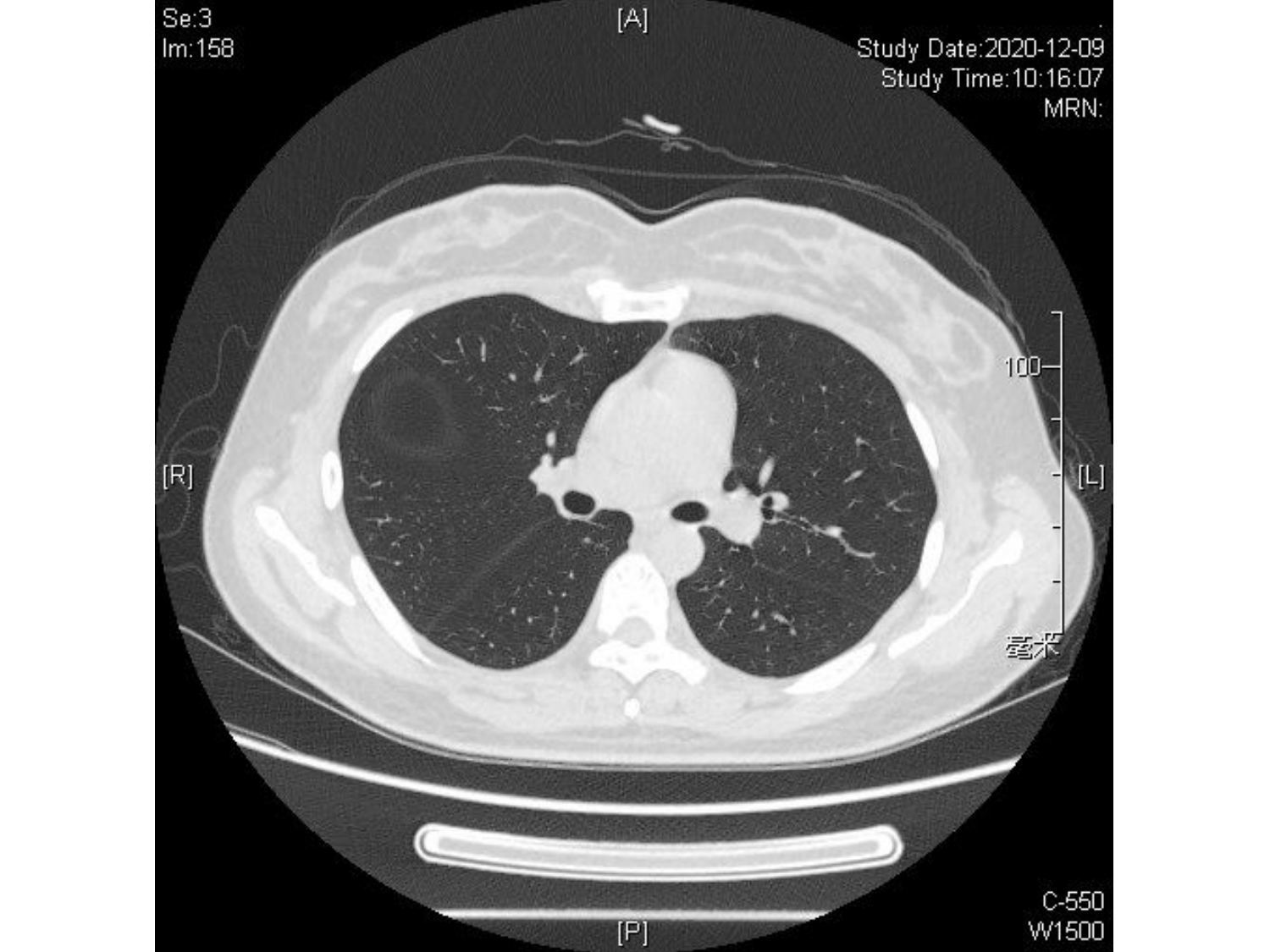

#

## Slide 24
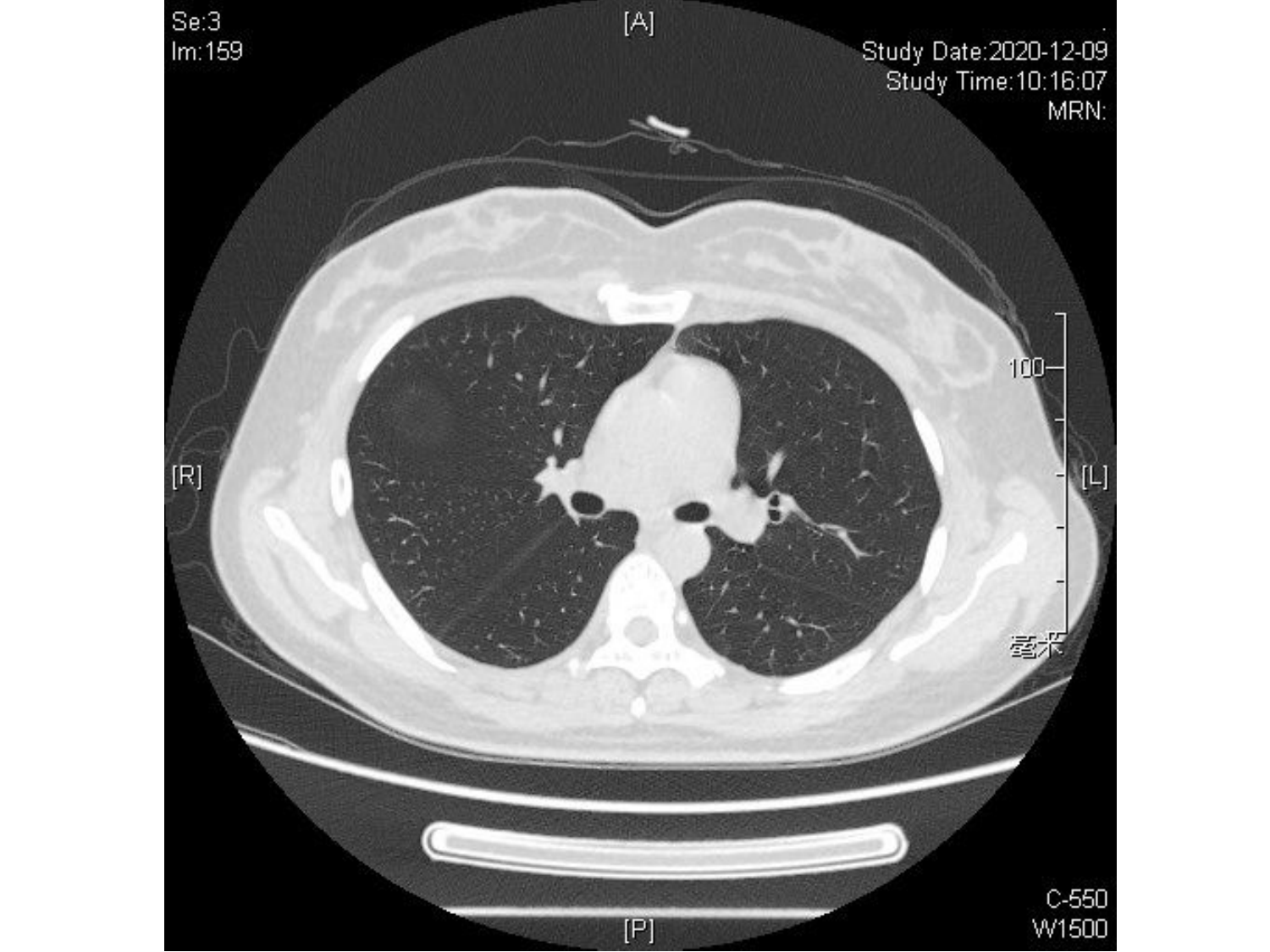

#

## Slide 25
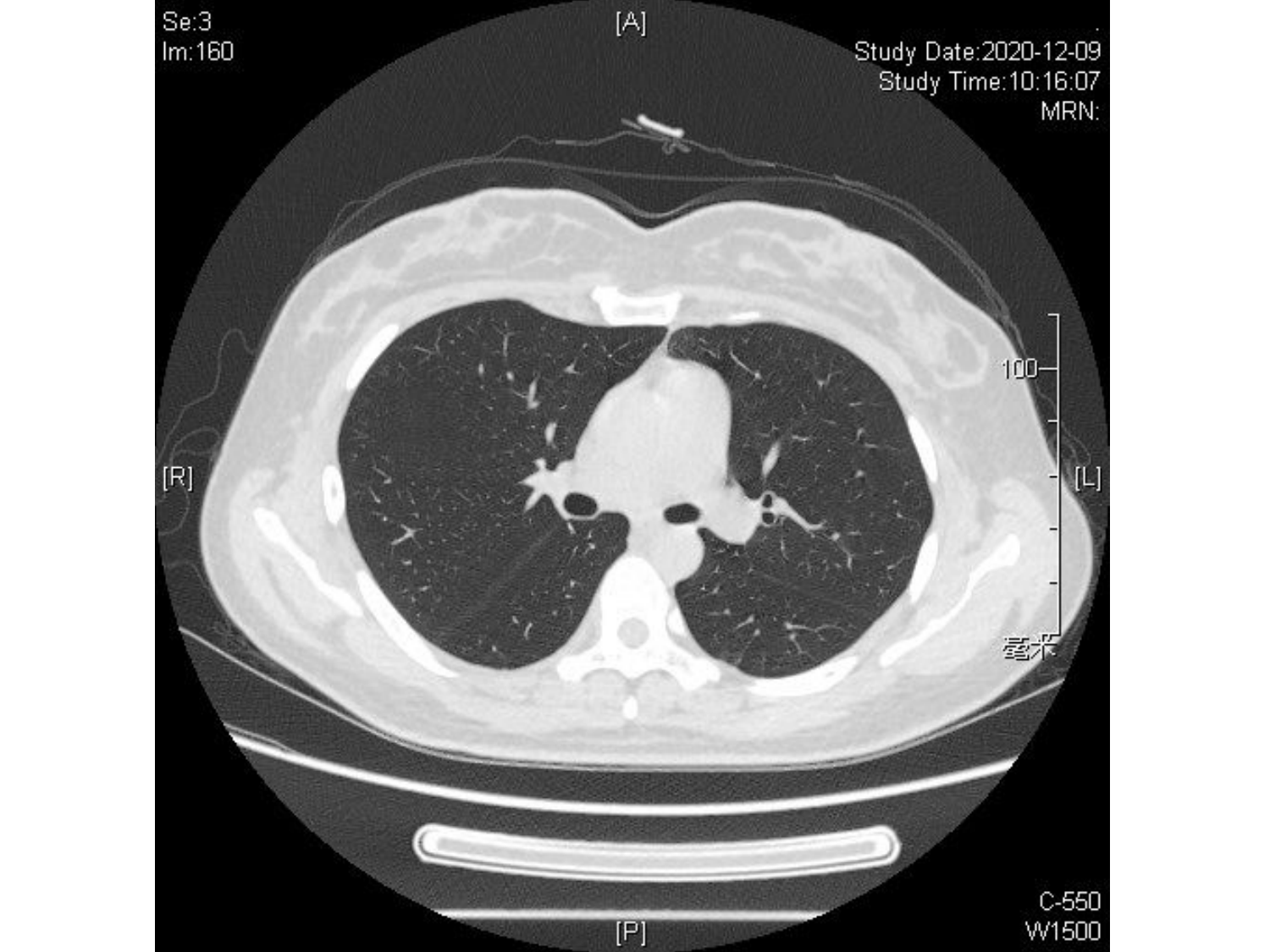

#

## Slide 26
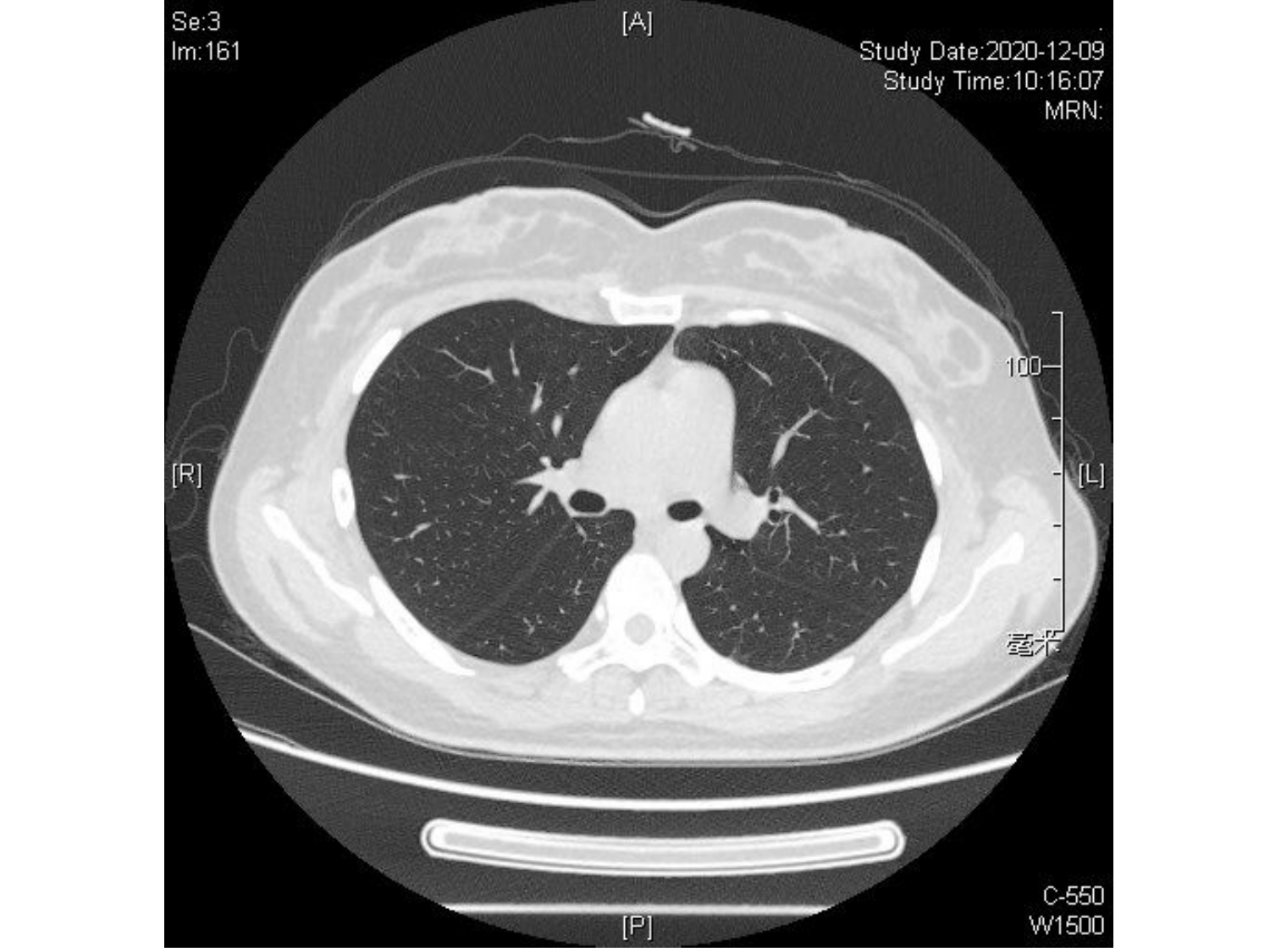

#

## Slide 27
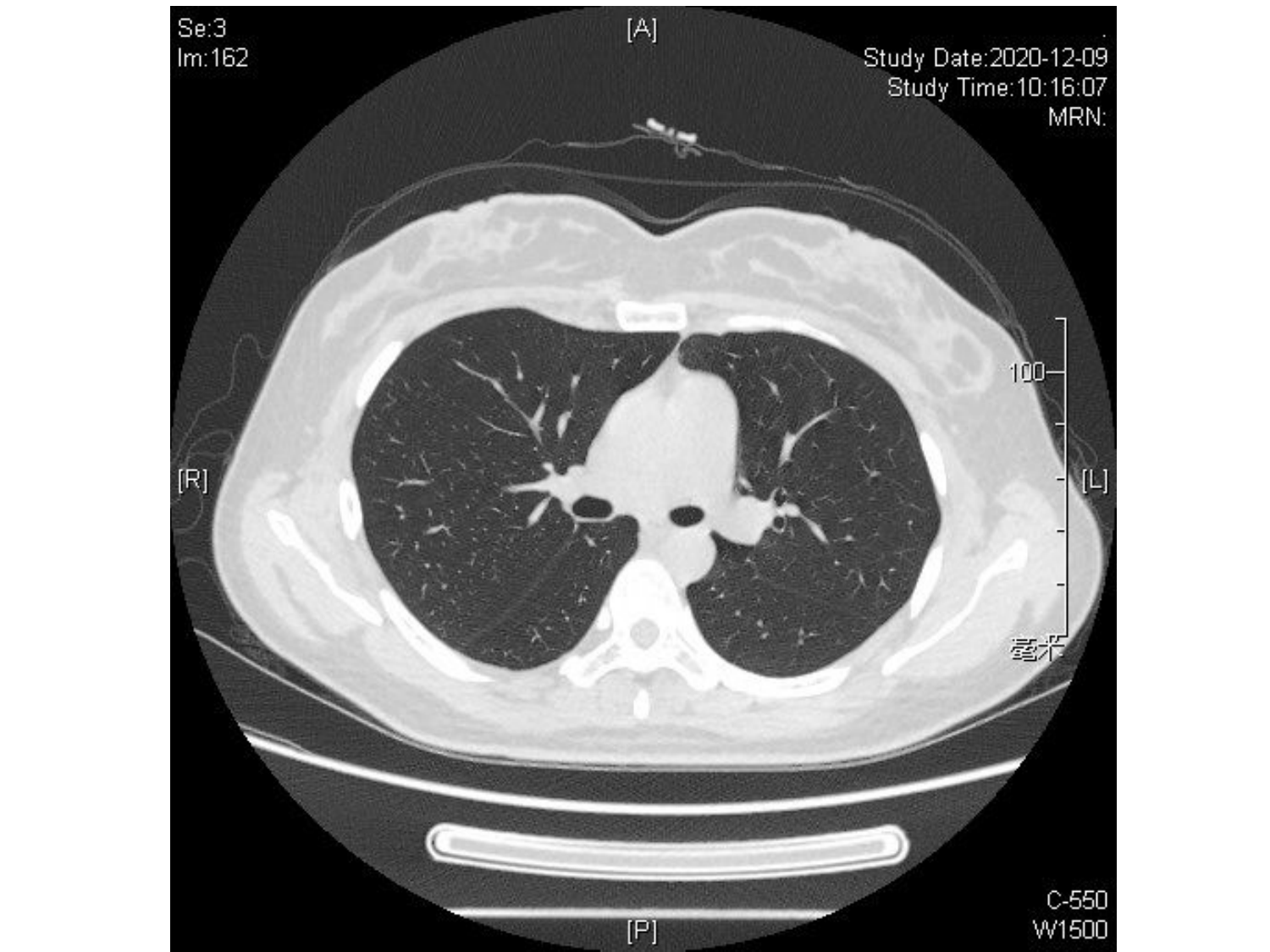

#

## Slide 28
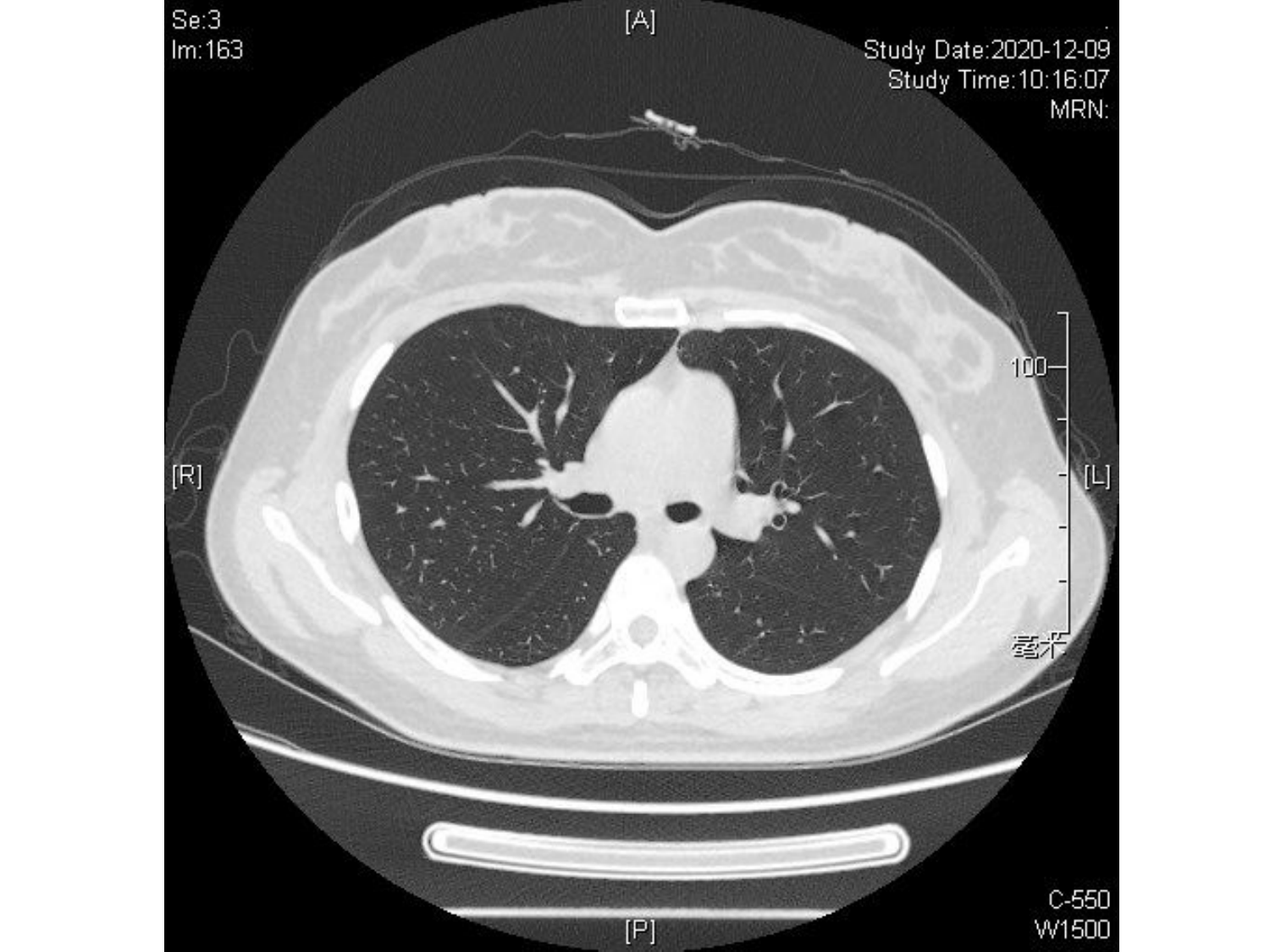

#

## Slide 29
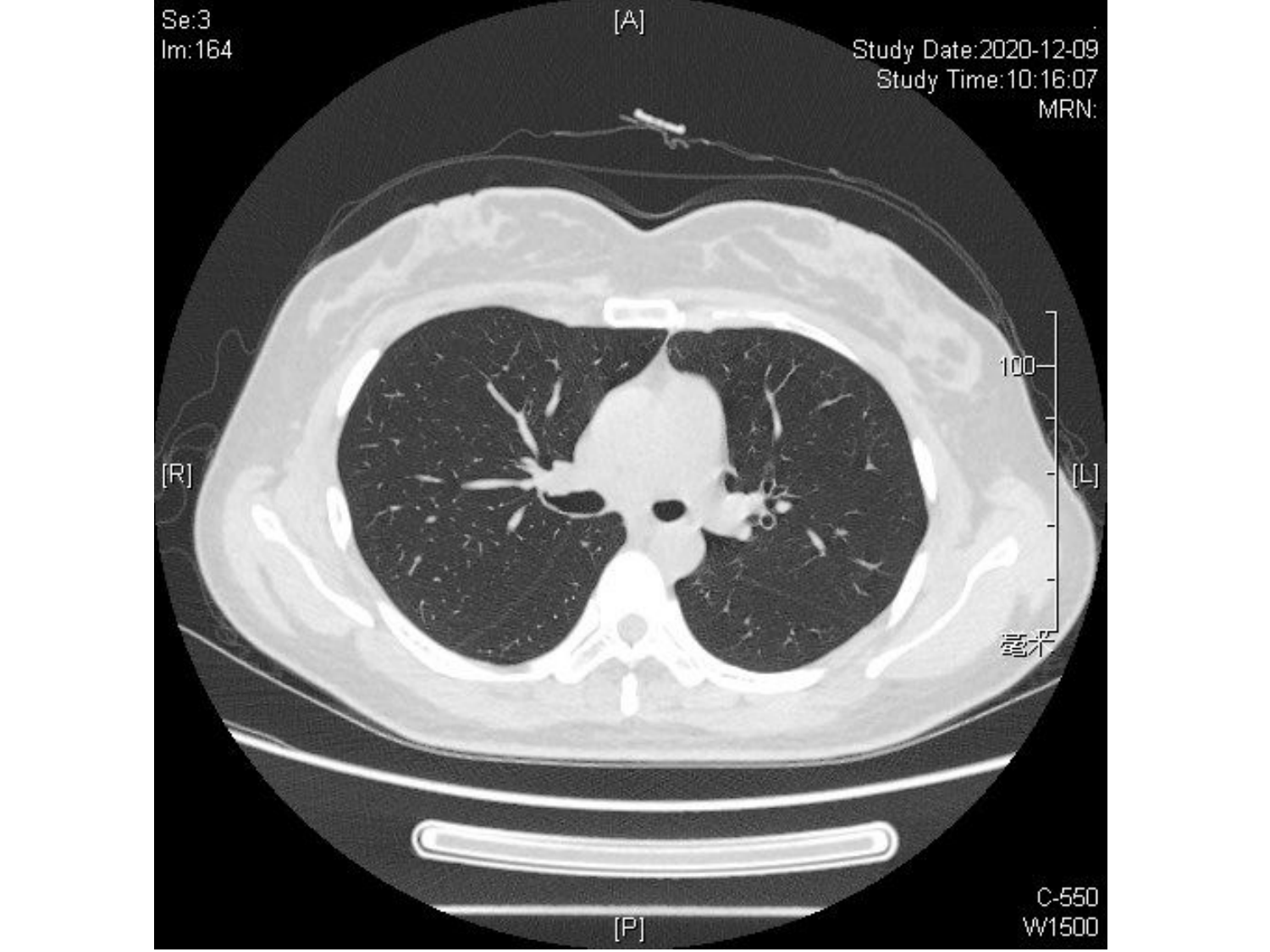

#

## Slide 30
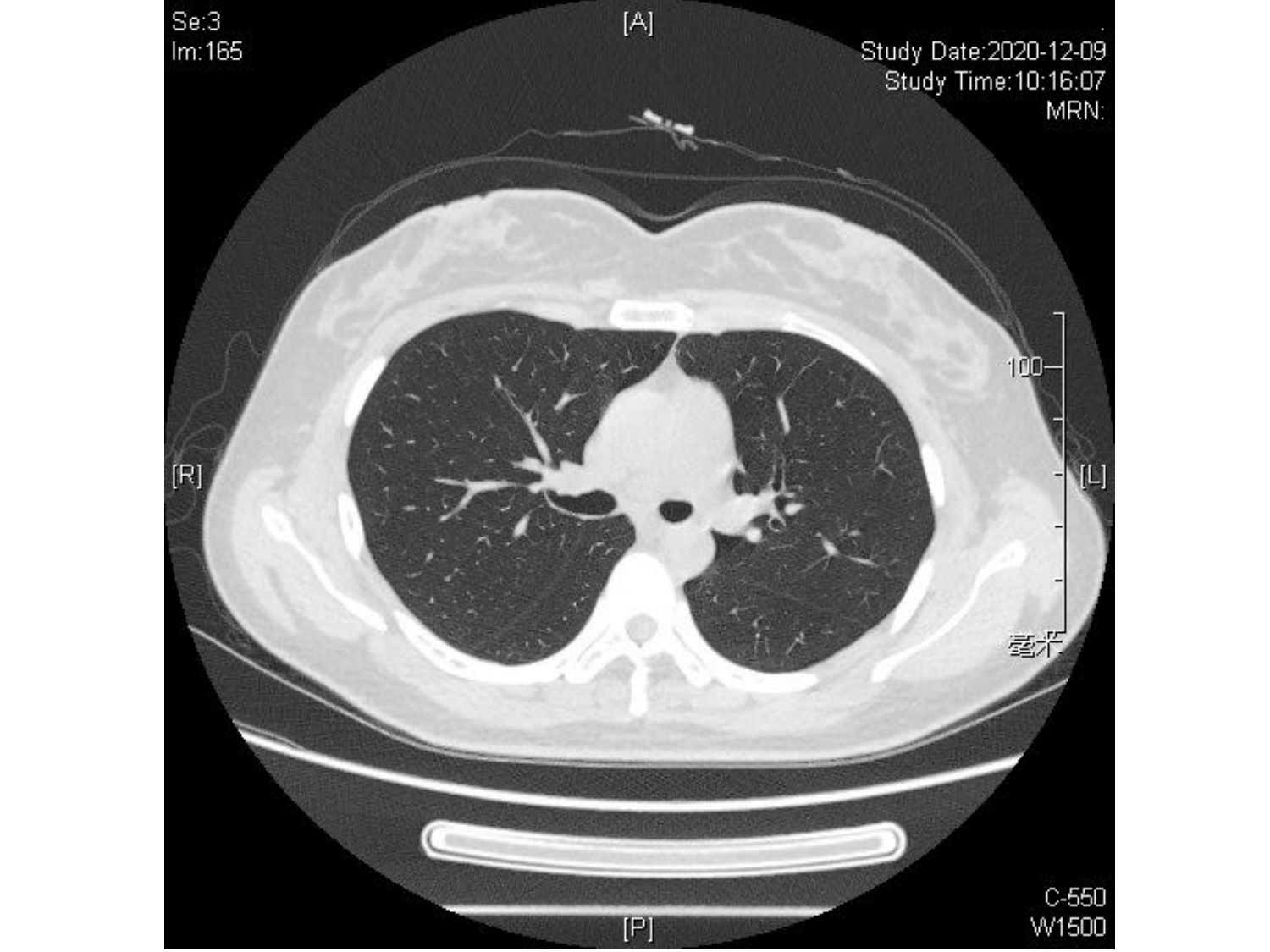

#

## Slide 31
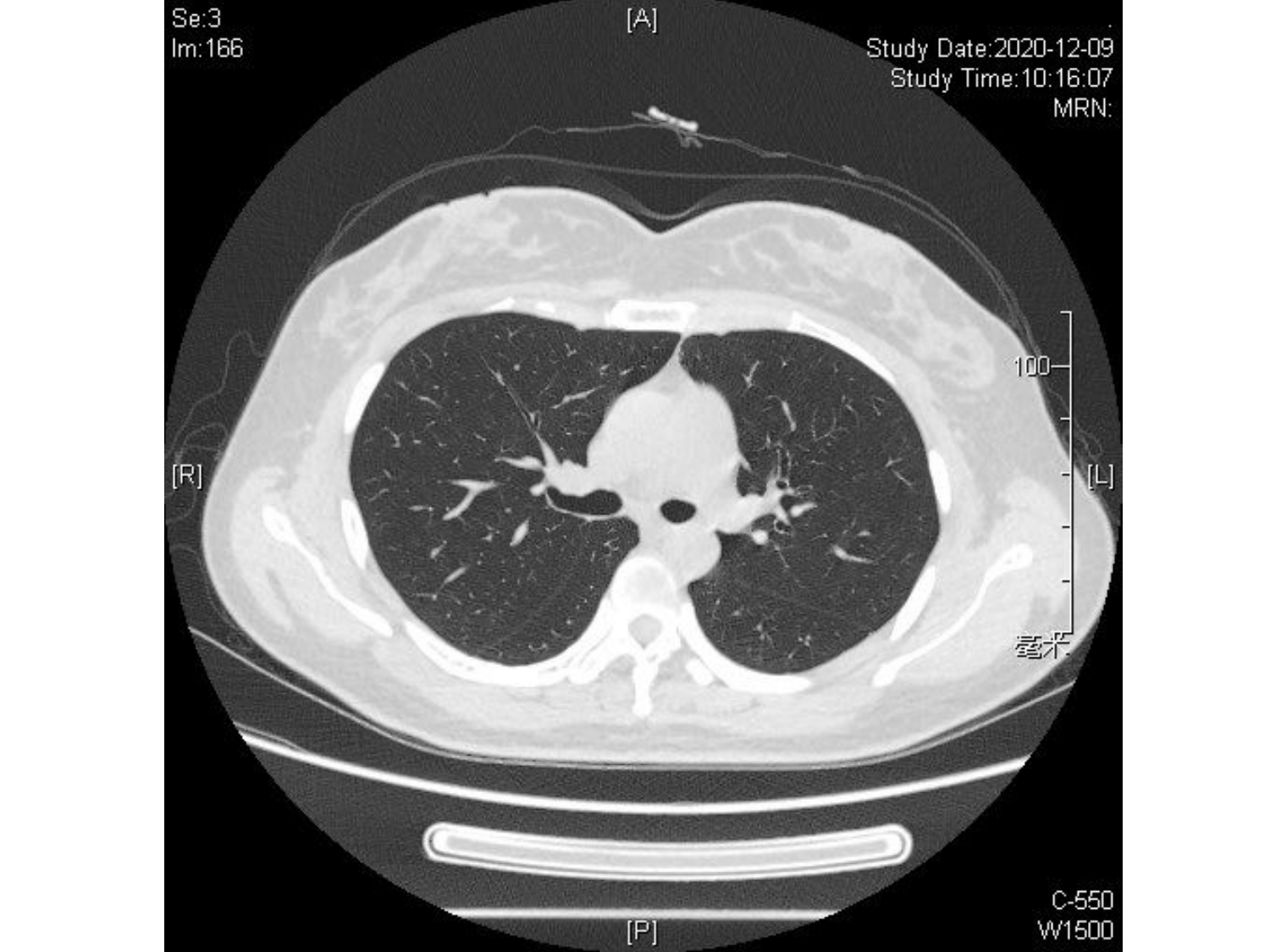

#

## Slide 32
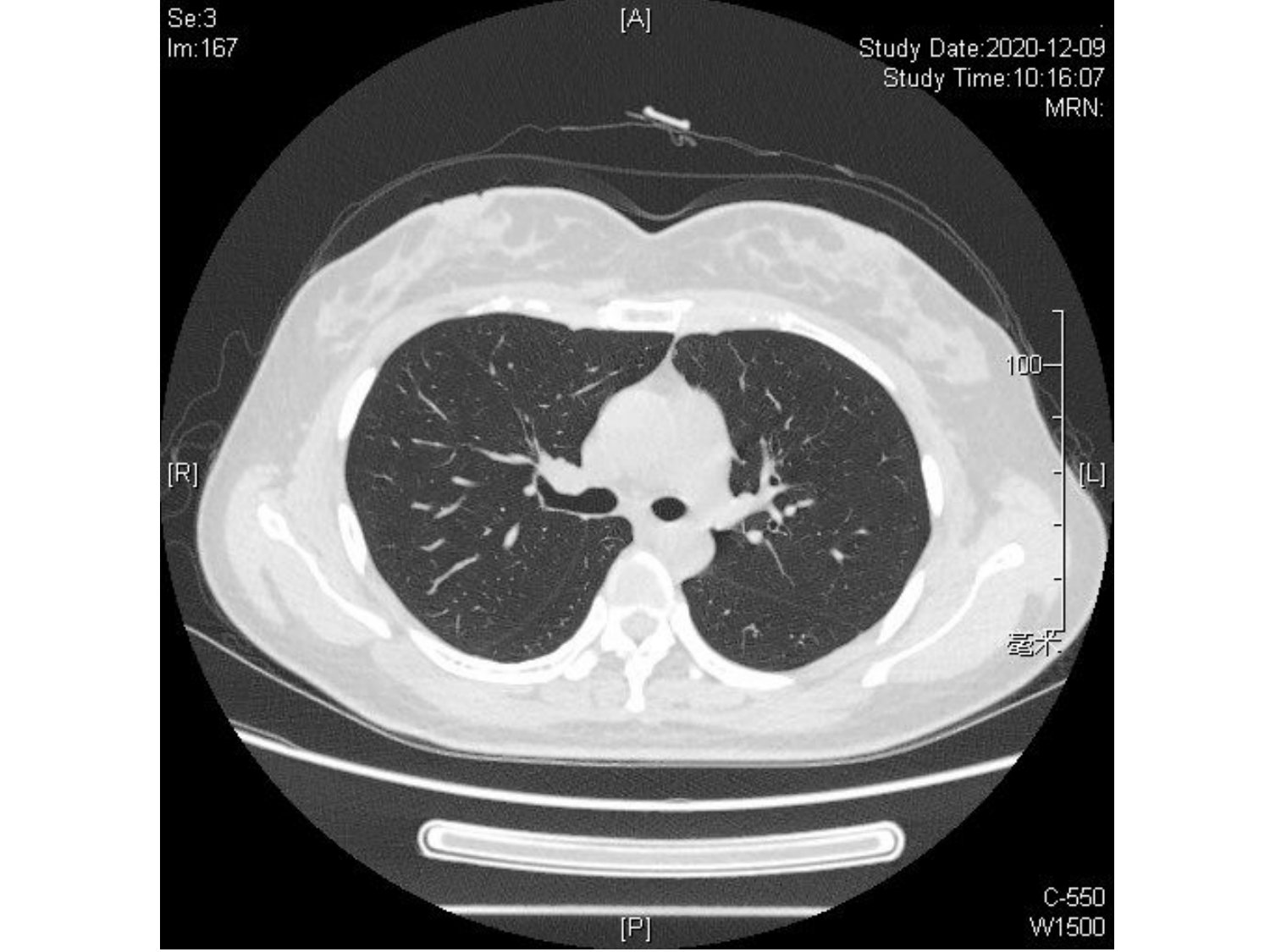

#

## Slide 33
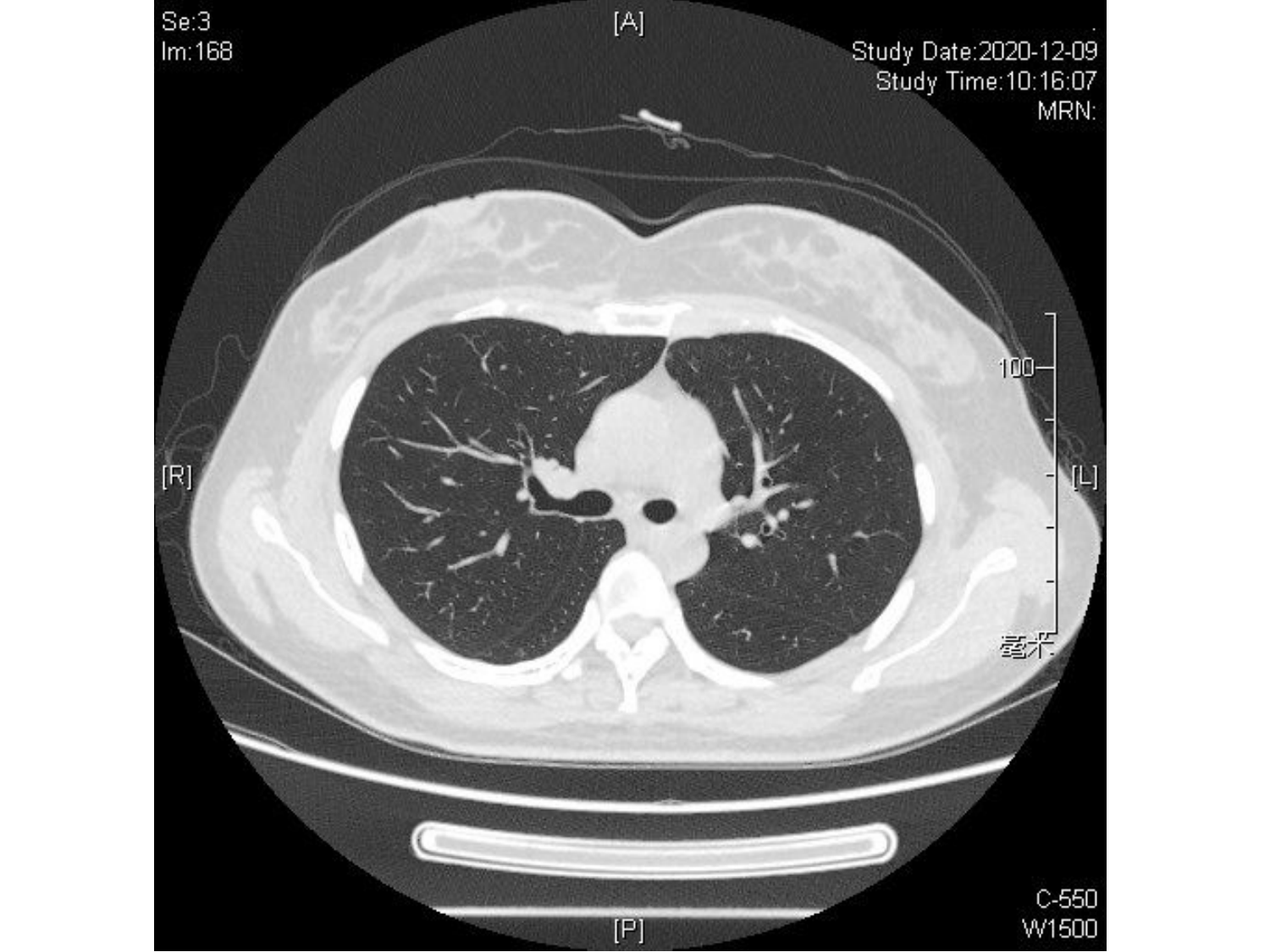

#

## Slide 34
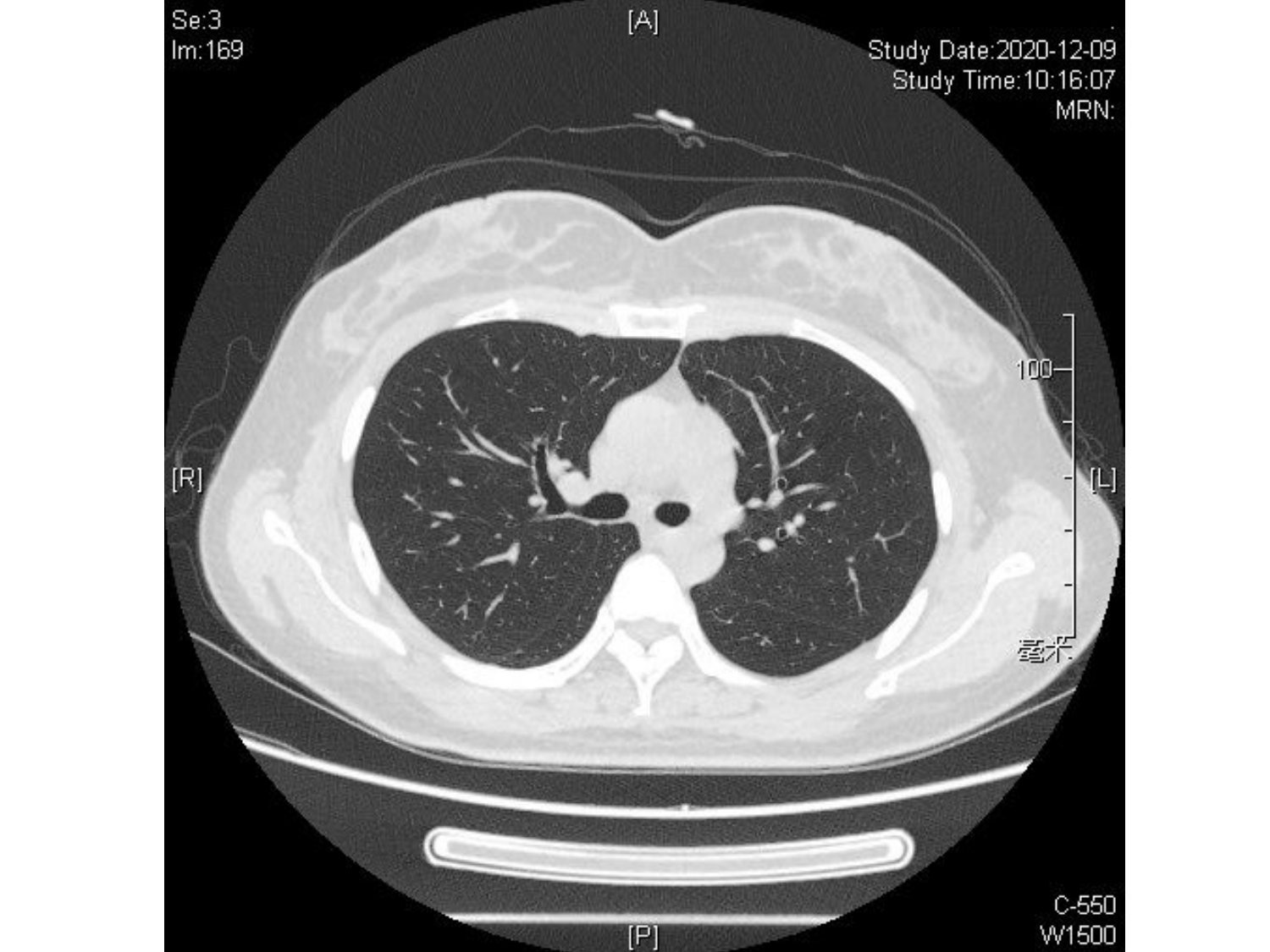

#

## Slide 35
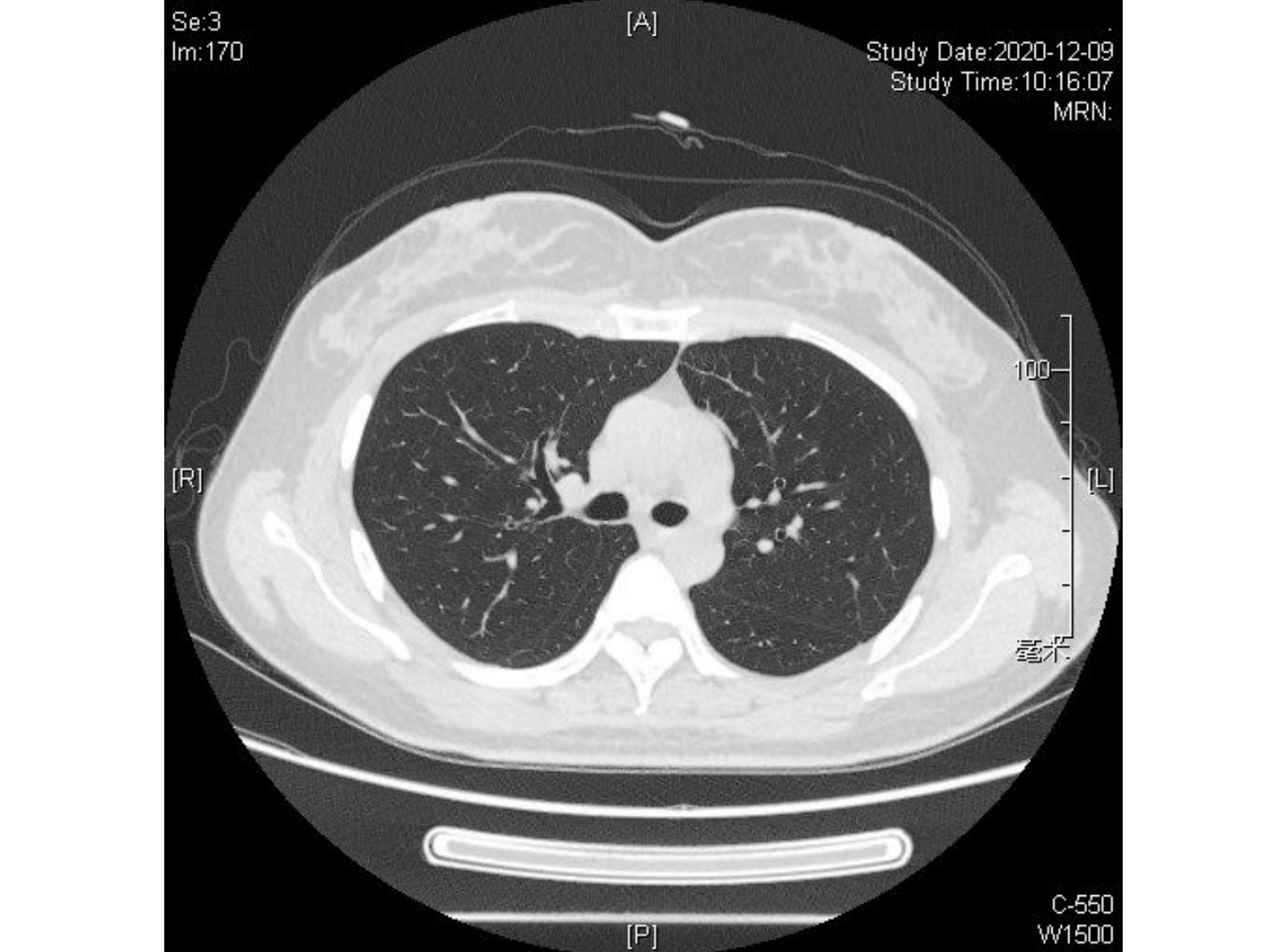

#

## Slide 36
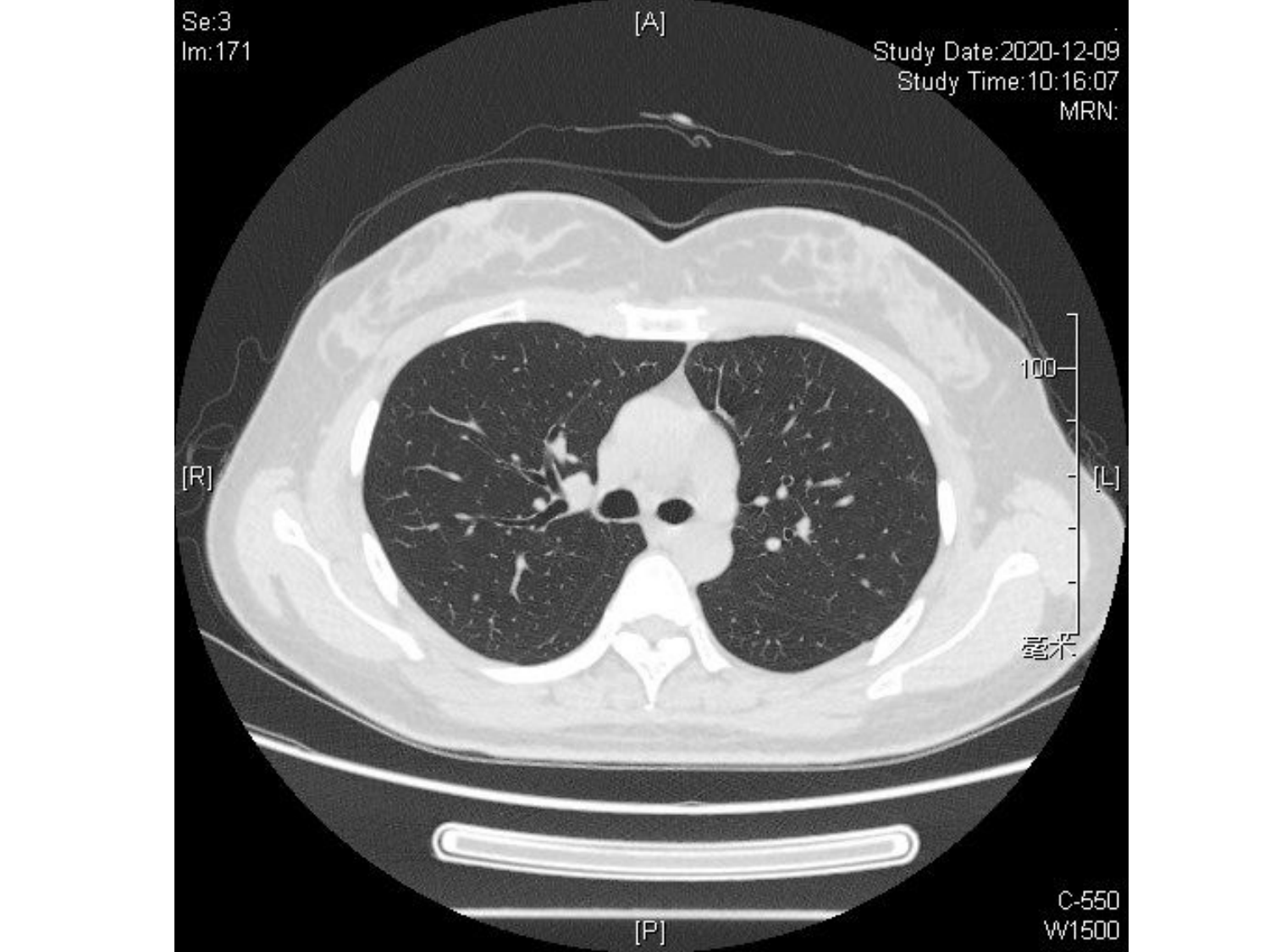

#

## Slide 37
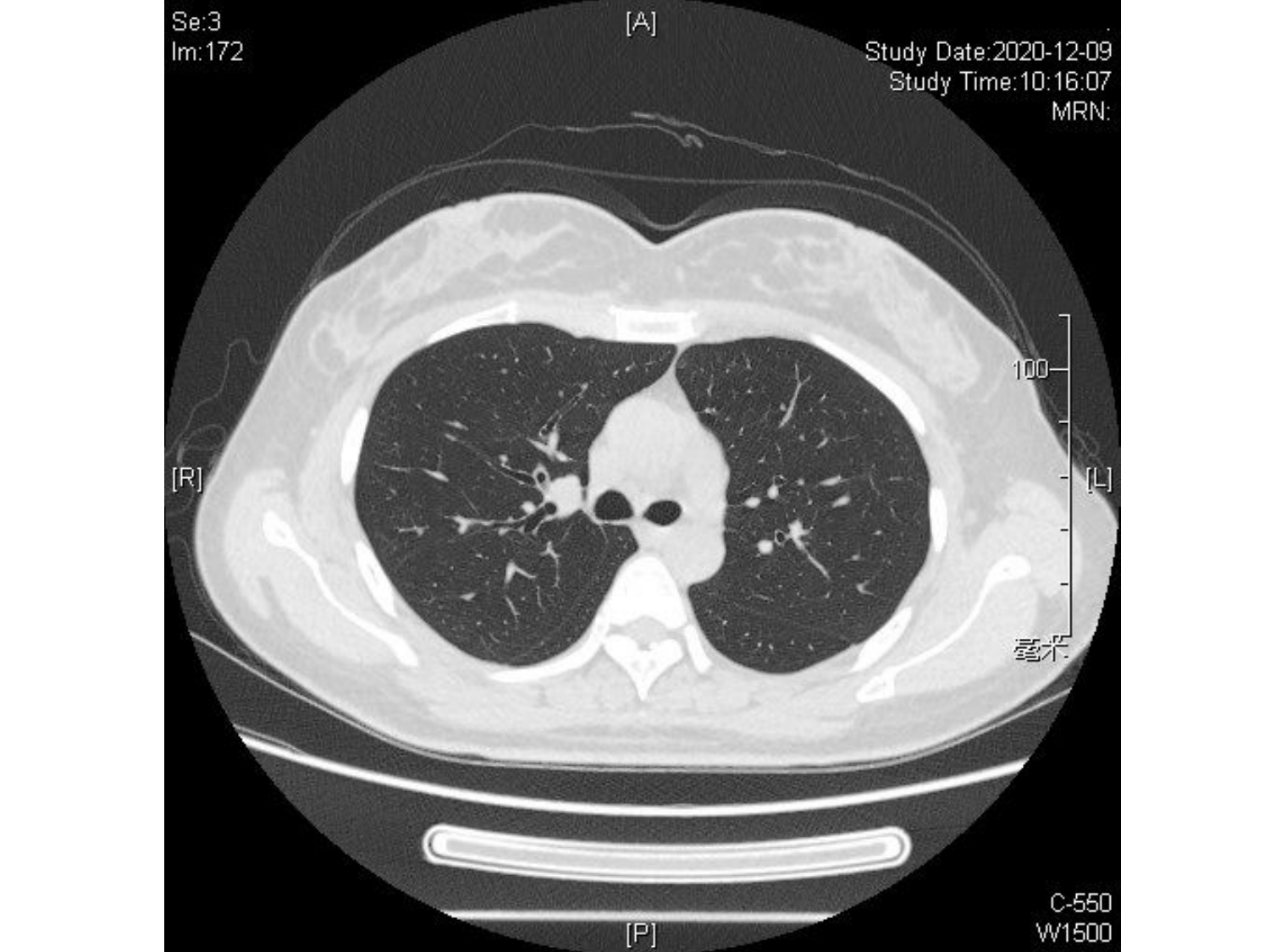

#

## Slide 38
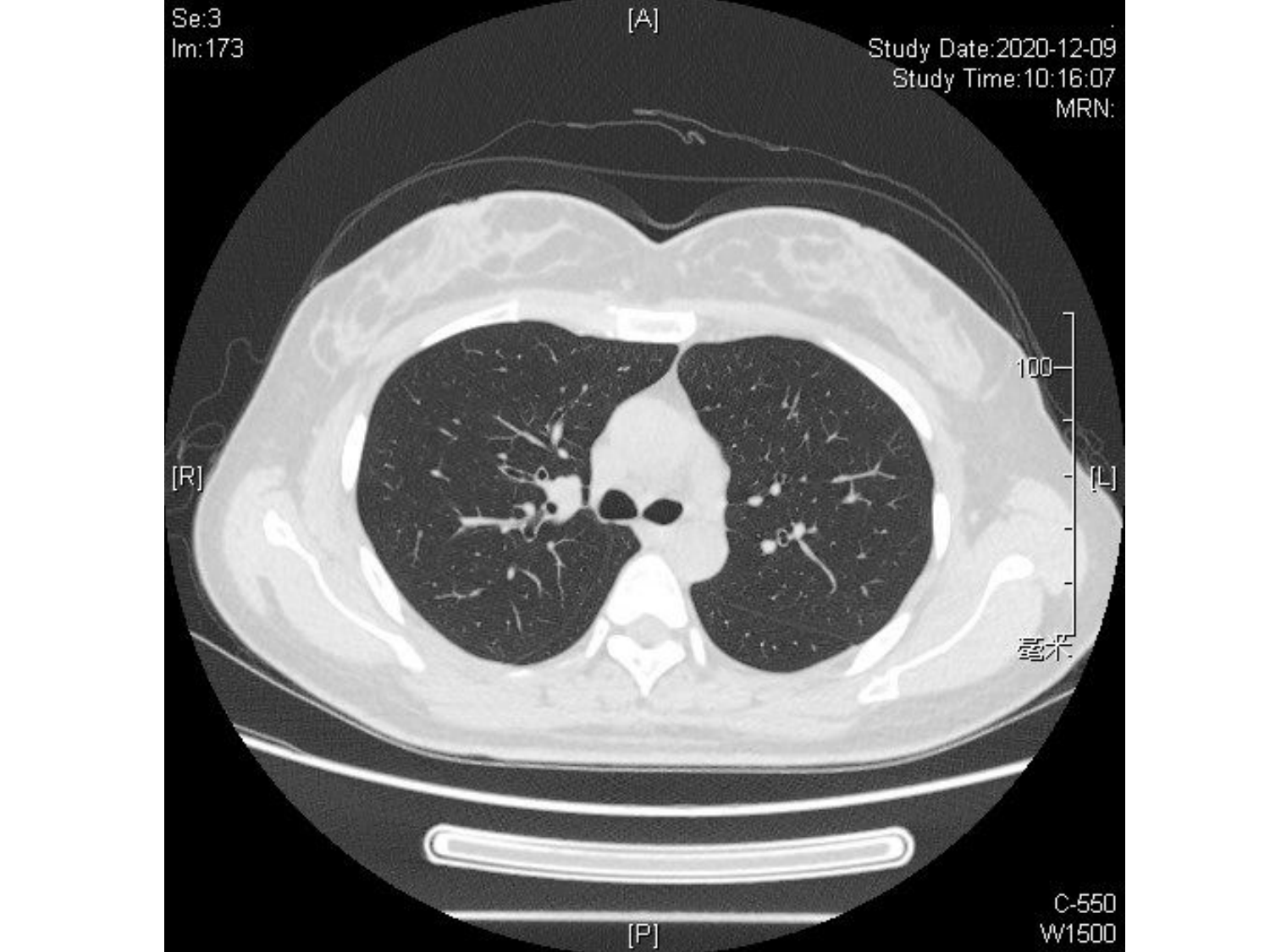

#

## Slide 39
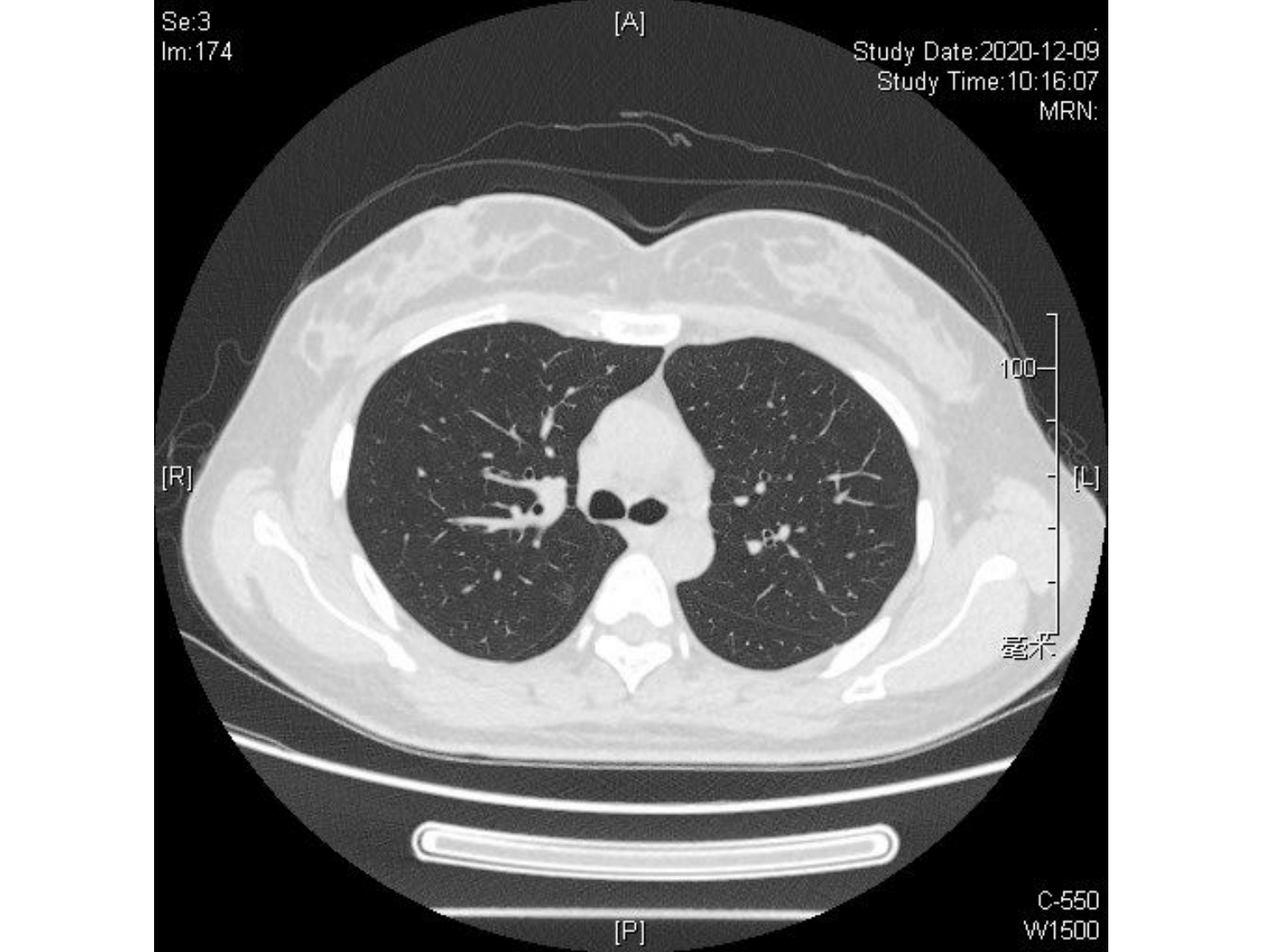

#

## Slide 40
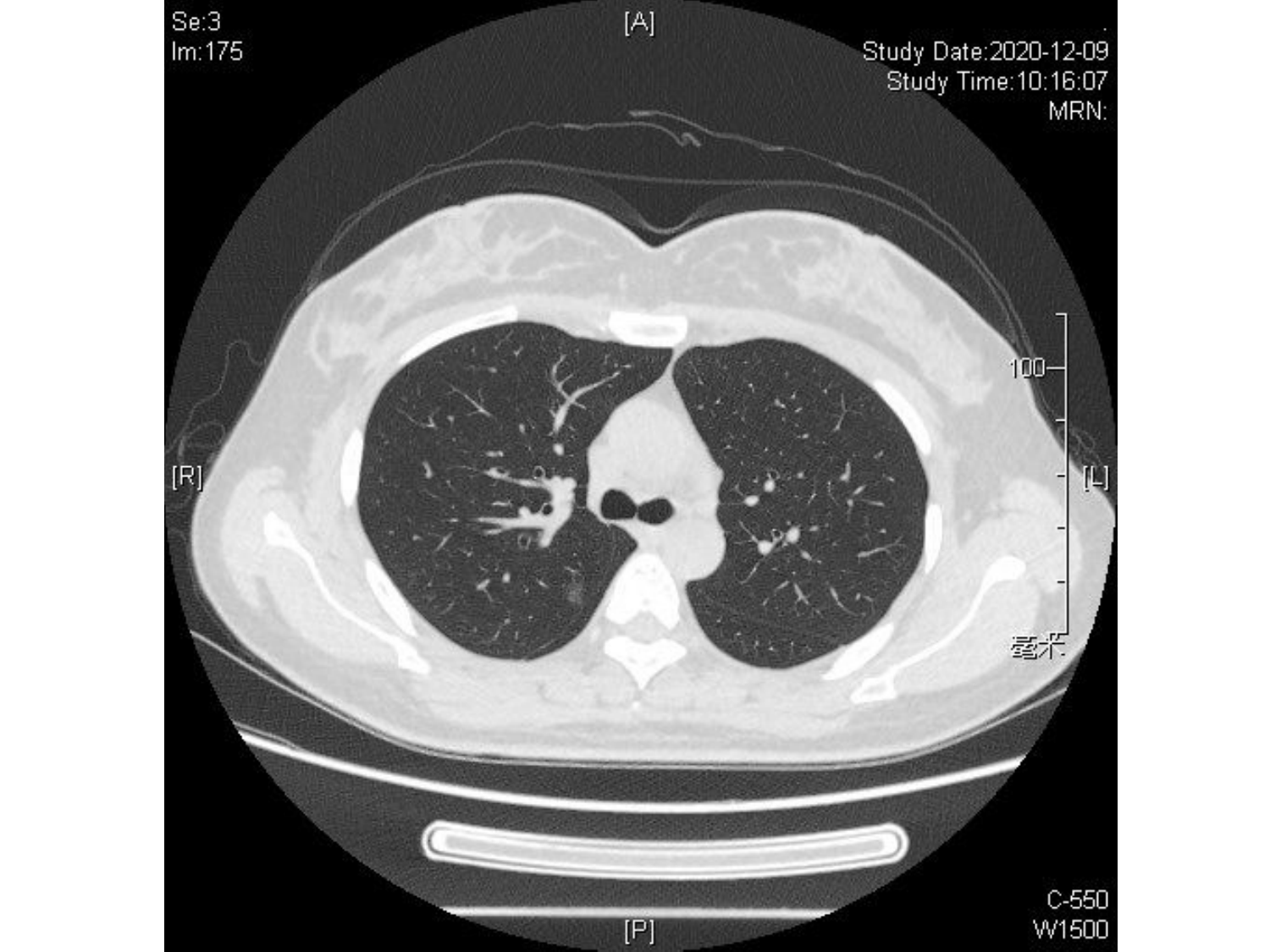

#

## Slide 41
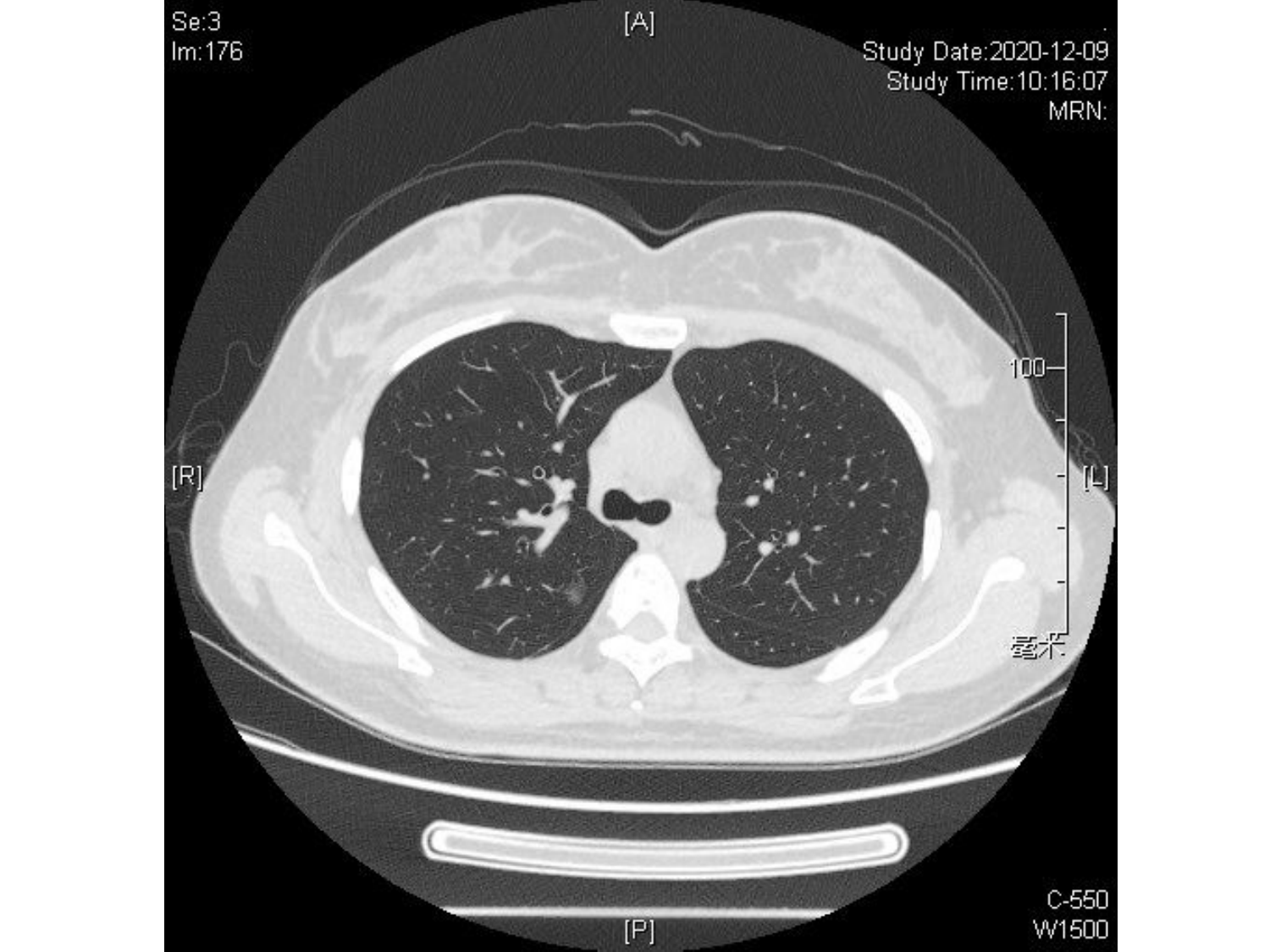

#

## Slide 42
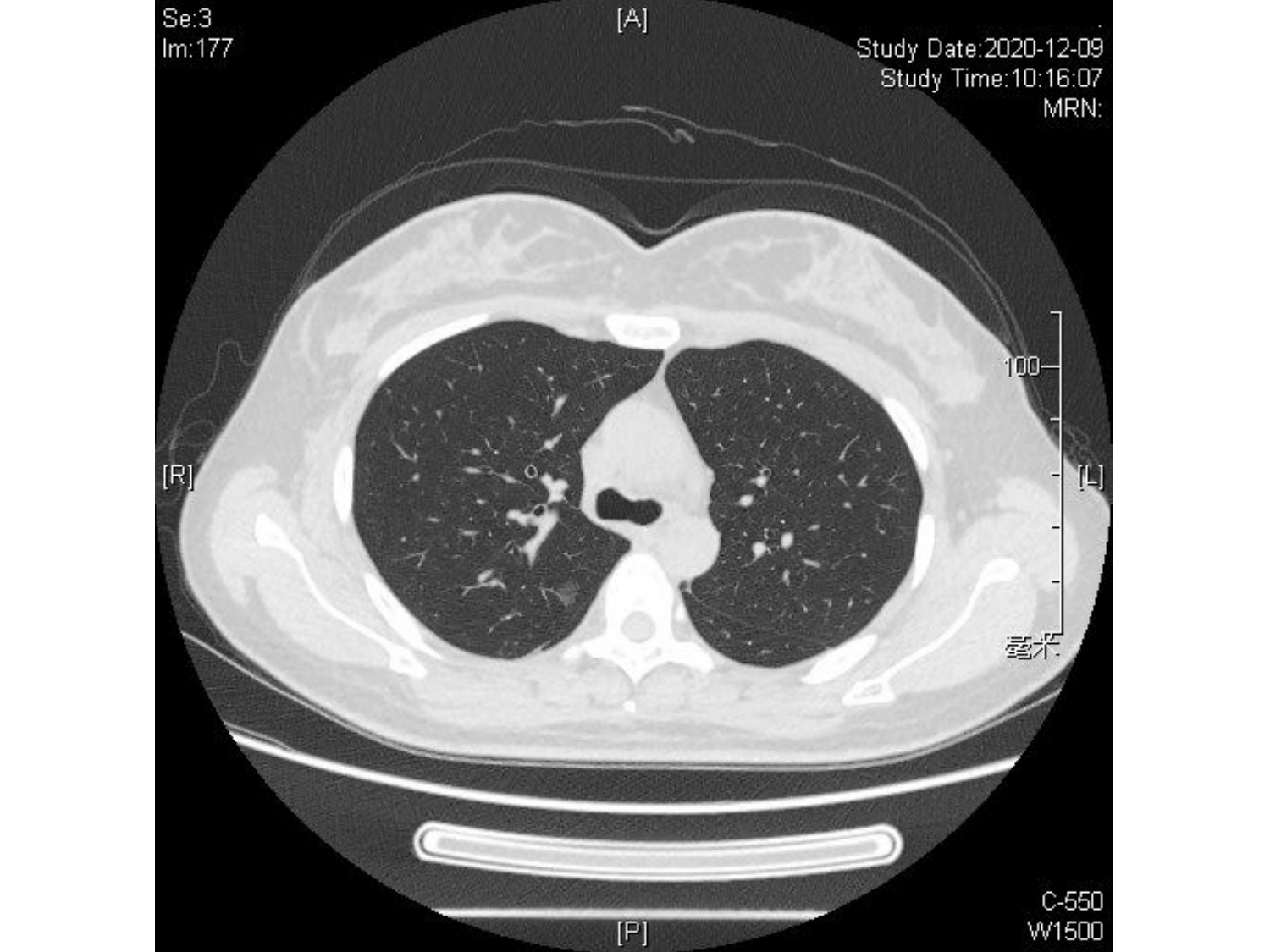

#

## Slide 43
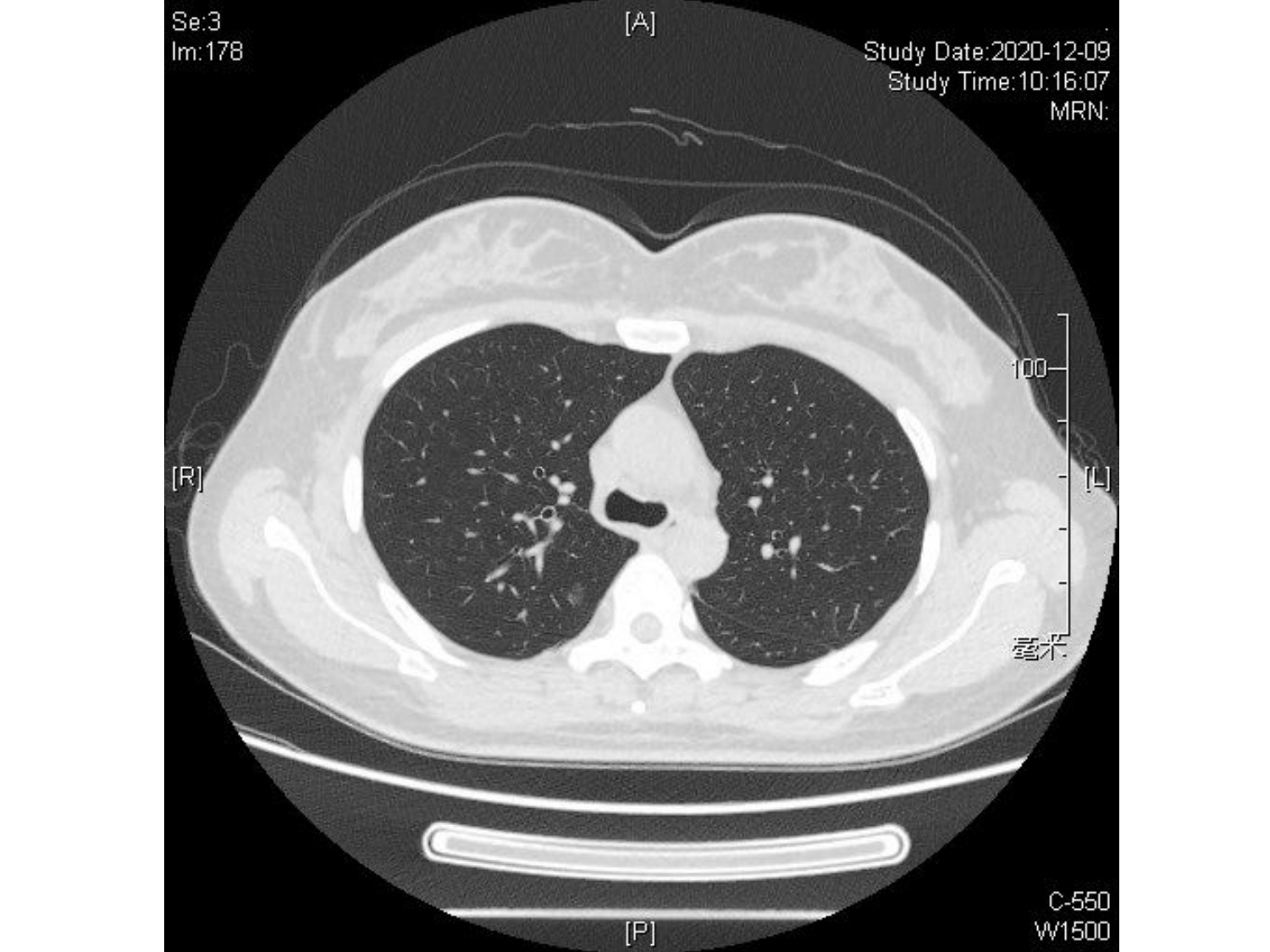

#

## Slide 44
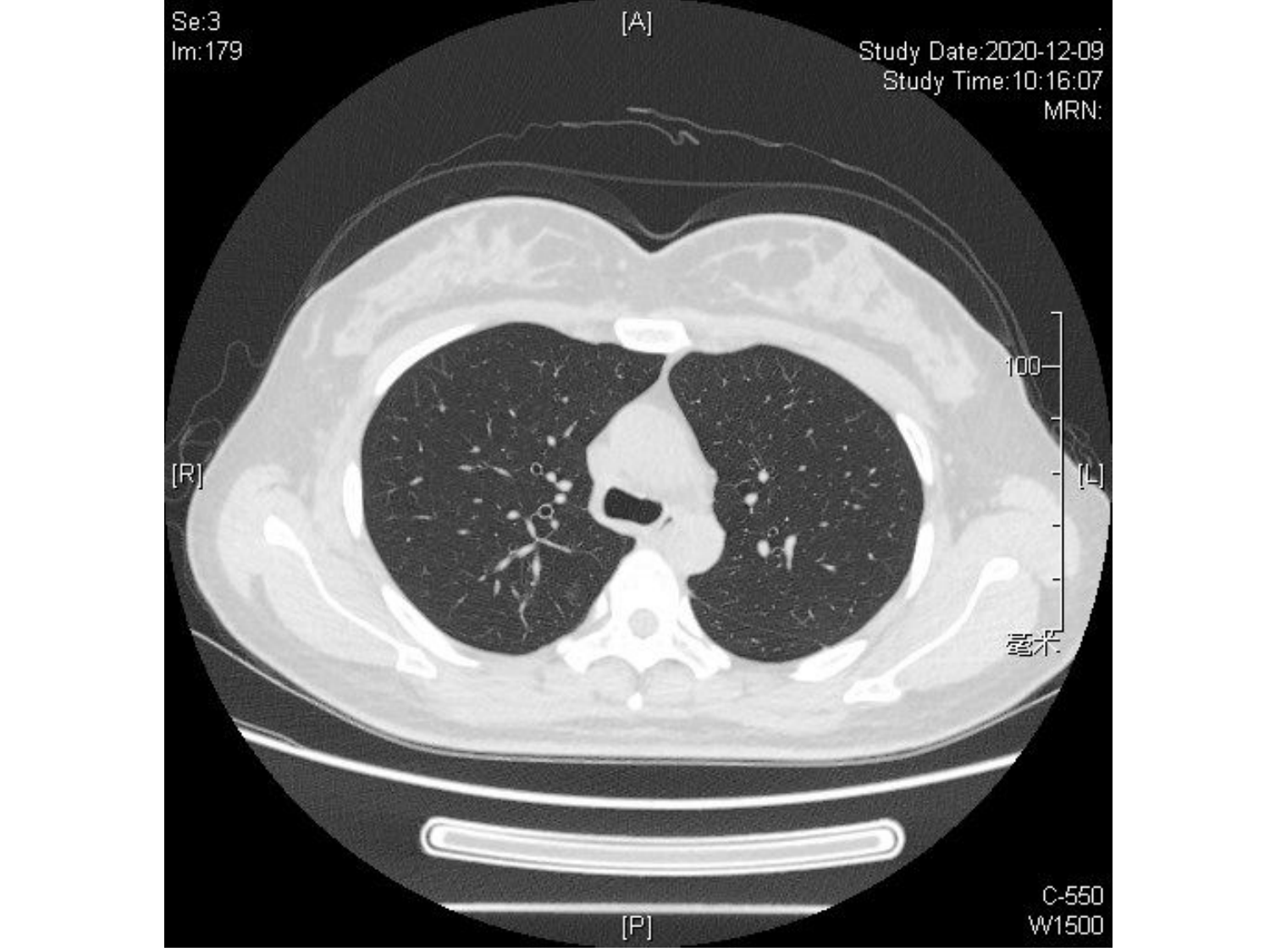

#

## Slide 45
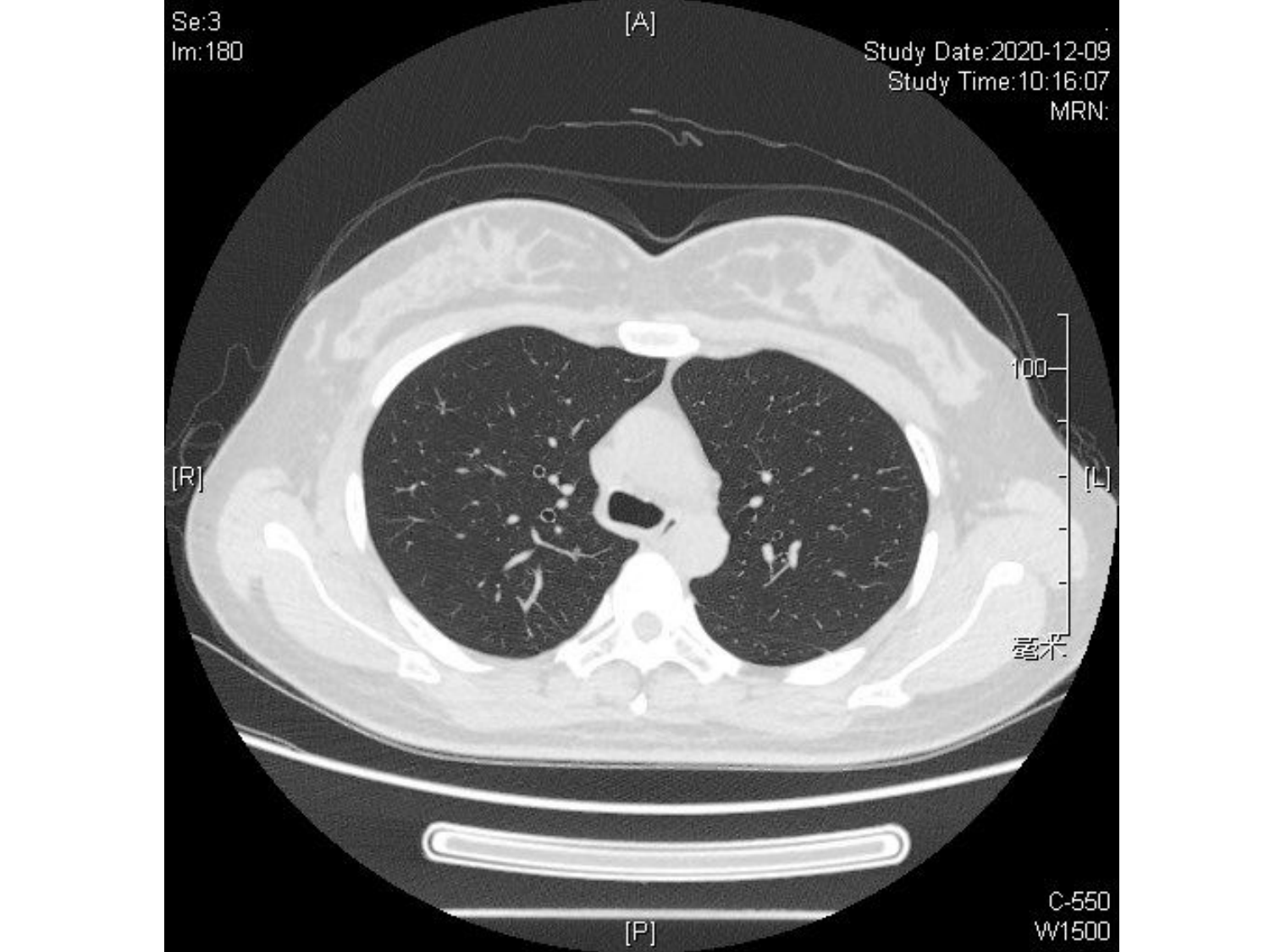

#

## Slide 46
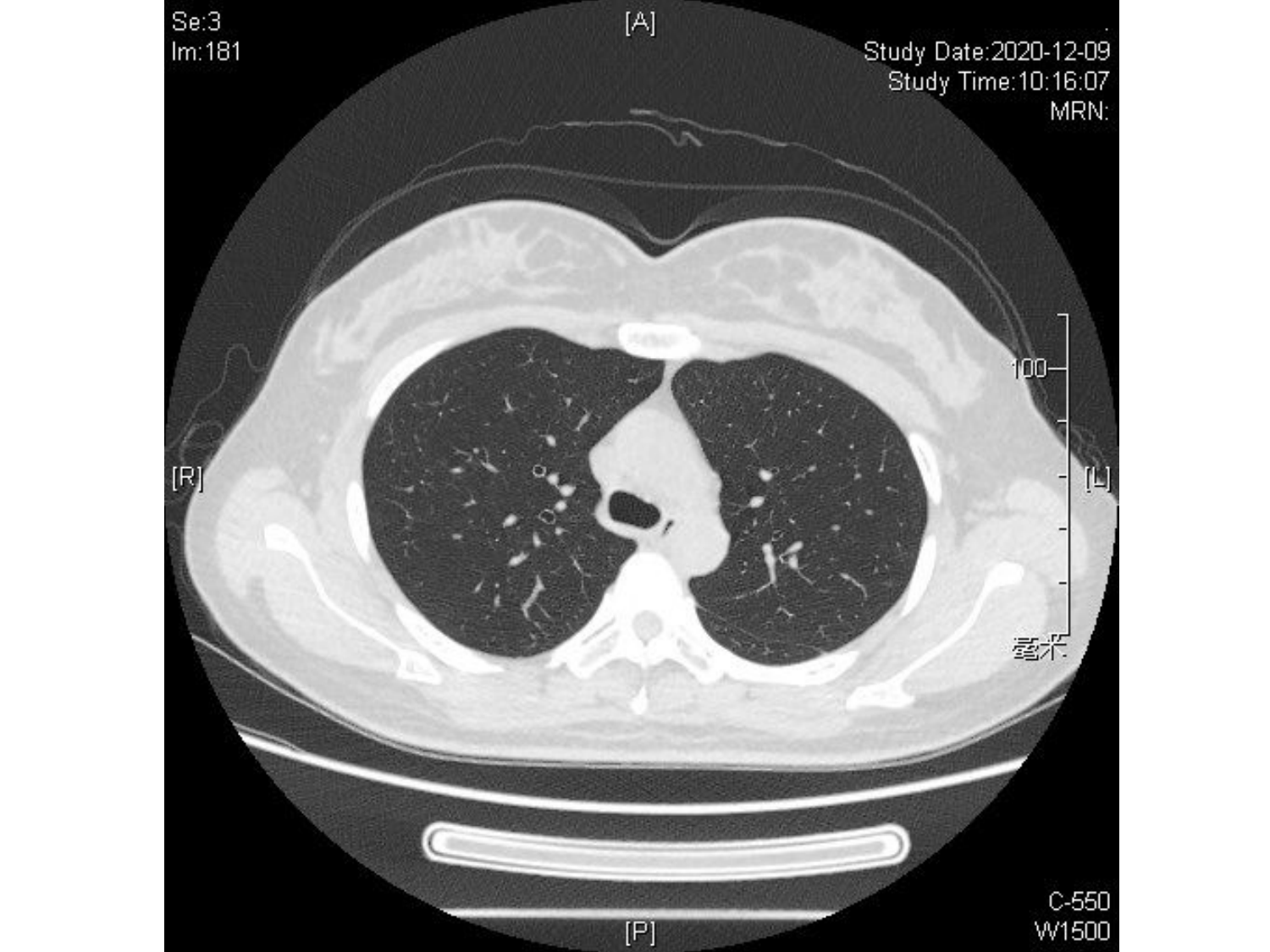

#

## Slide 47
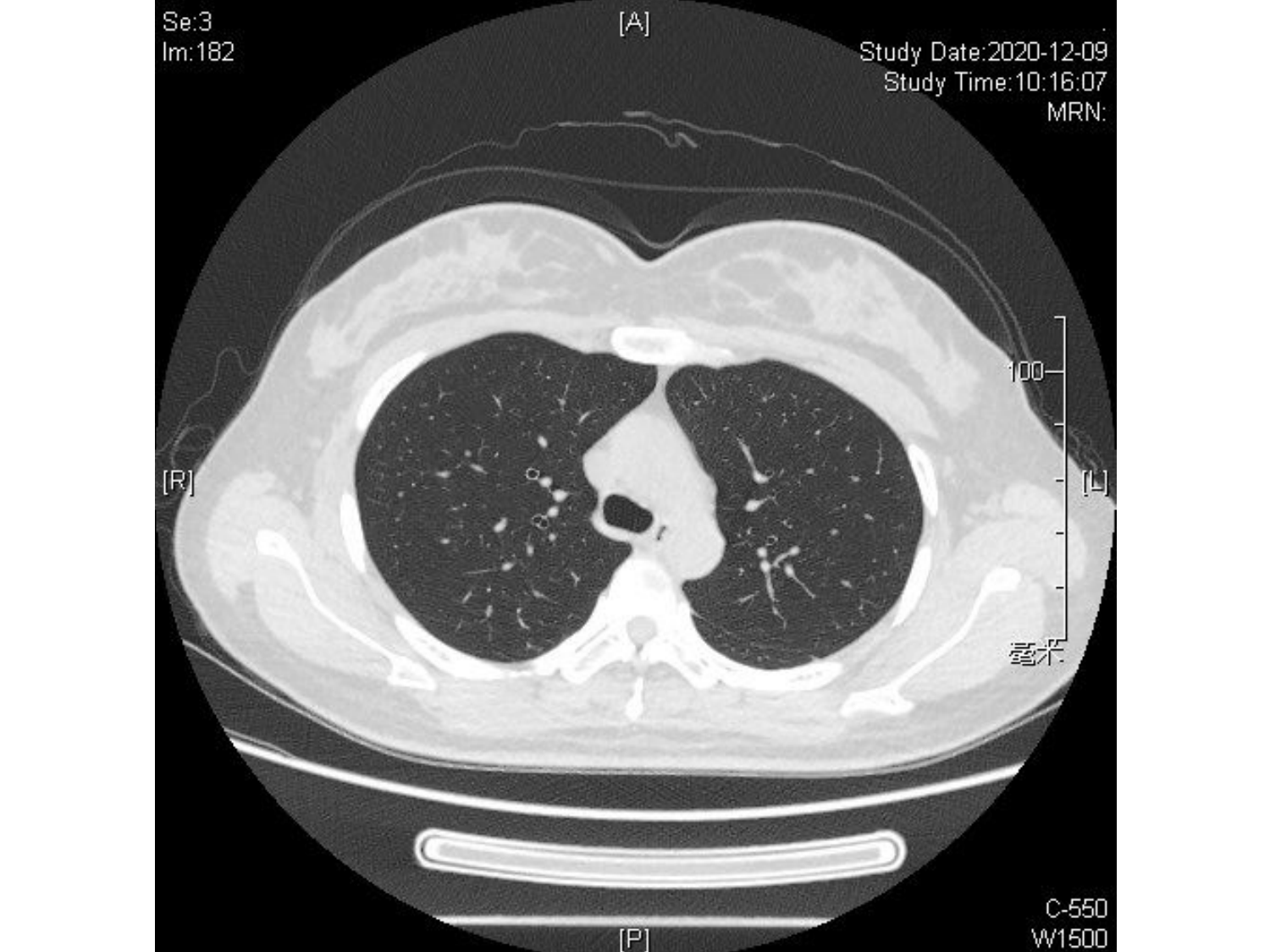

#

## Slide 48
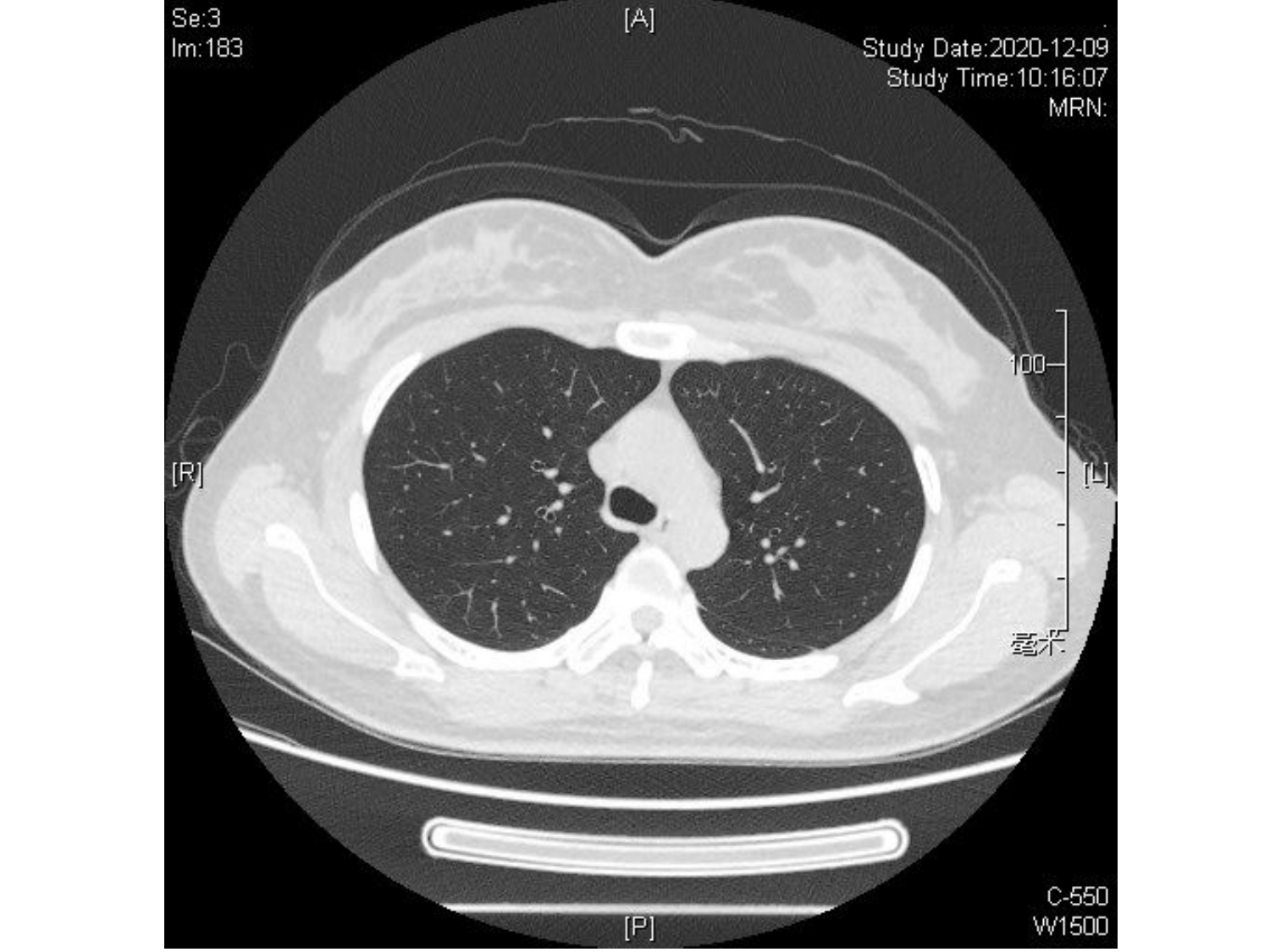

#

## Slide 49
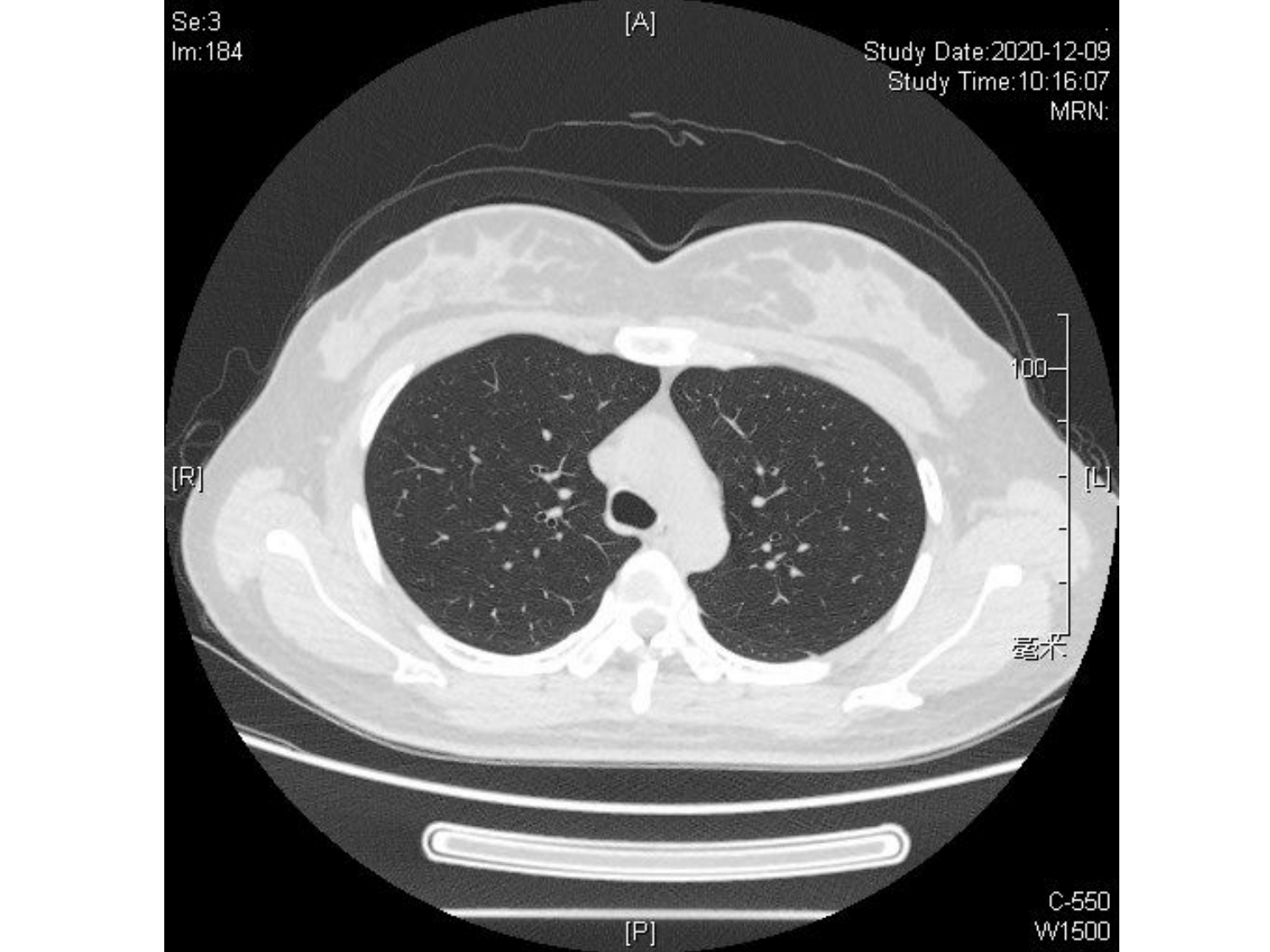

#

## Slide 50
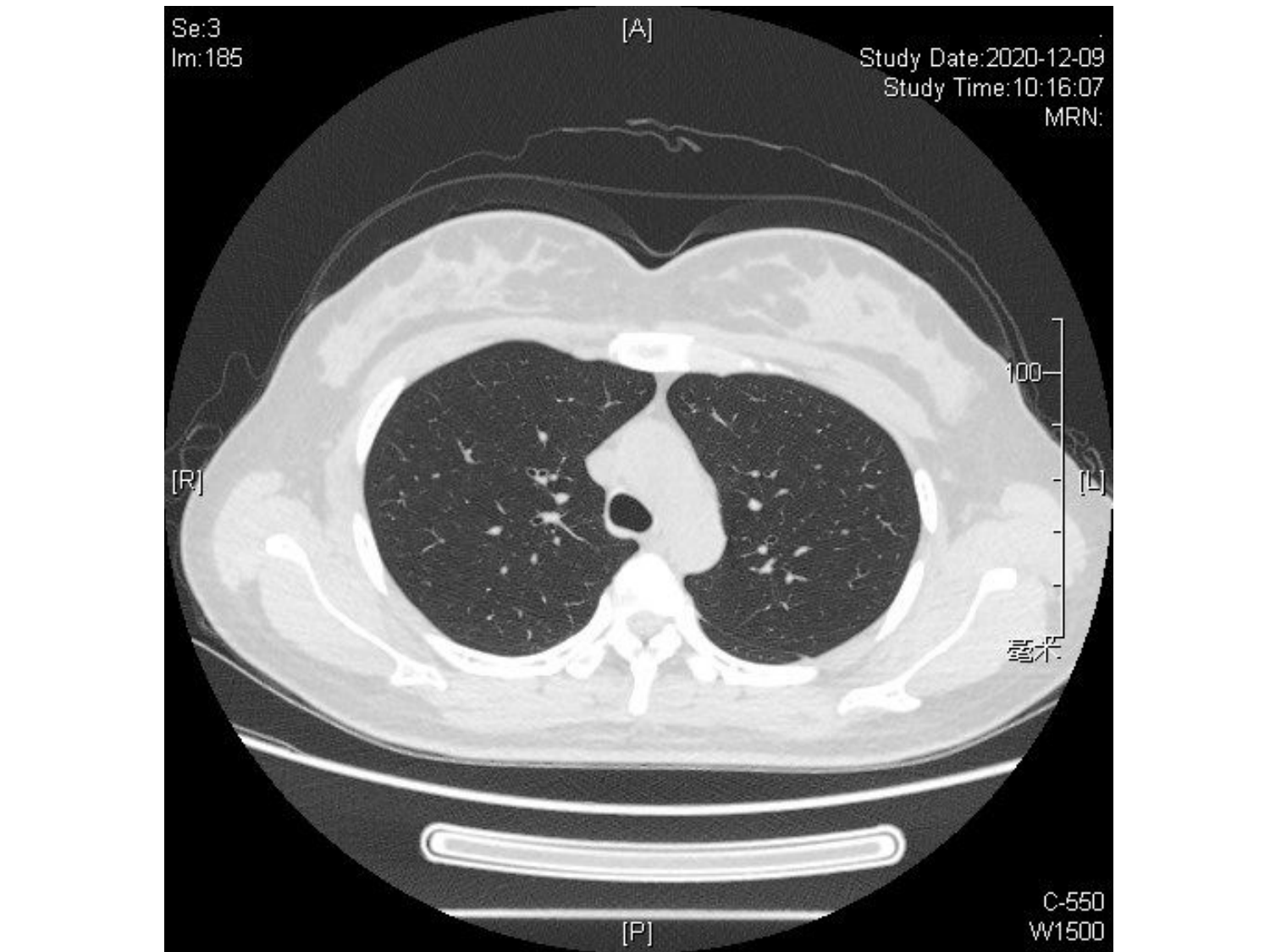

#

## Slide 51
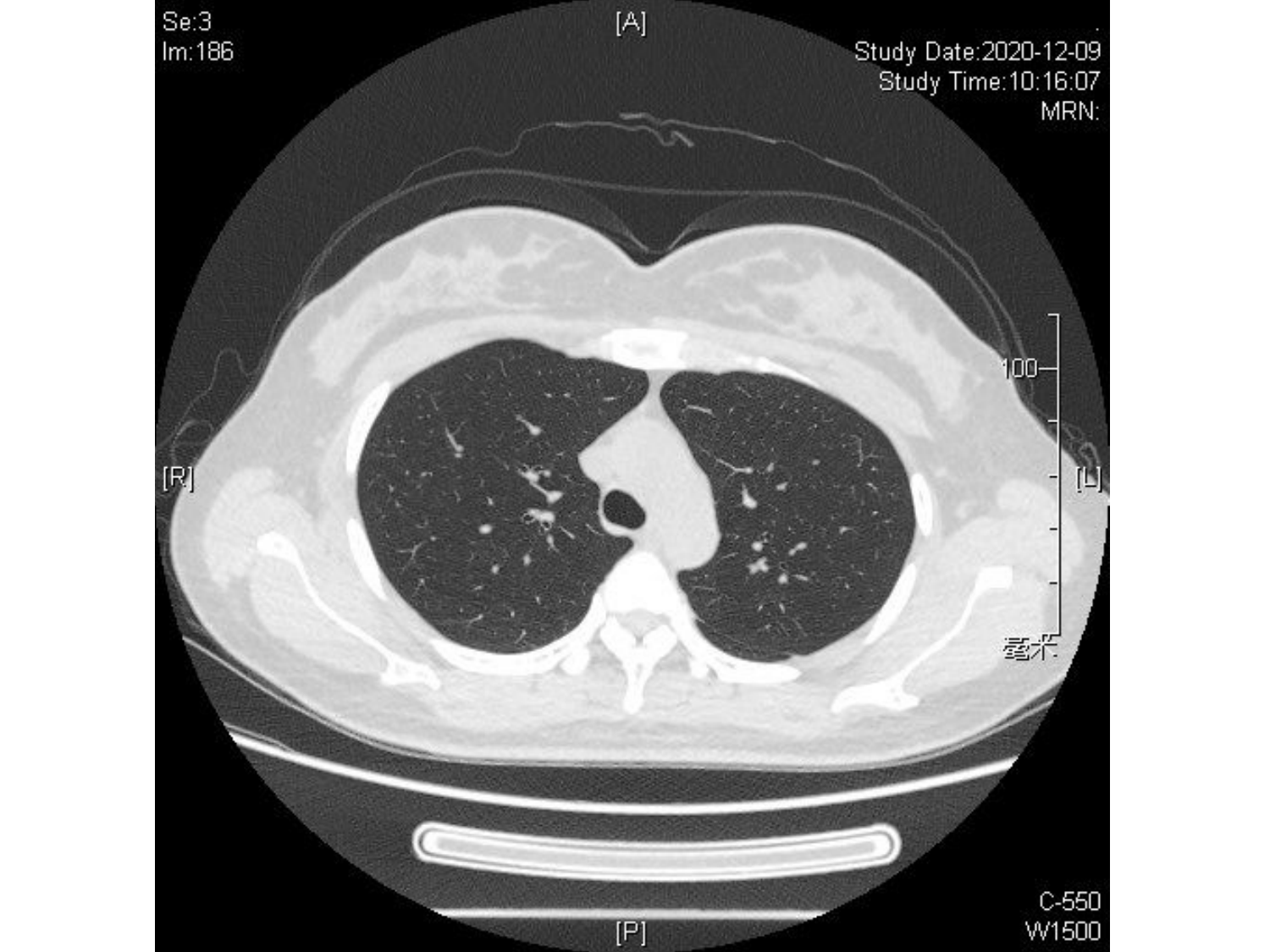

#

## Slide 52
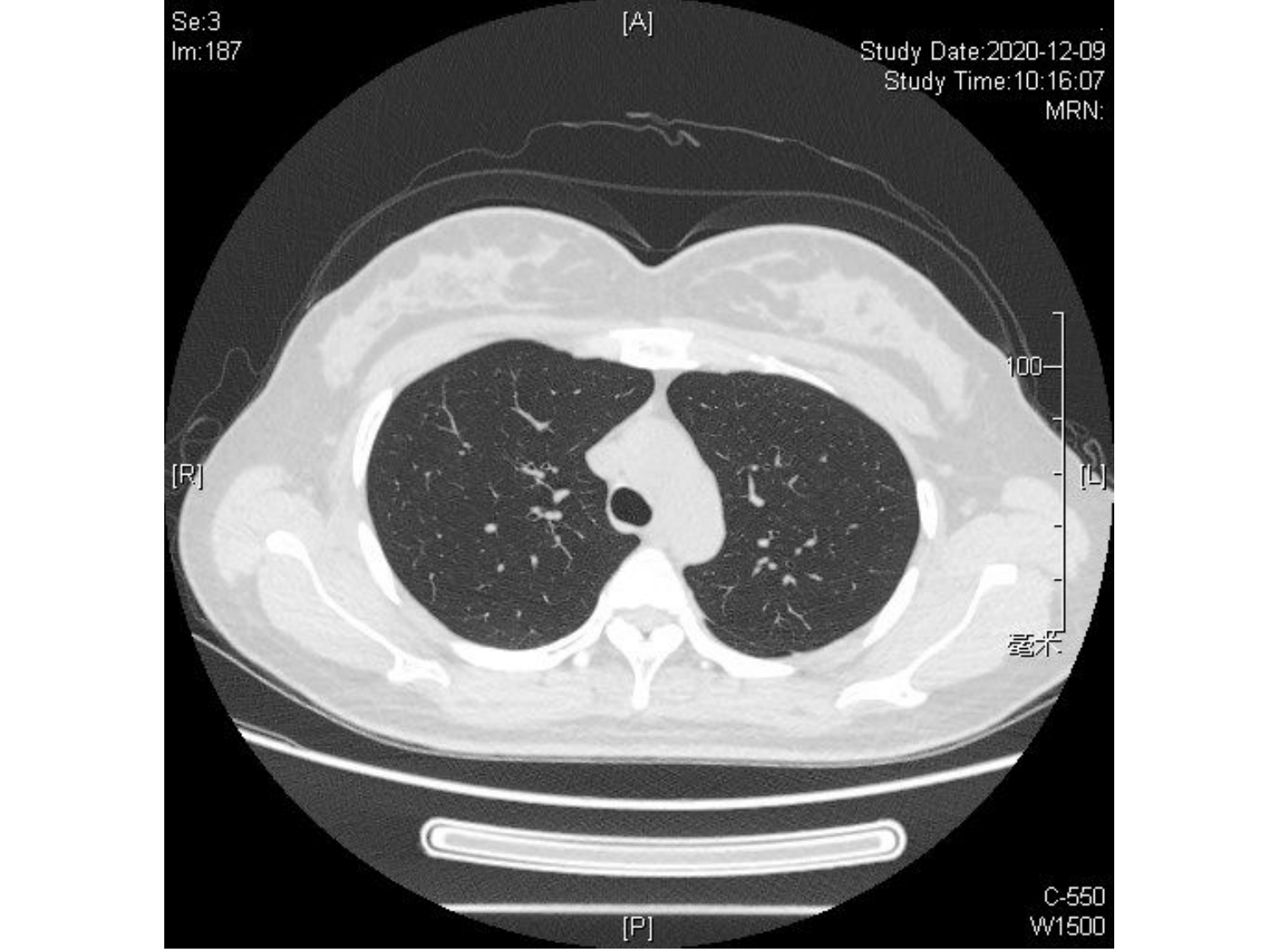

#

## Slide 53
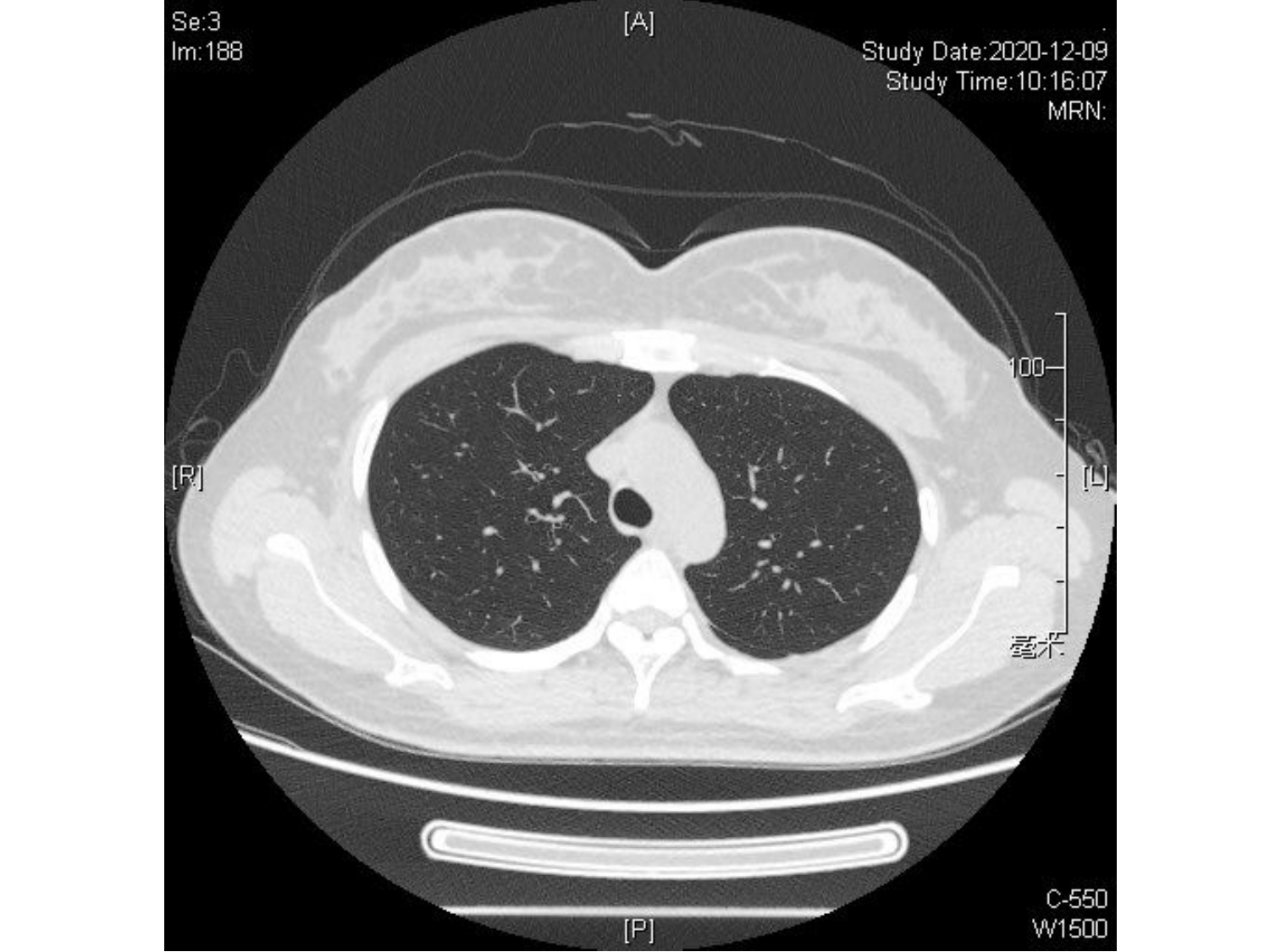

#

## Slide 54
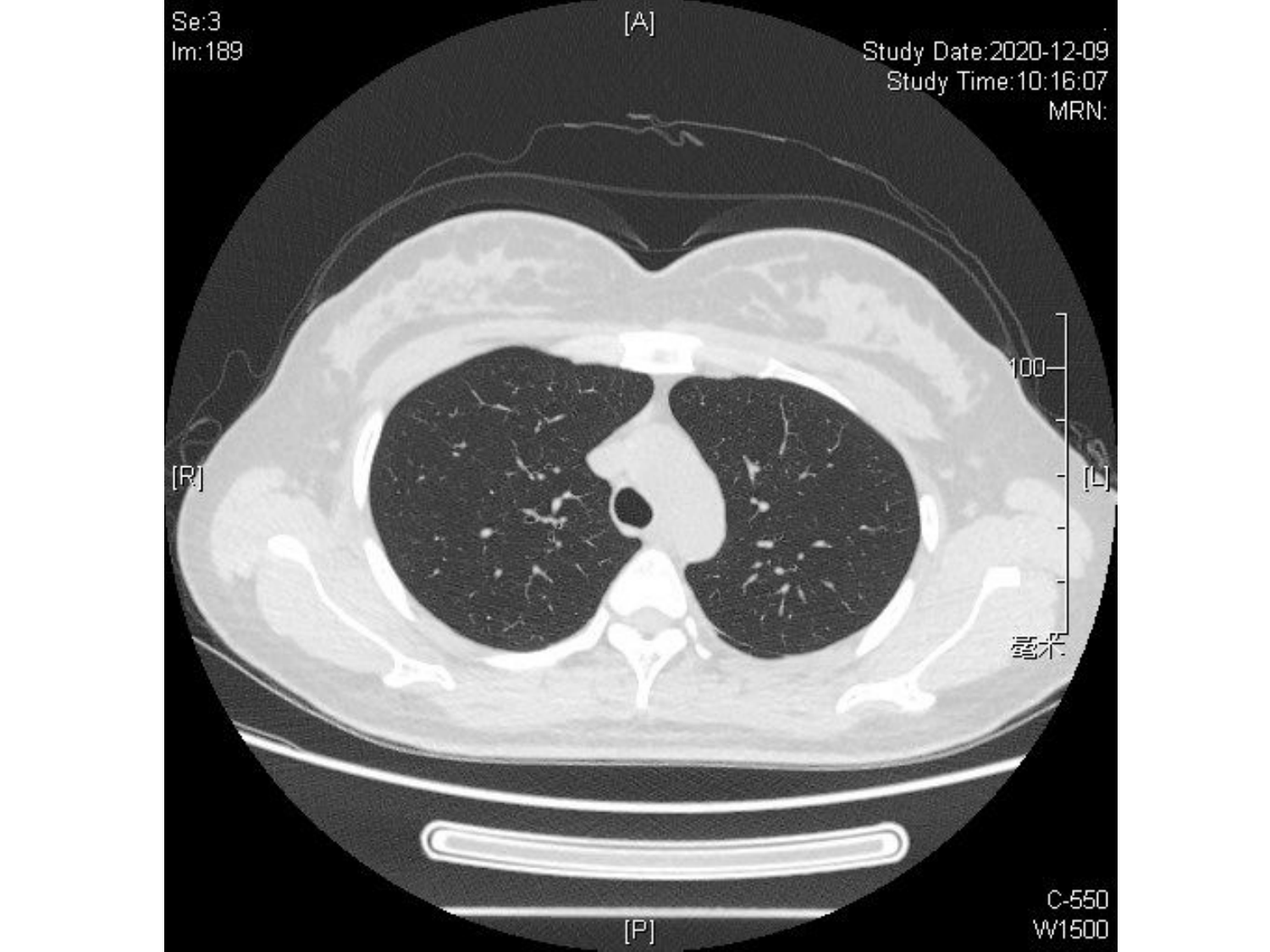

#

## Slide 55
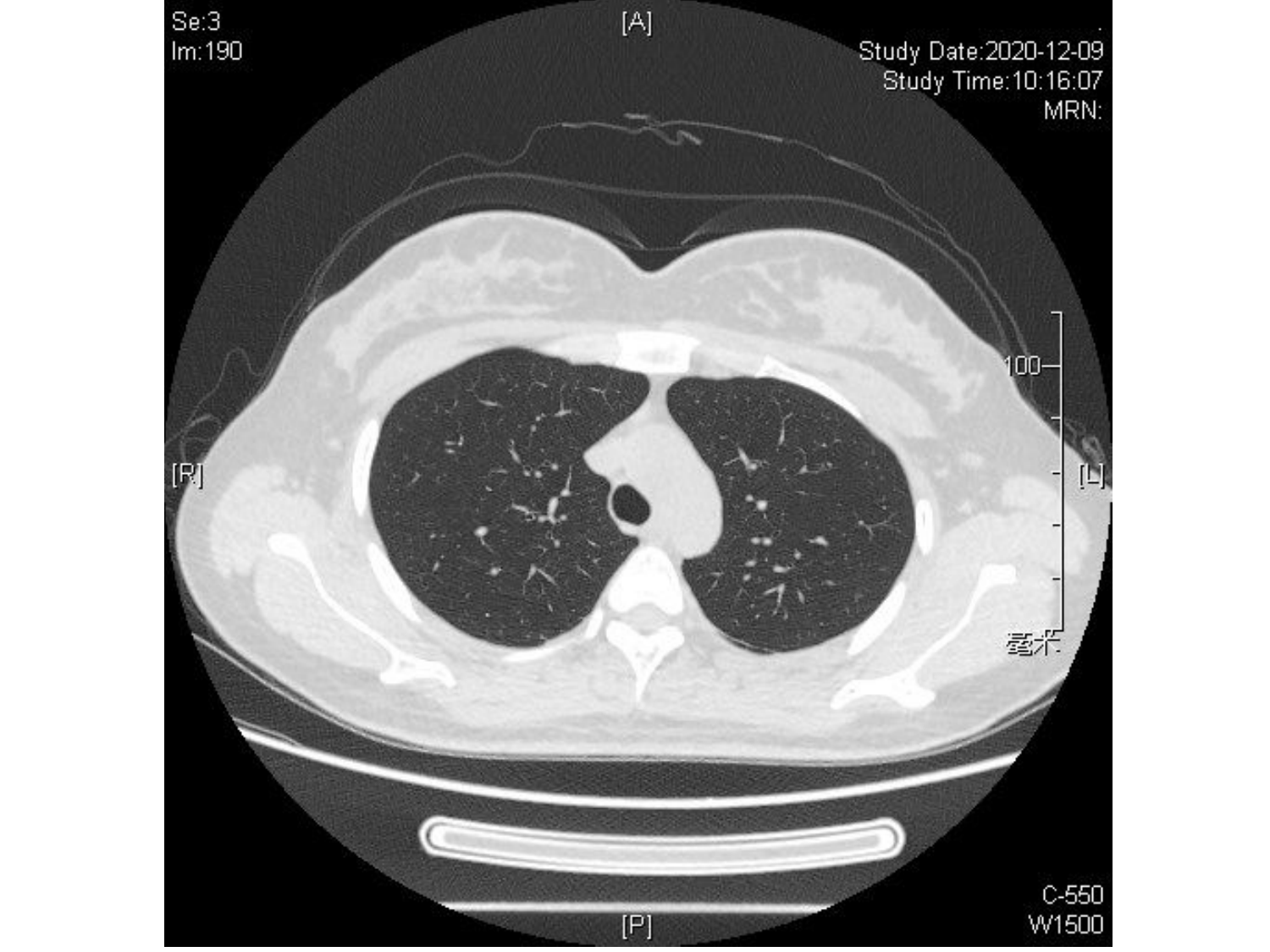

#

## Slide 56
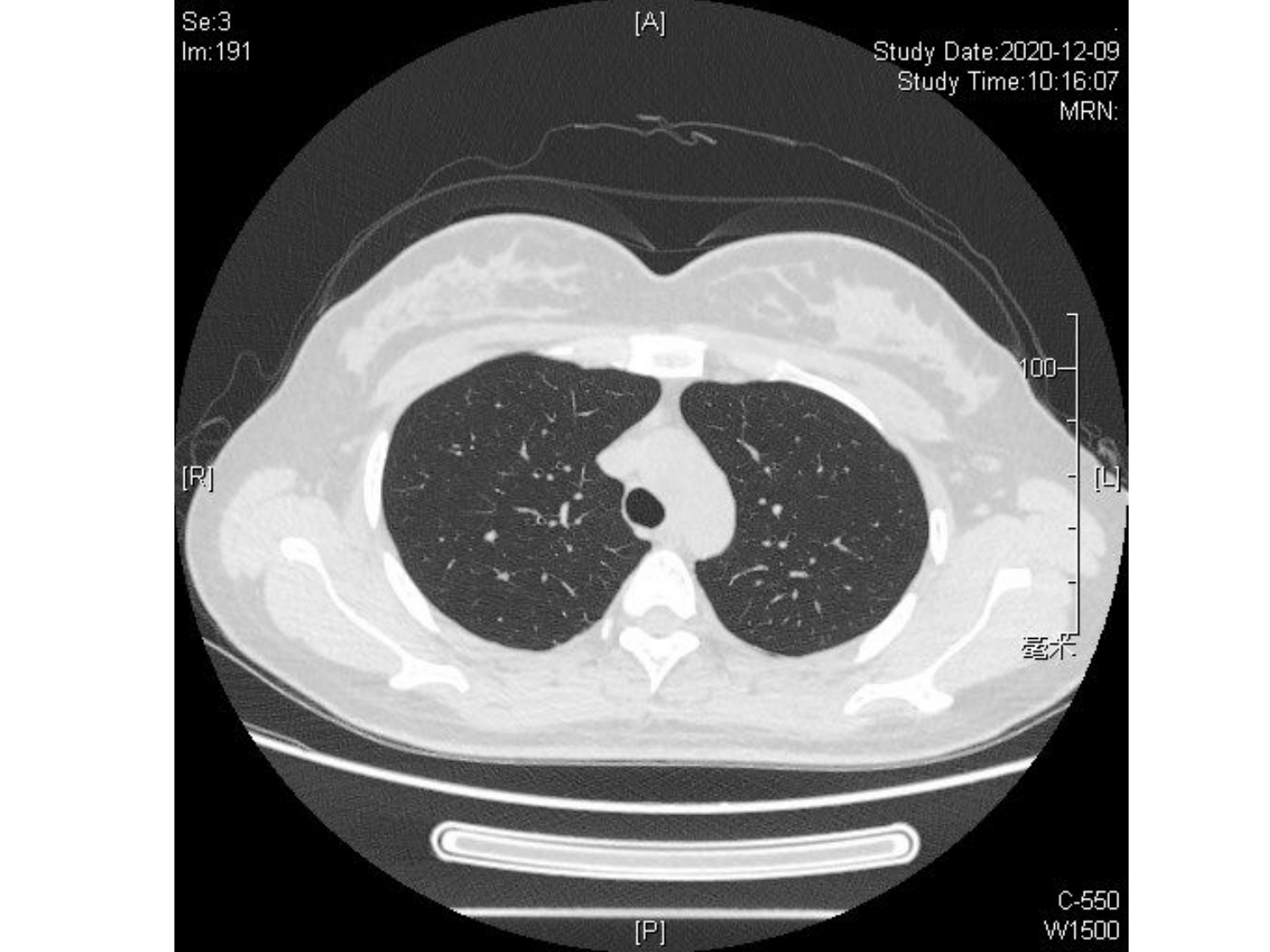

#

## Slide 57
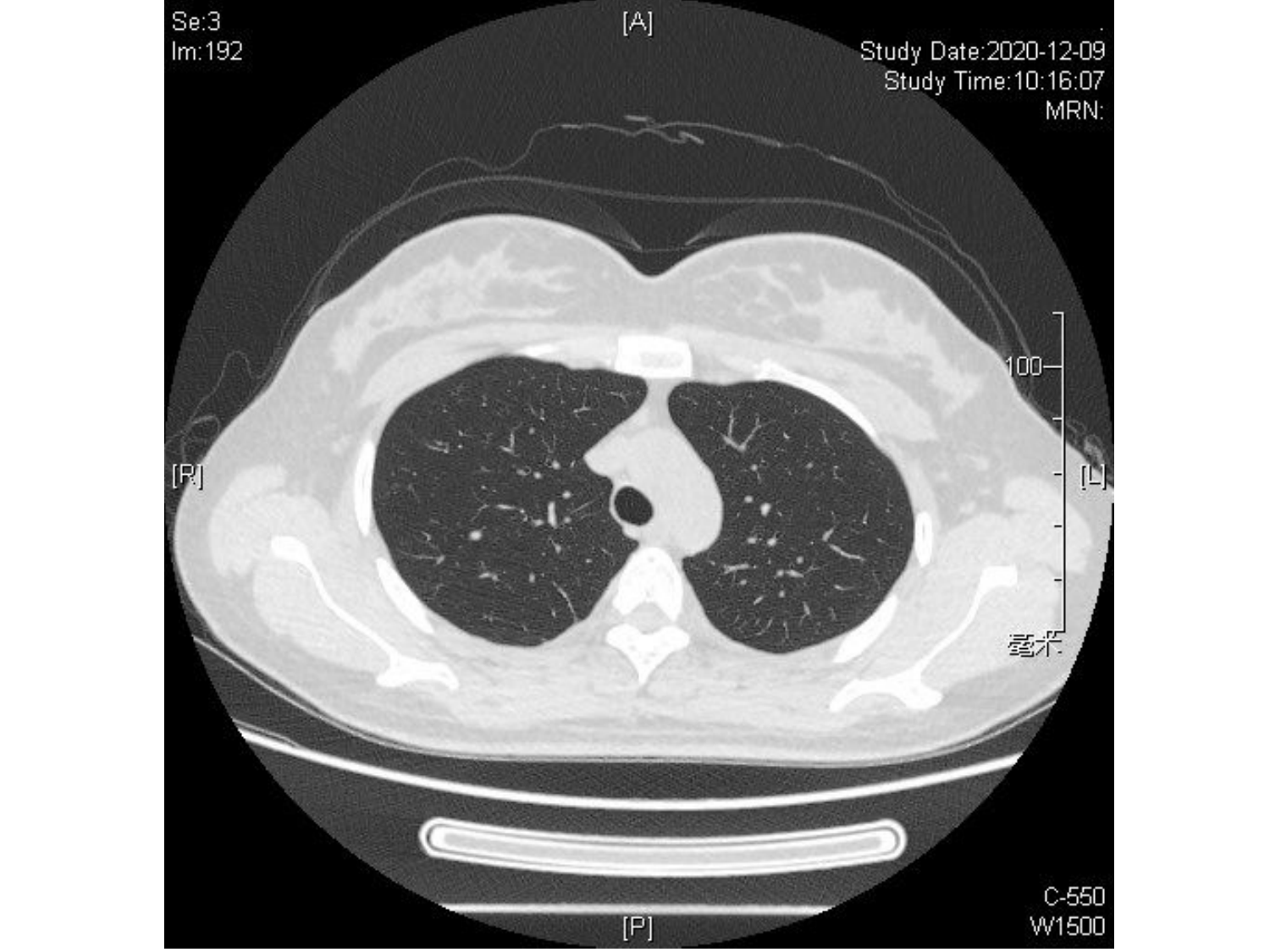

#

## Slide 58
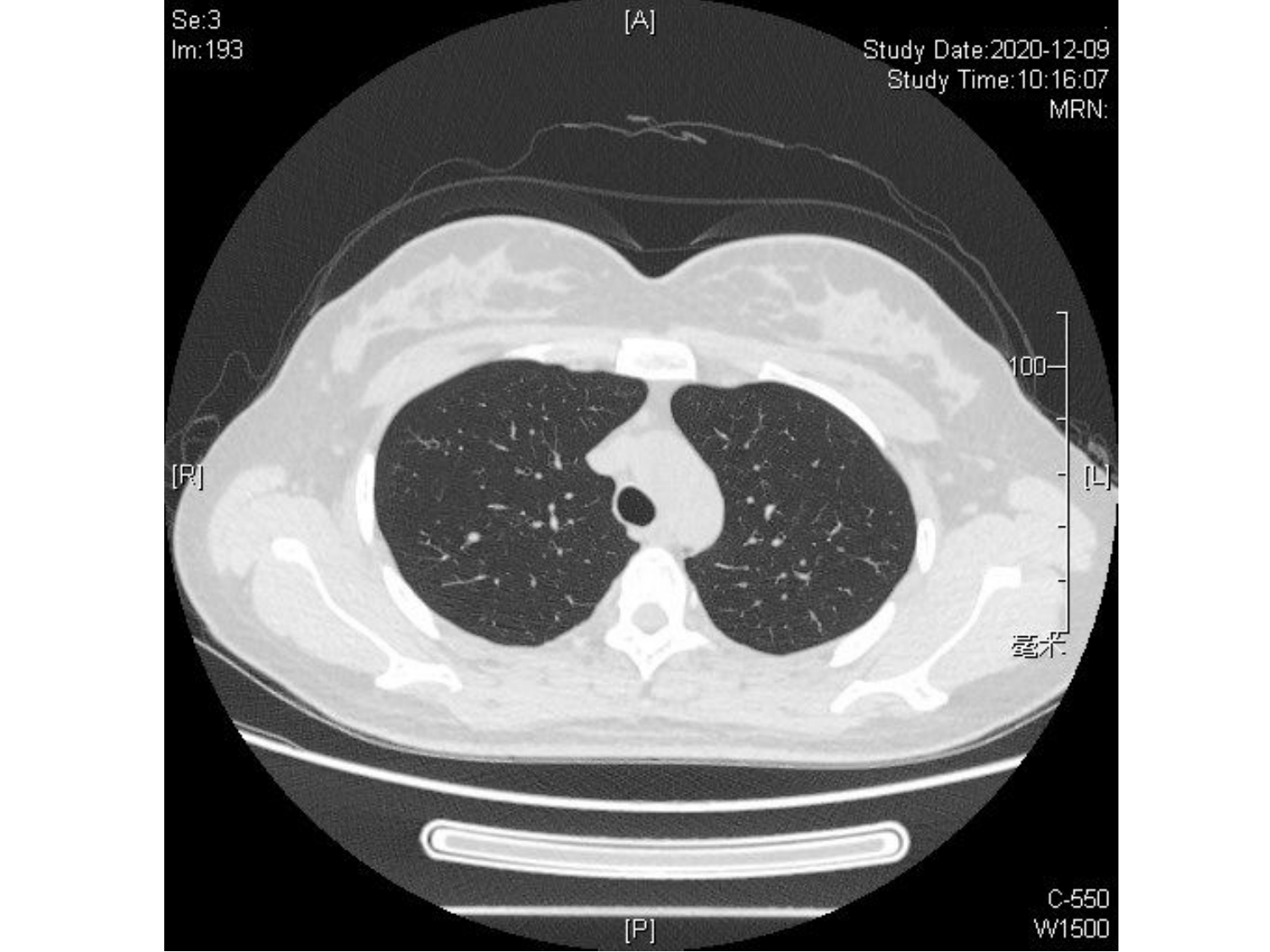

#

## Slide 59
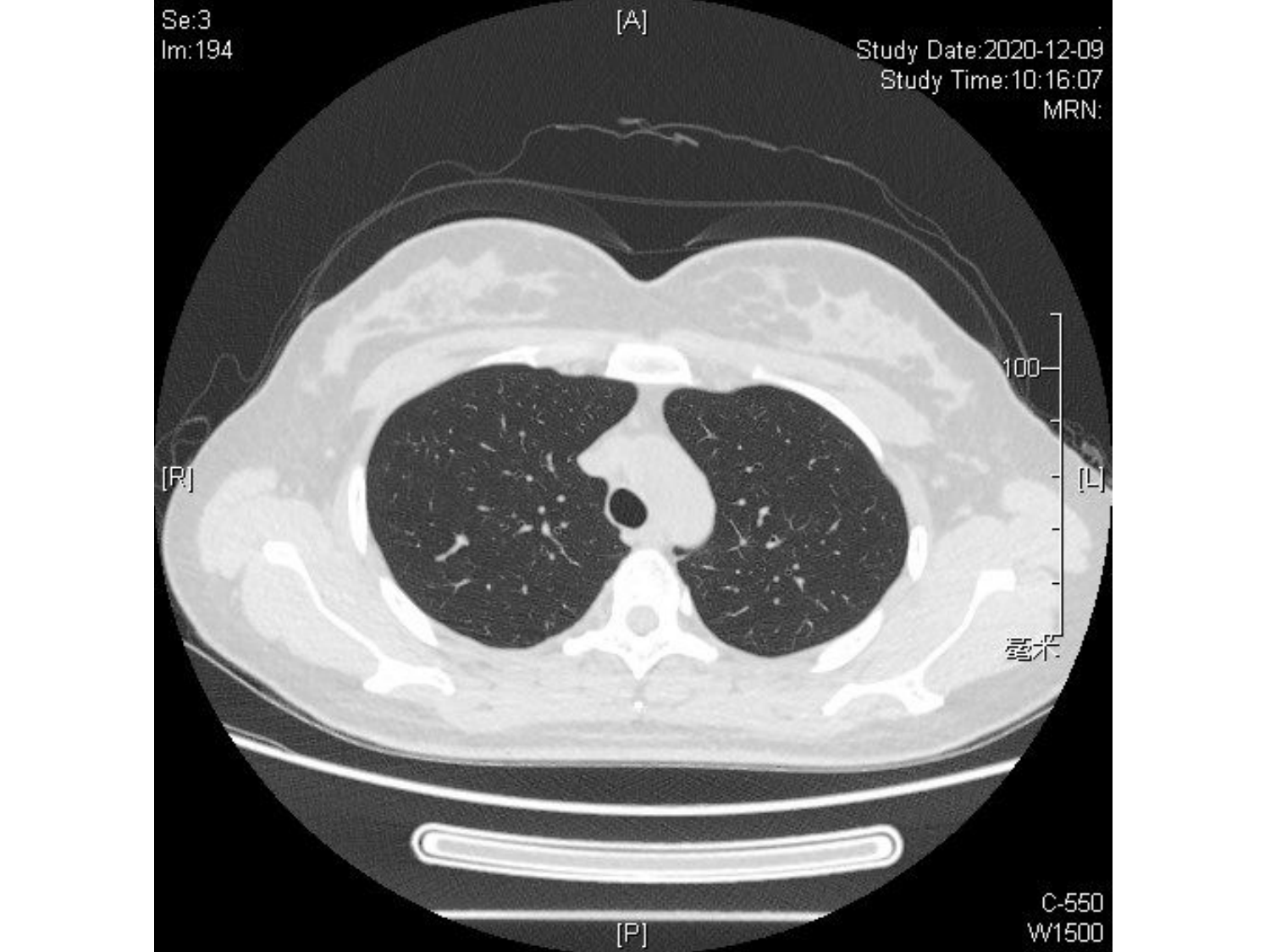

#

## Slide 60
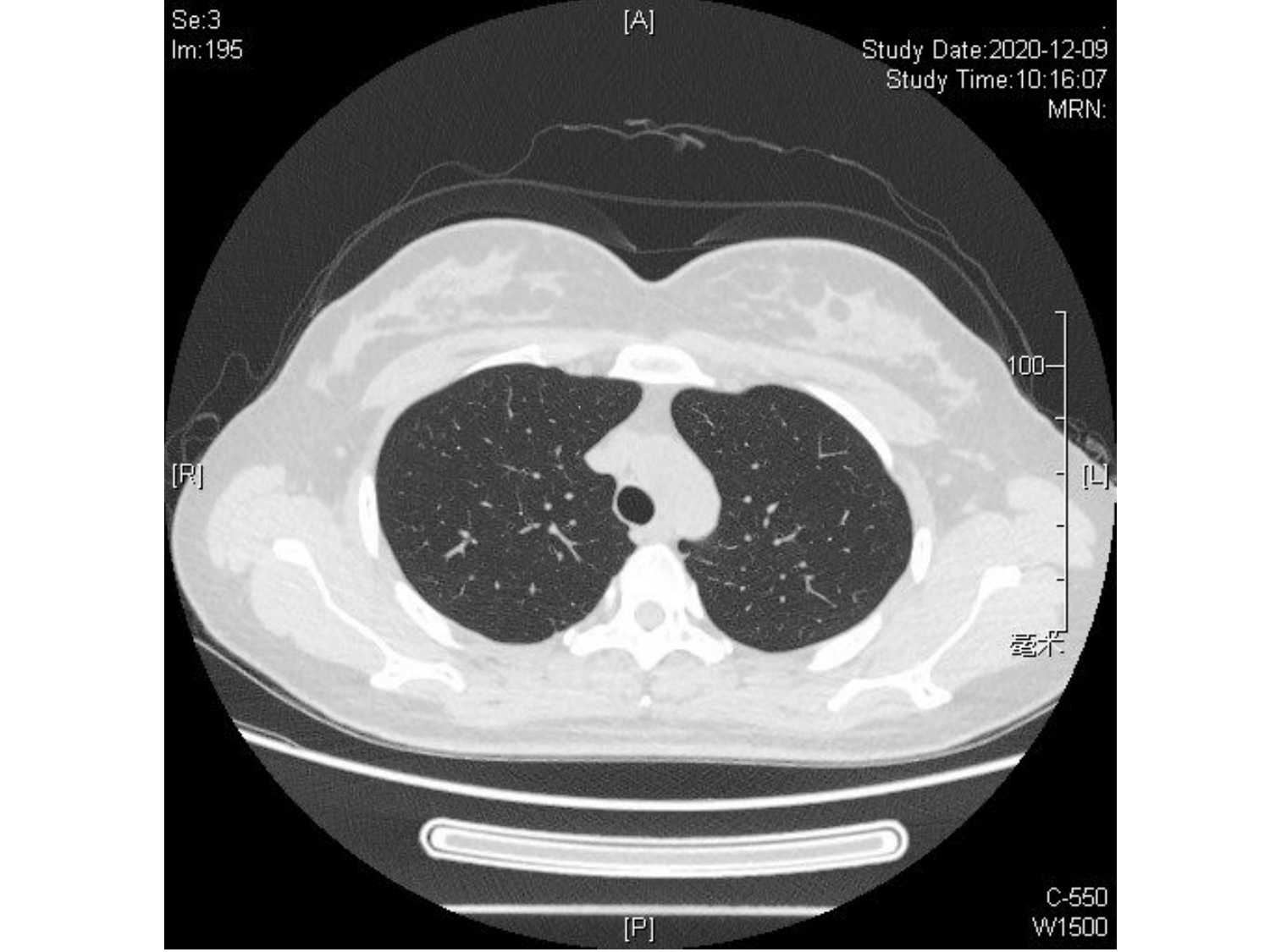

#

## Slide 61
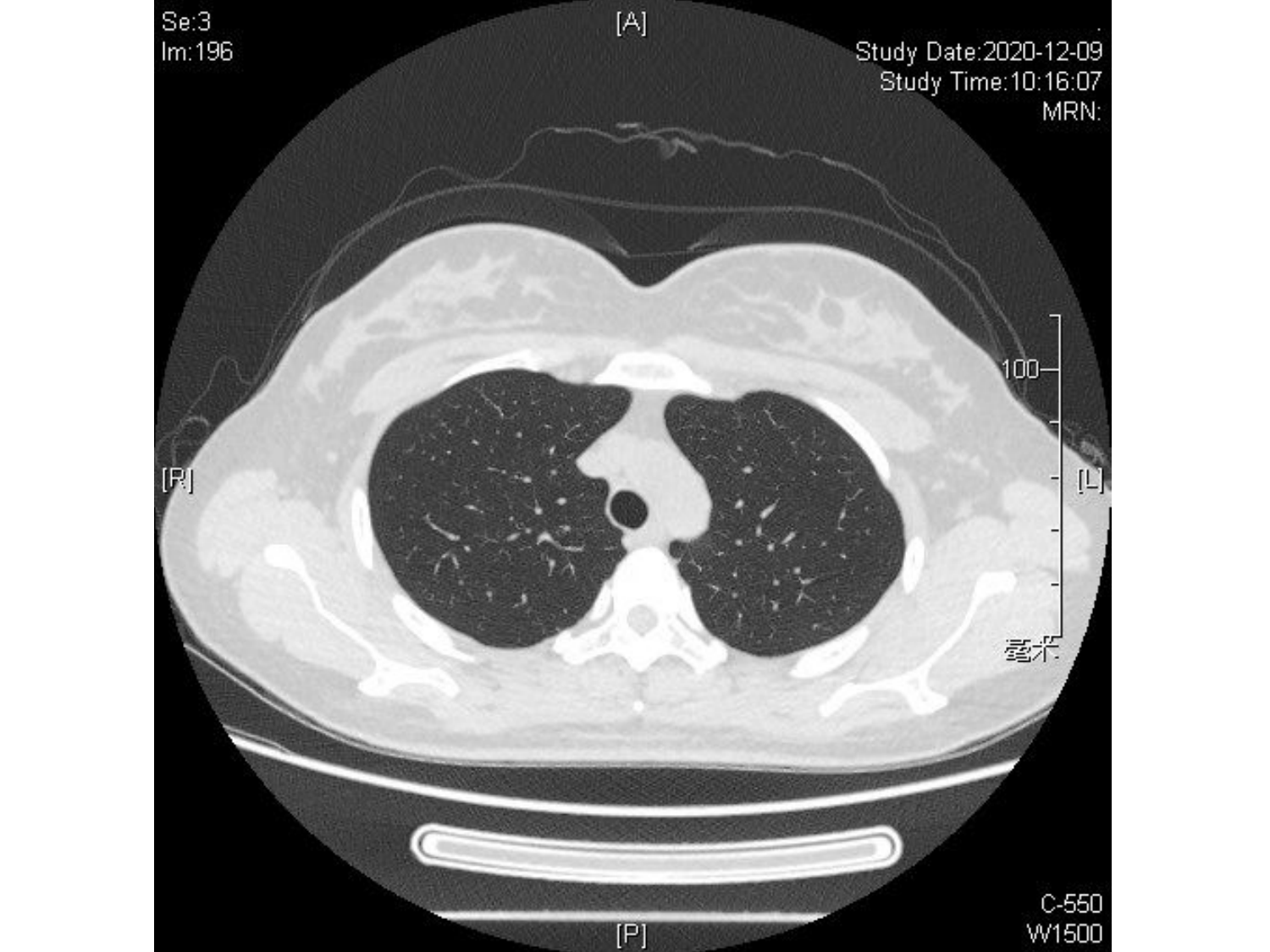

#

## Slide 62
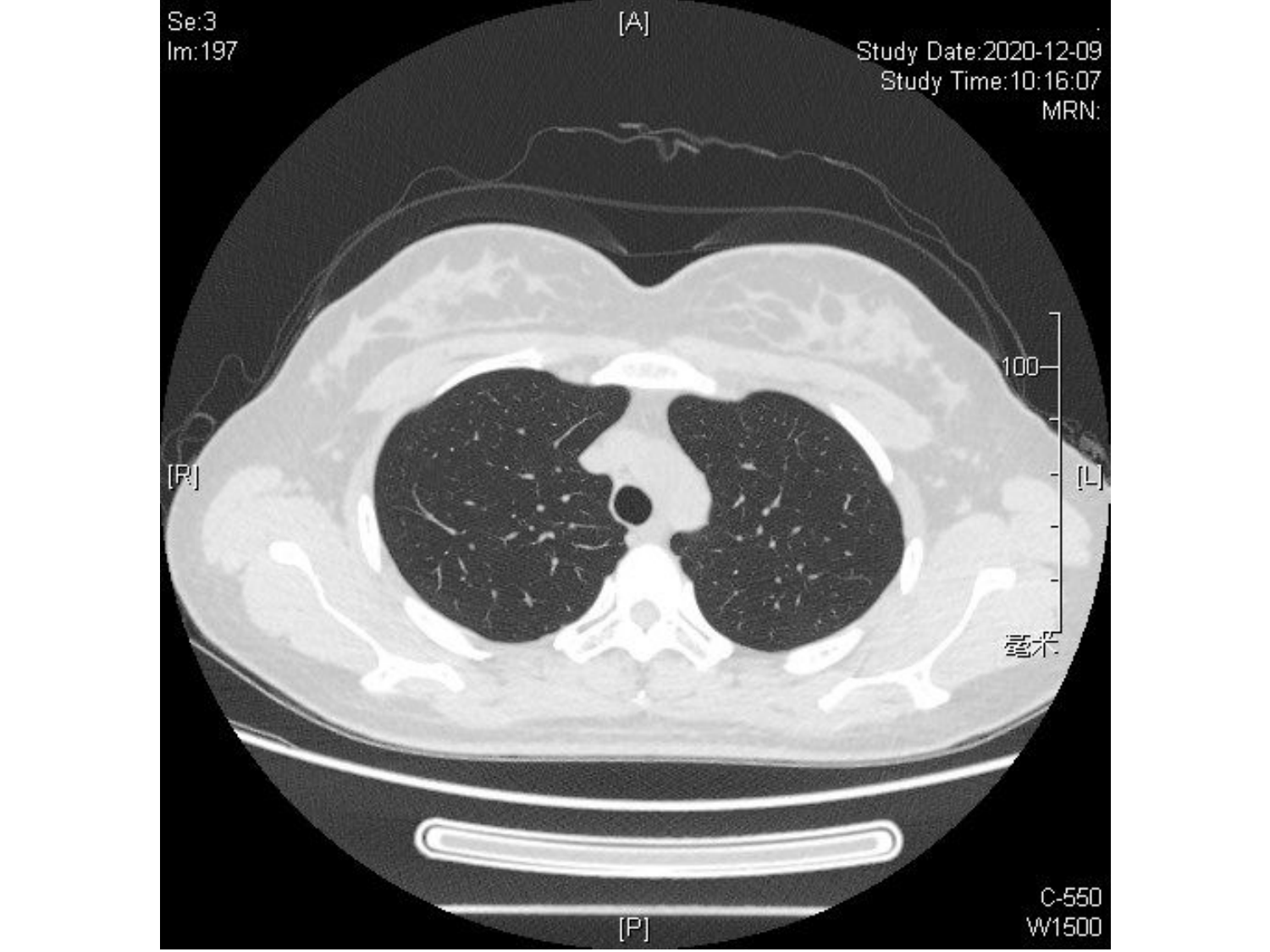

#

## Slide 63
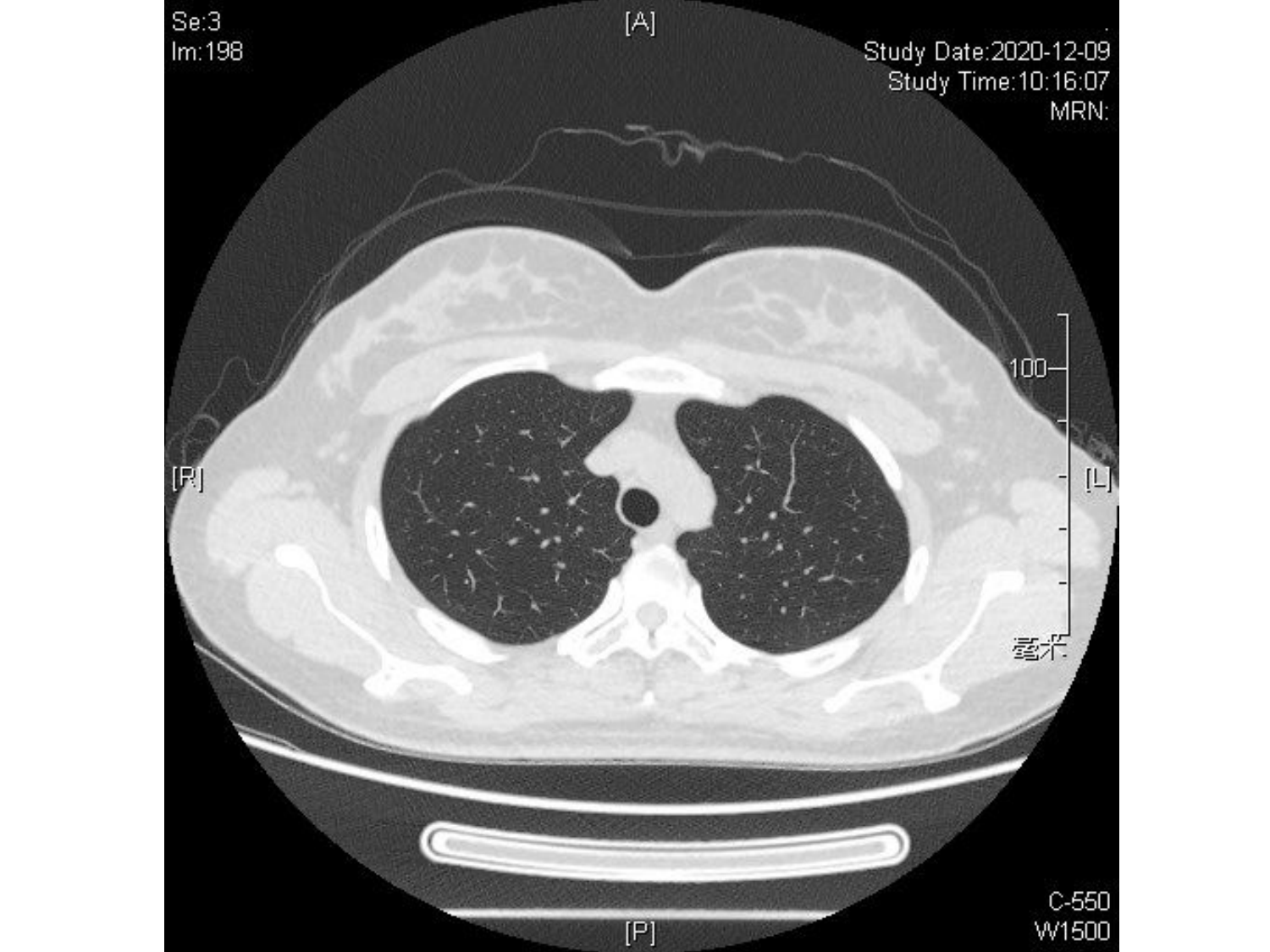

#

## Slide 64
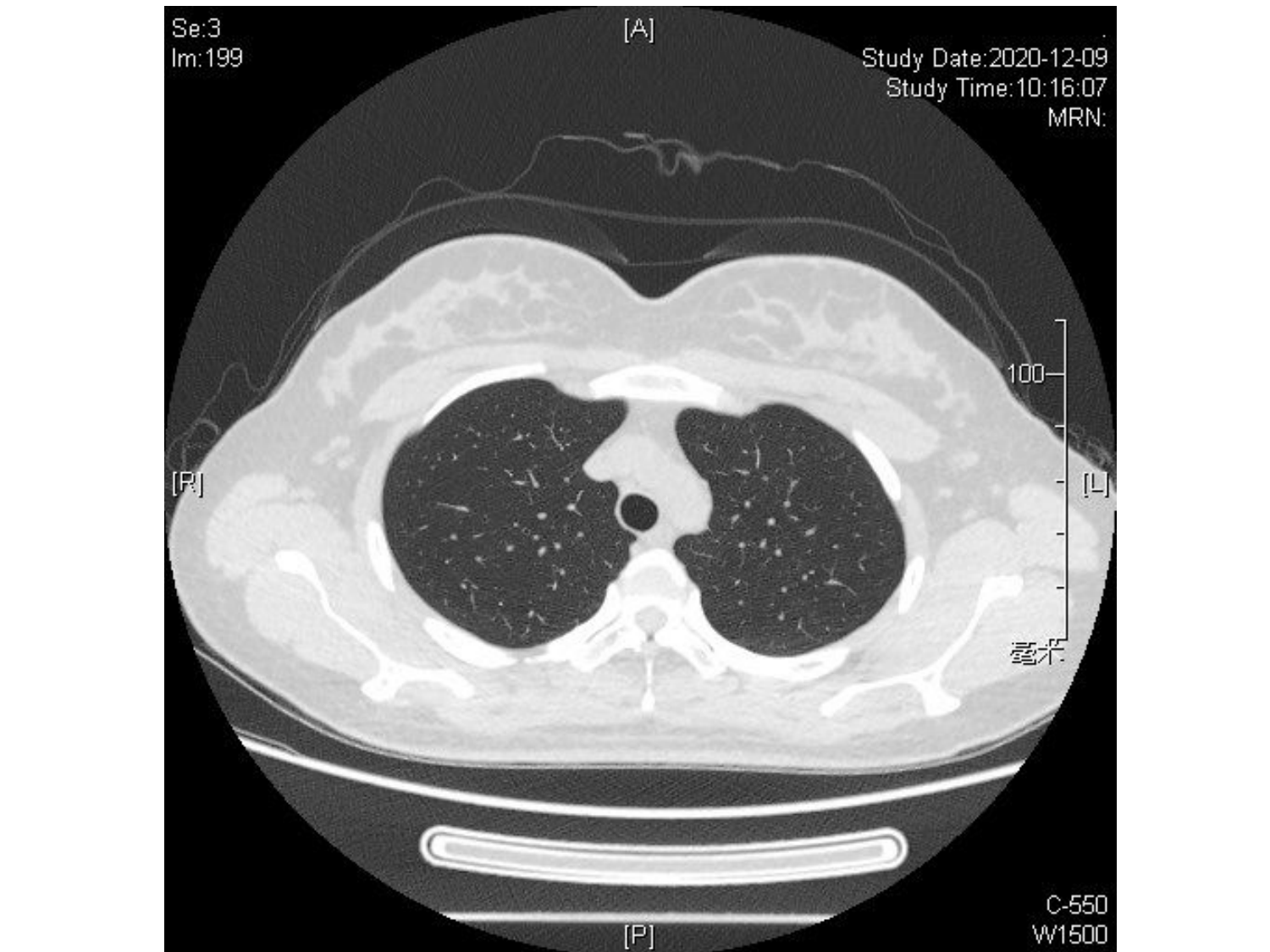

#

## Slide 65
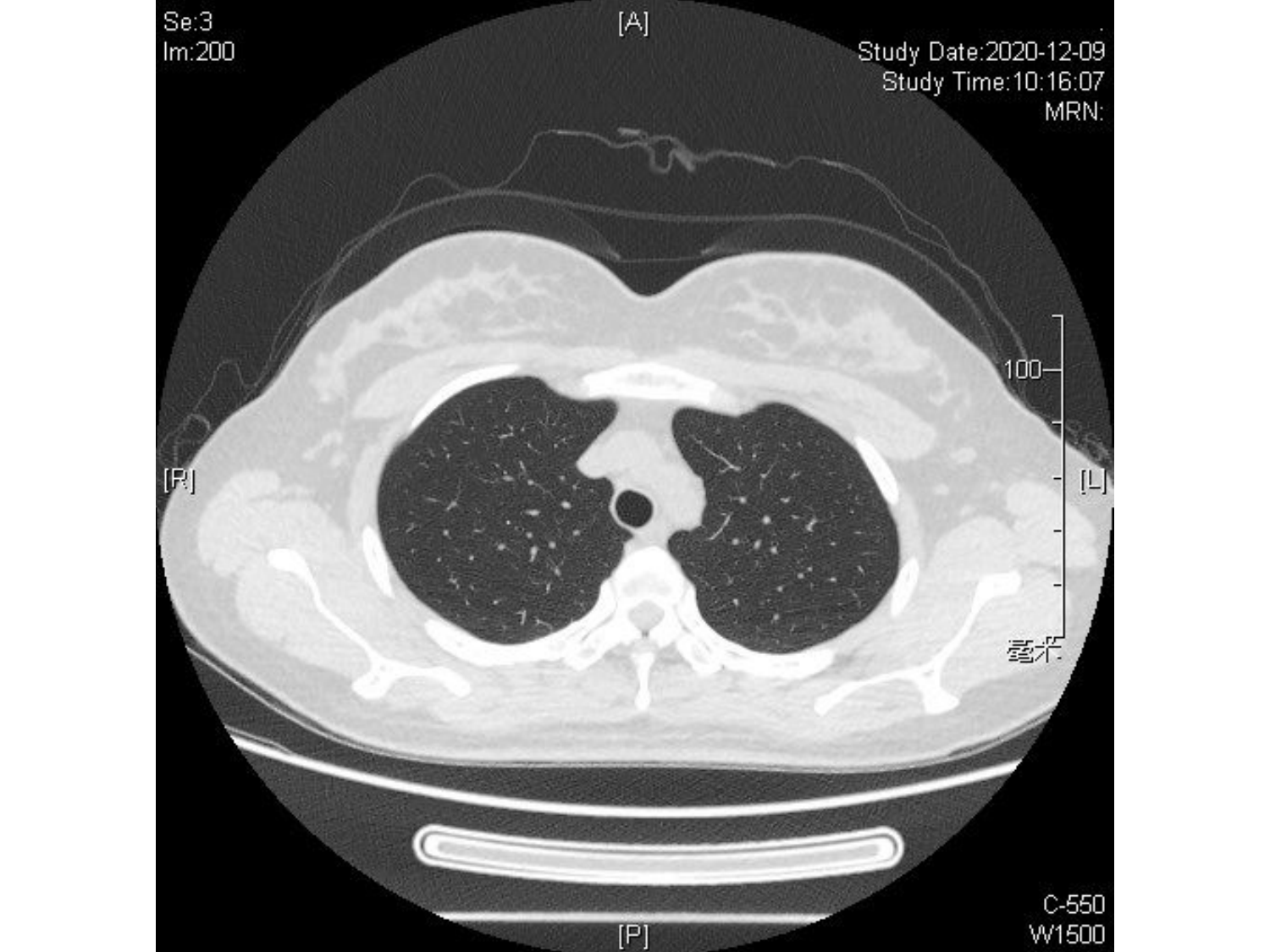

#

## Slide 66
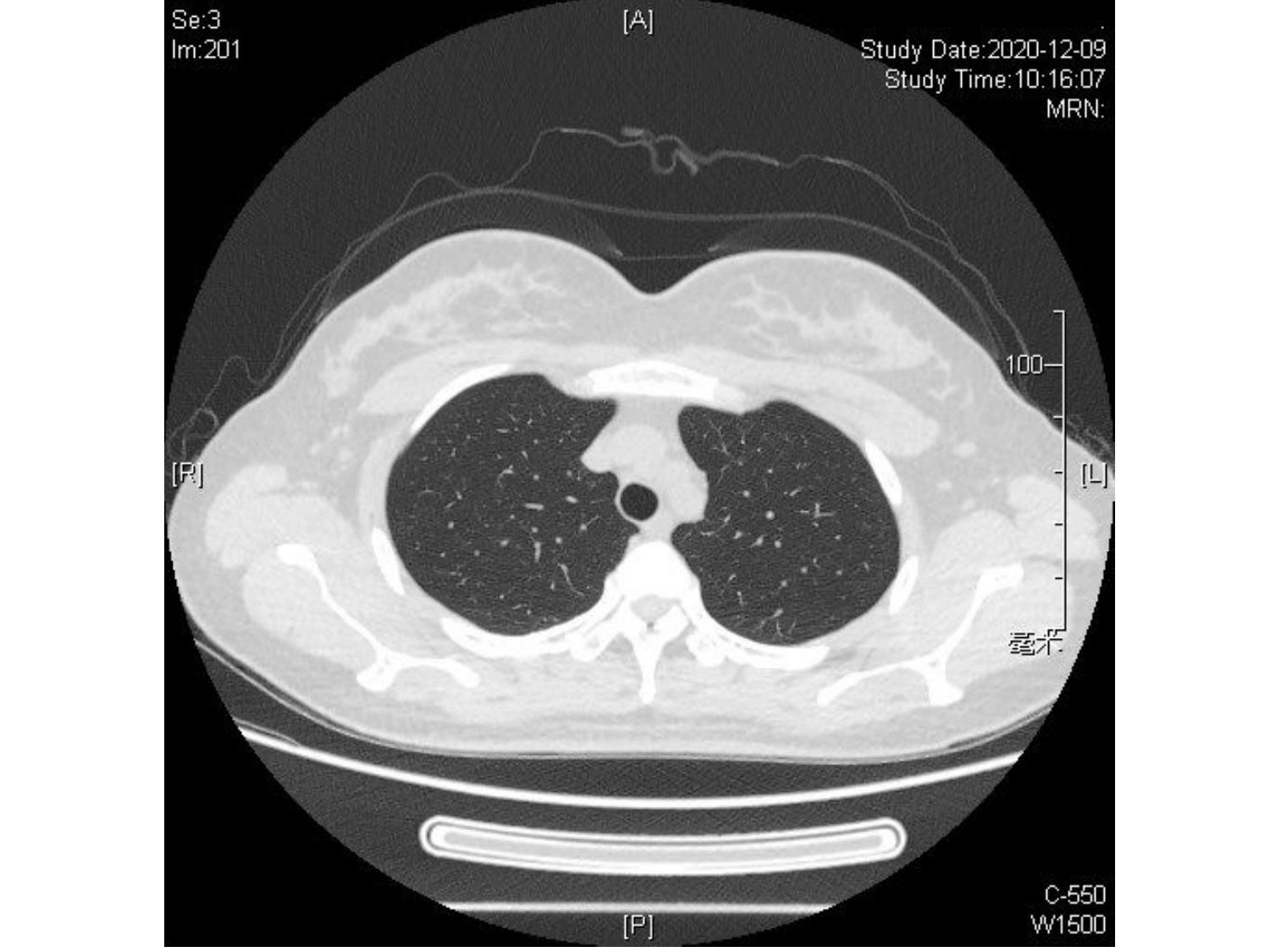

#

## Slide 67
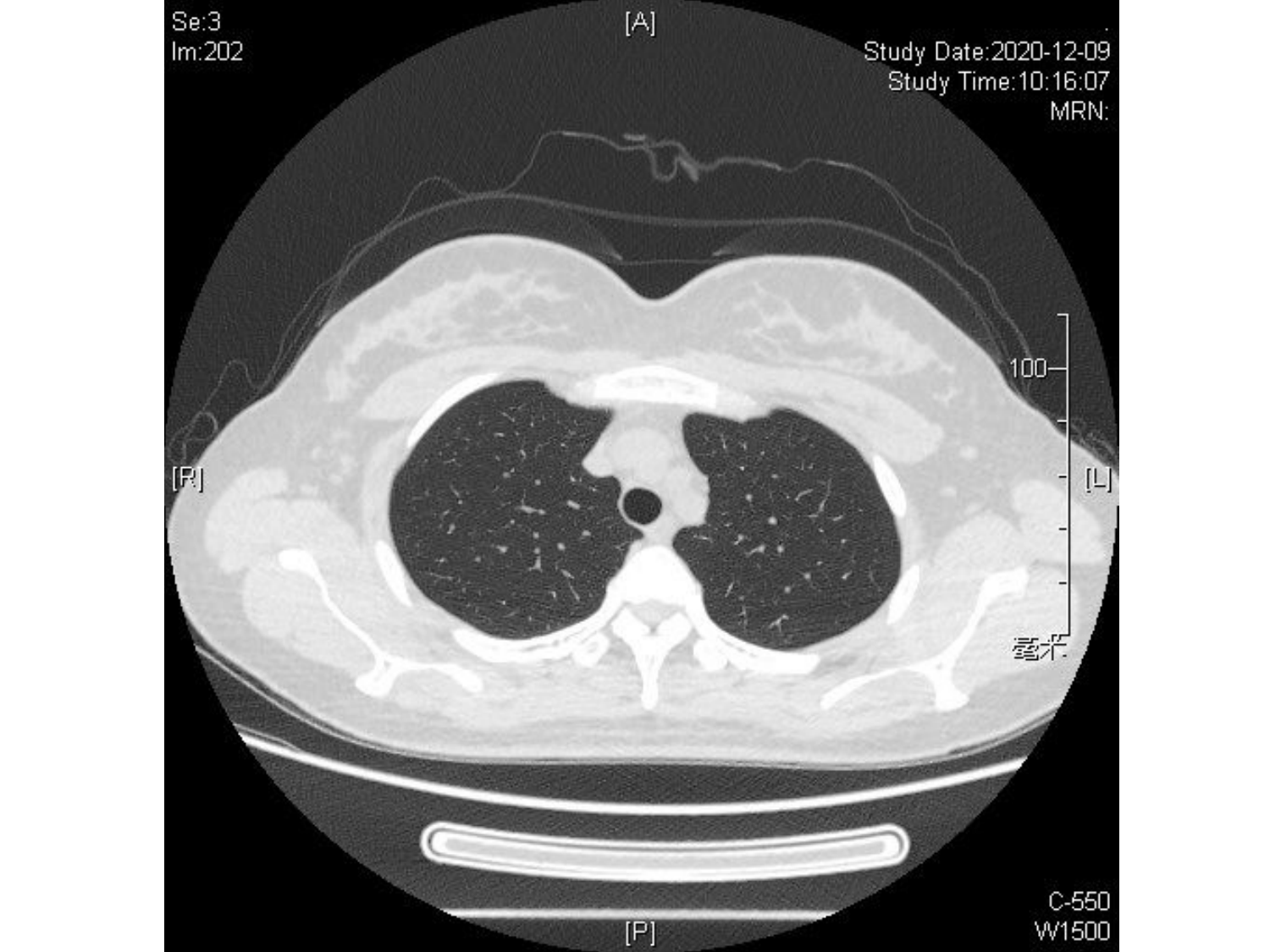

#

## Slide 68
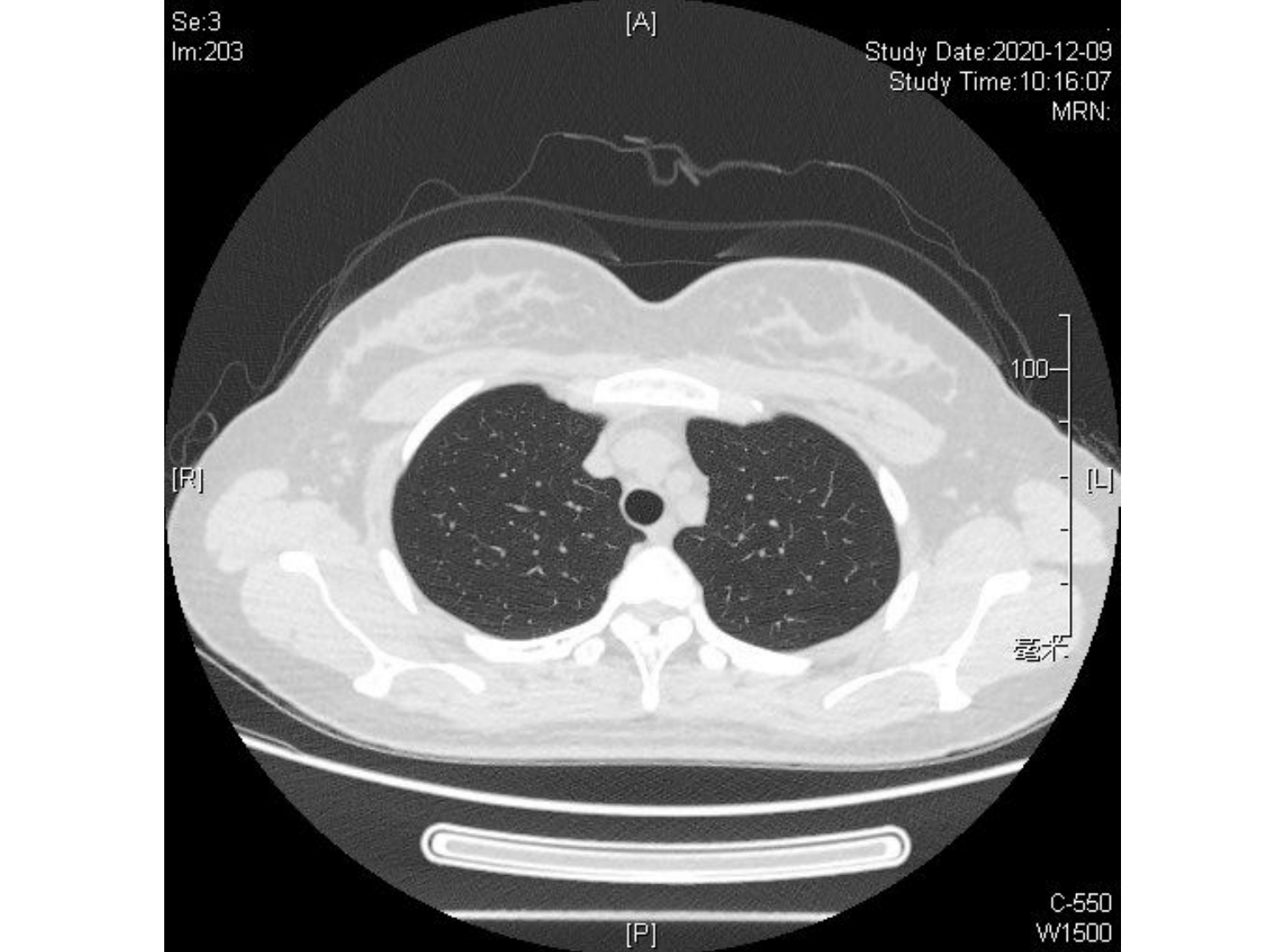

#

## Slide 69
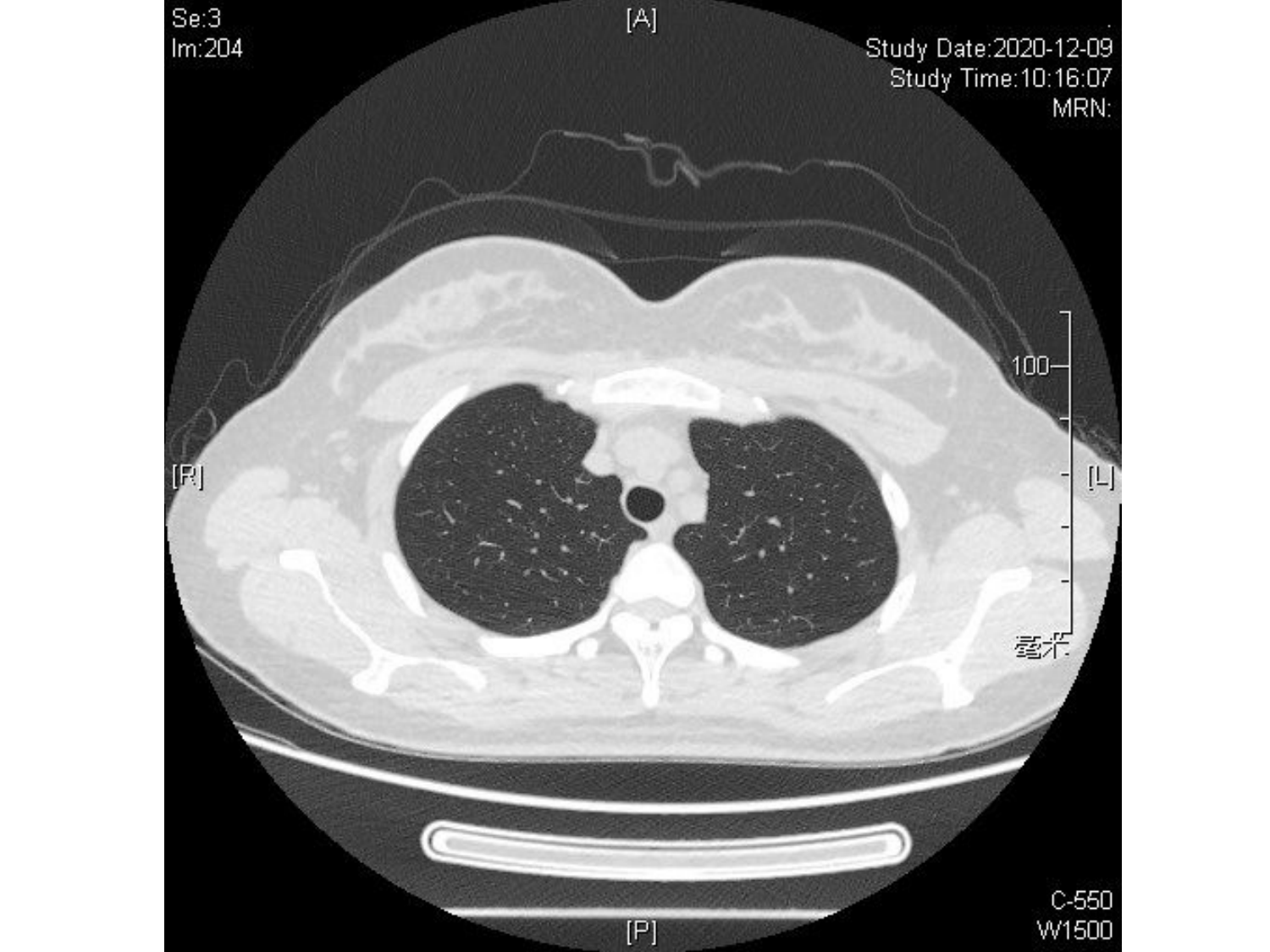

#

## Slide 70
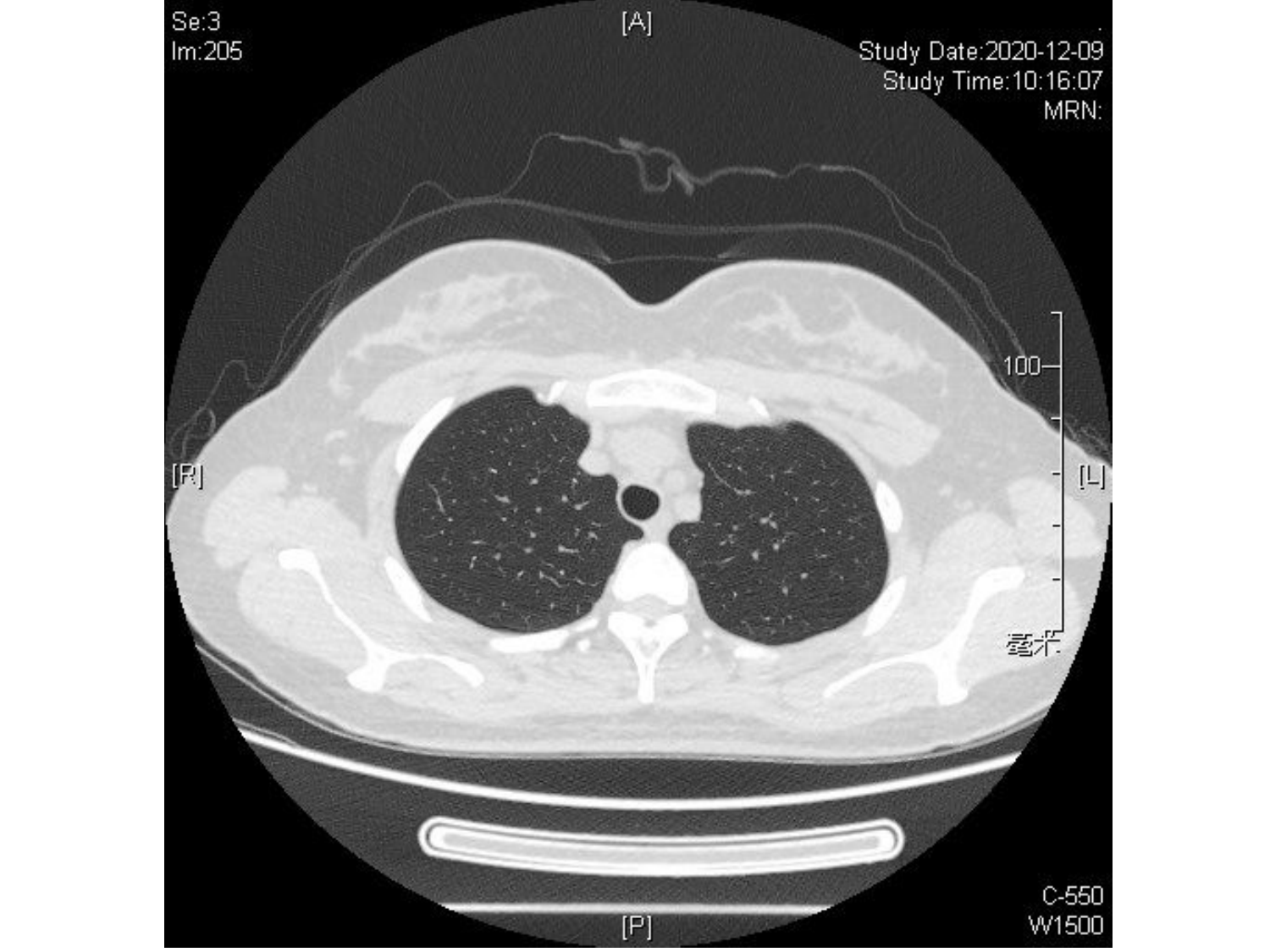

#

## Slide 71
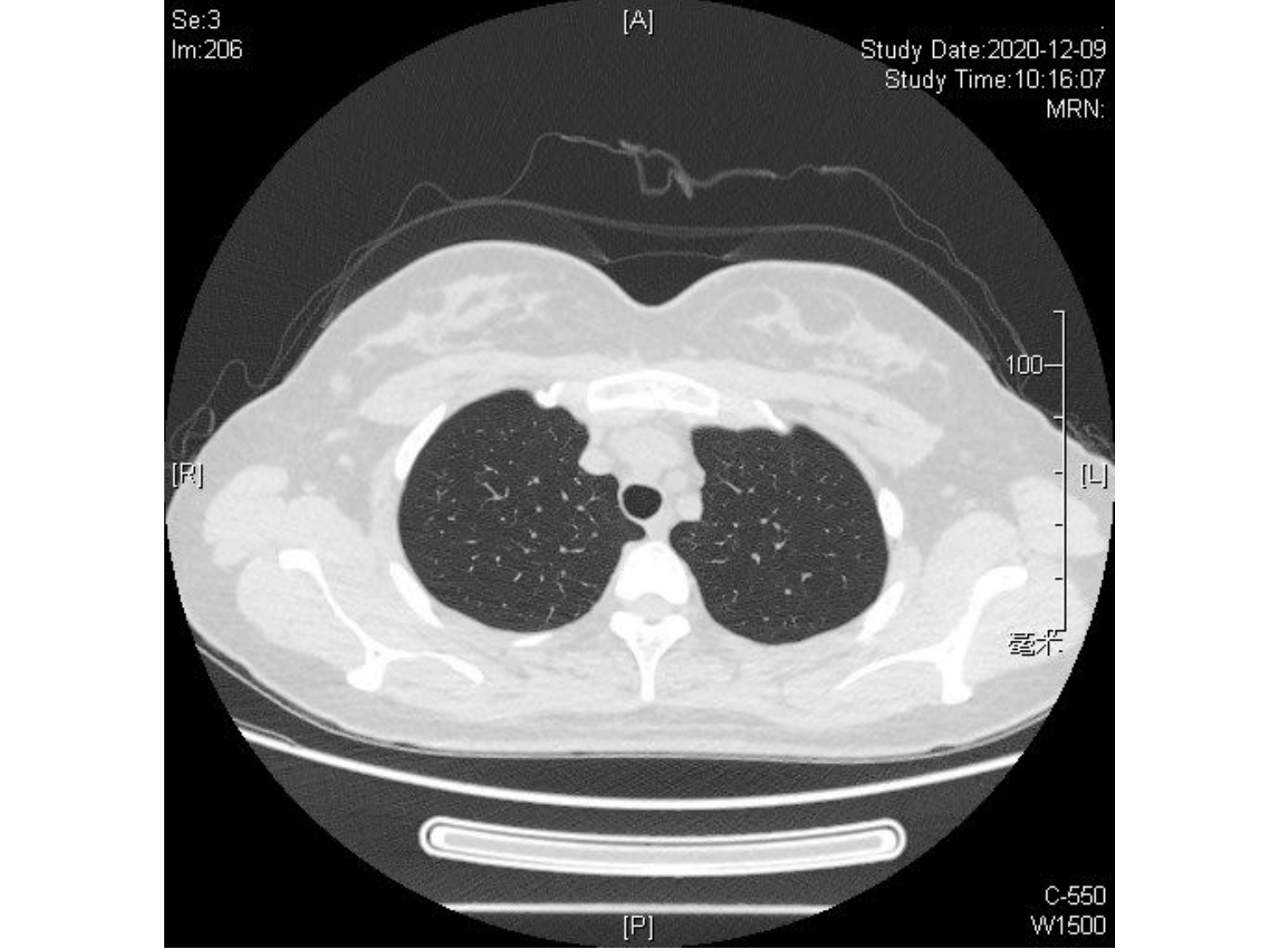

#

## Slide 72
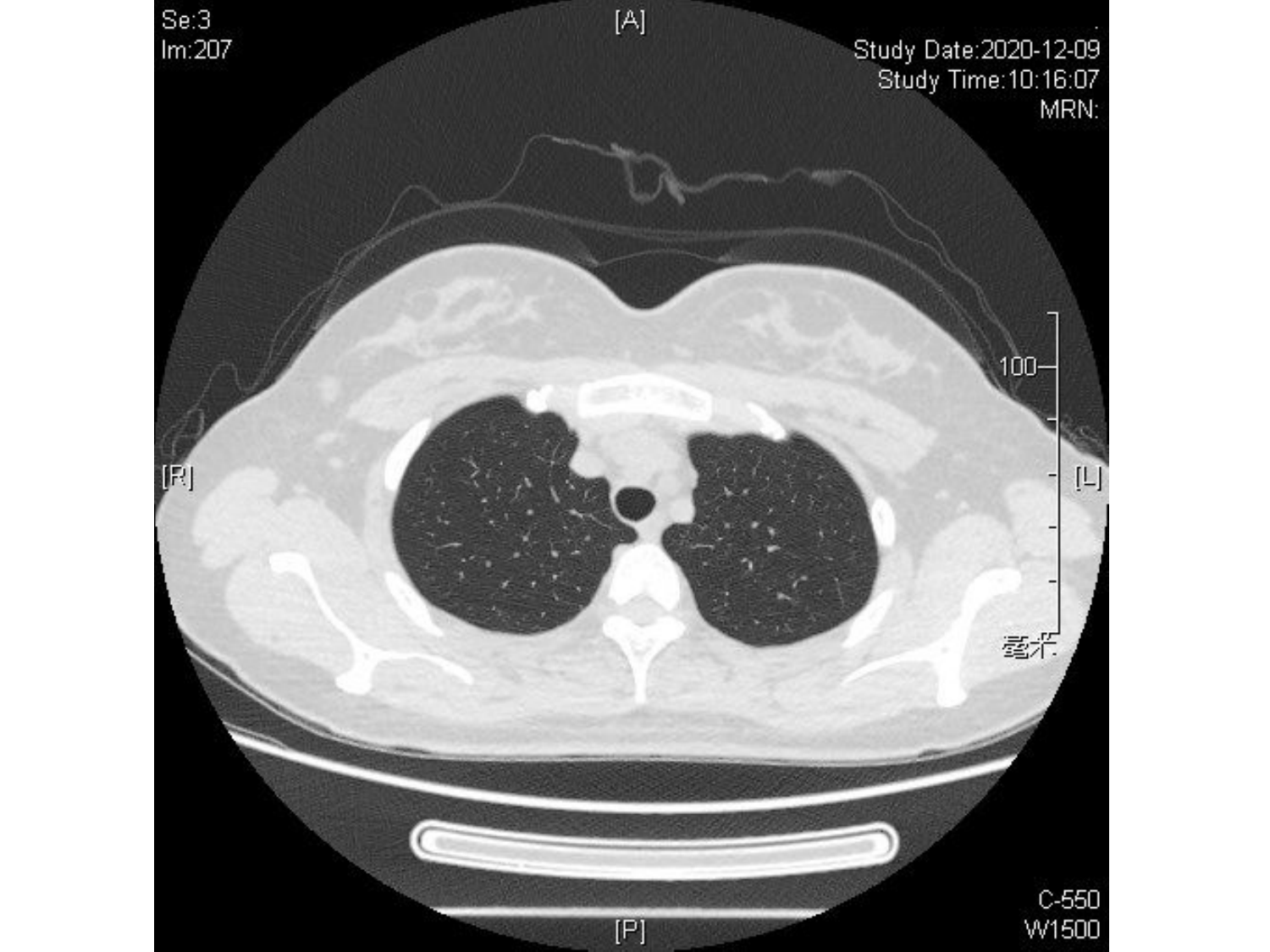

#

## Slide 73
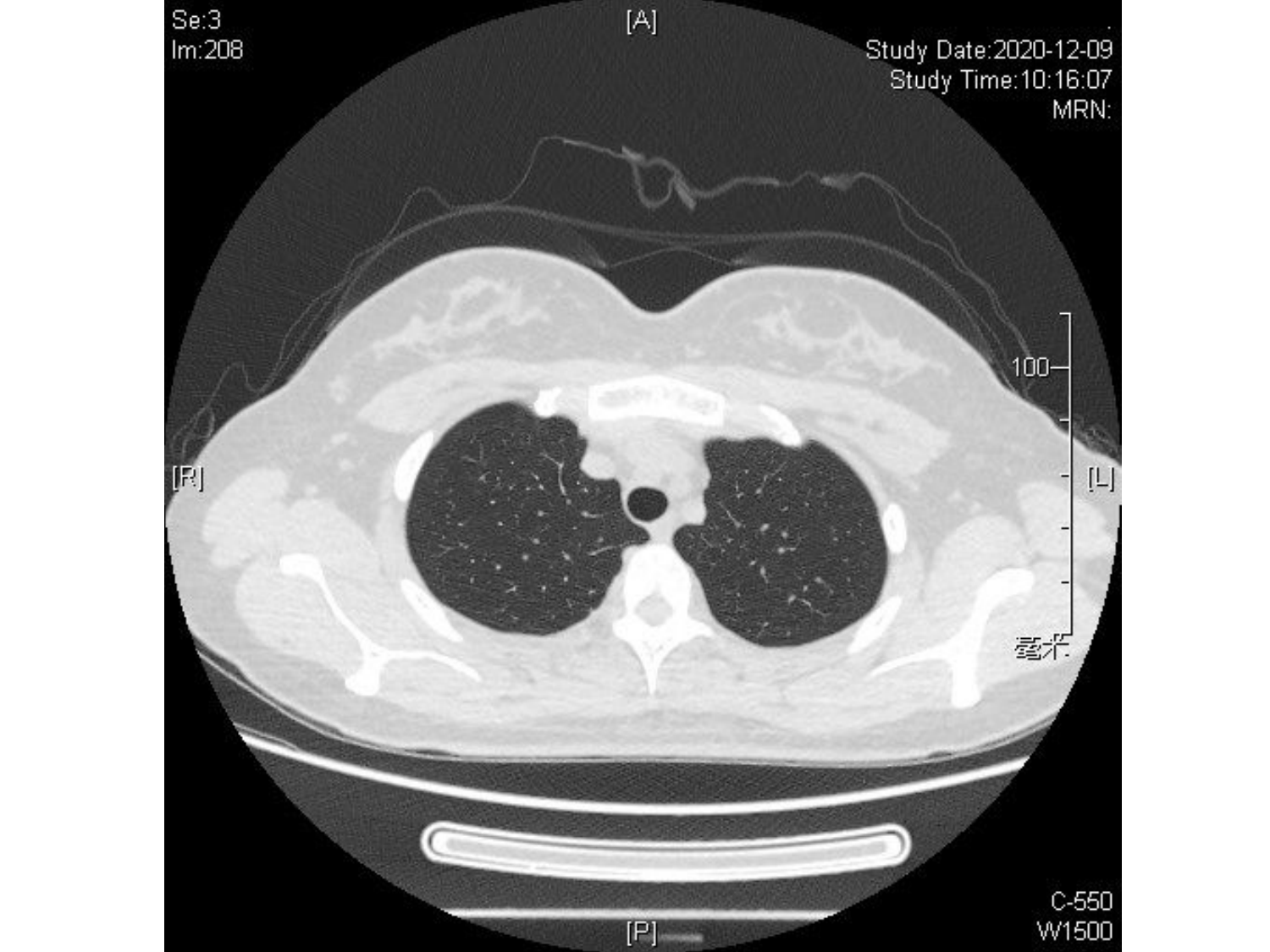

#

## Slide 74
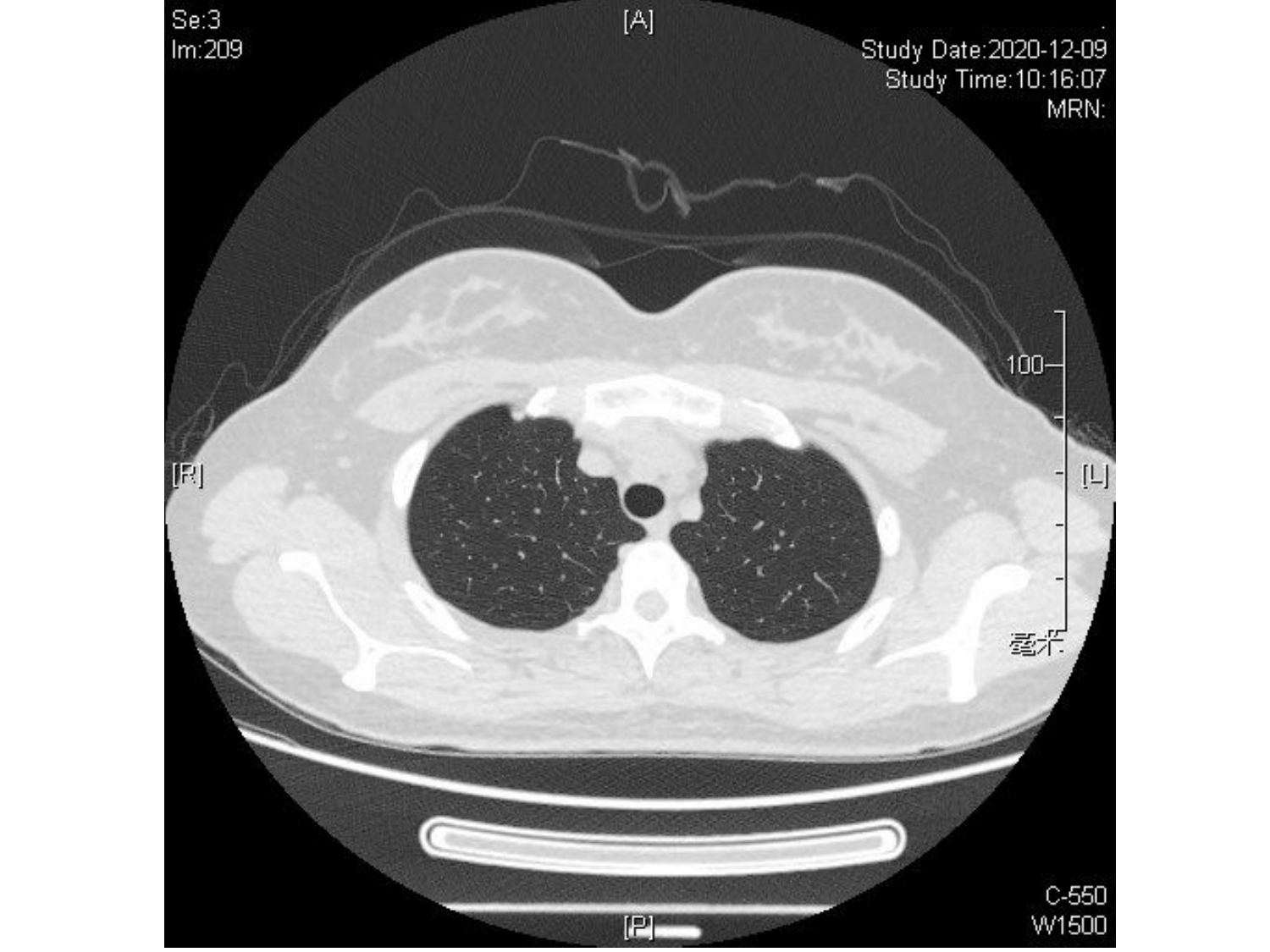

#

## Slide 75
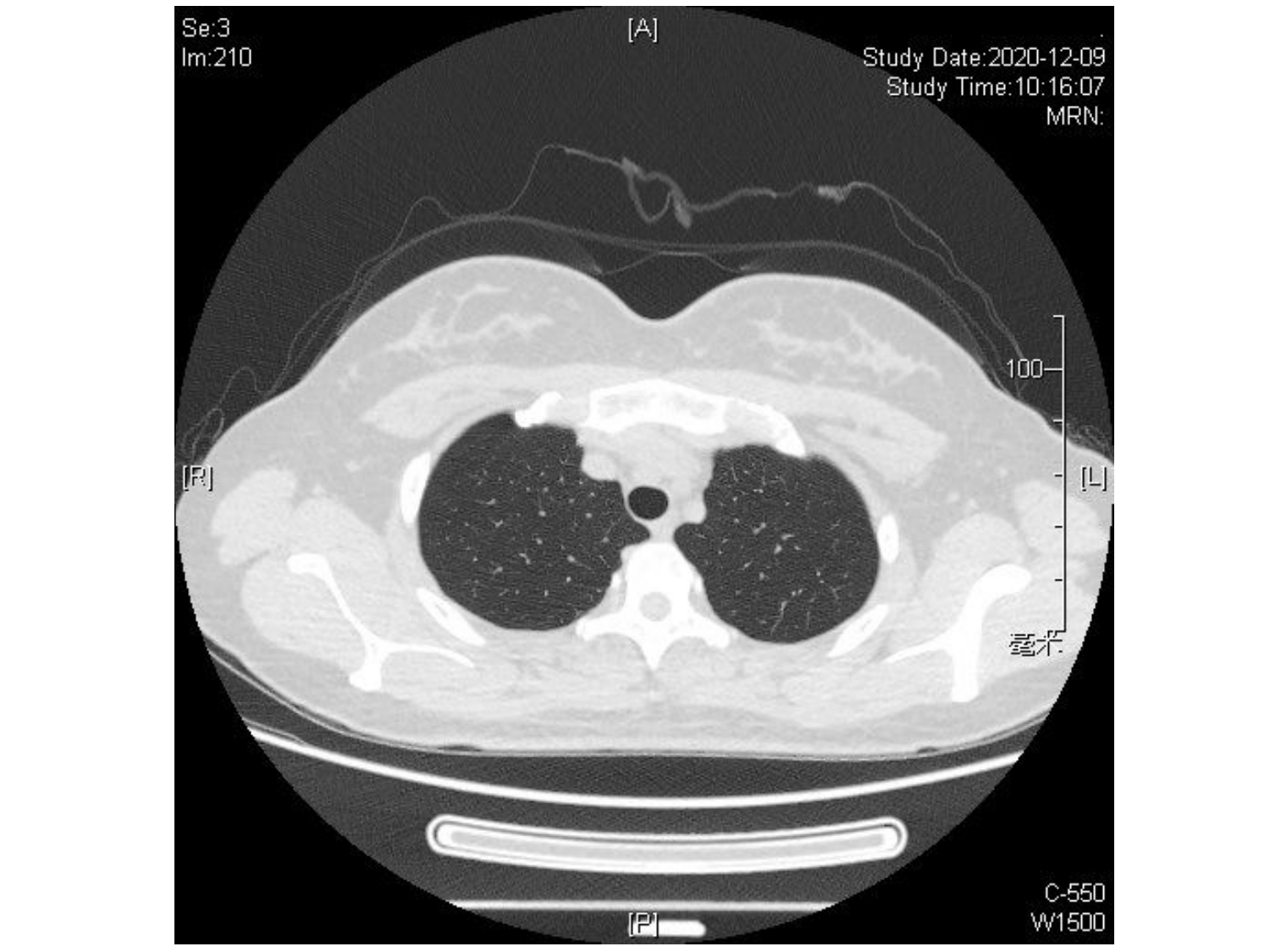

#

## Slide 76
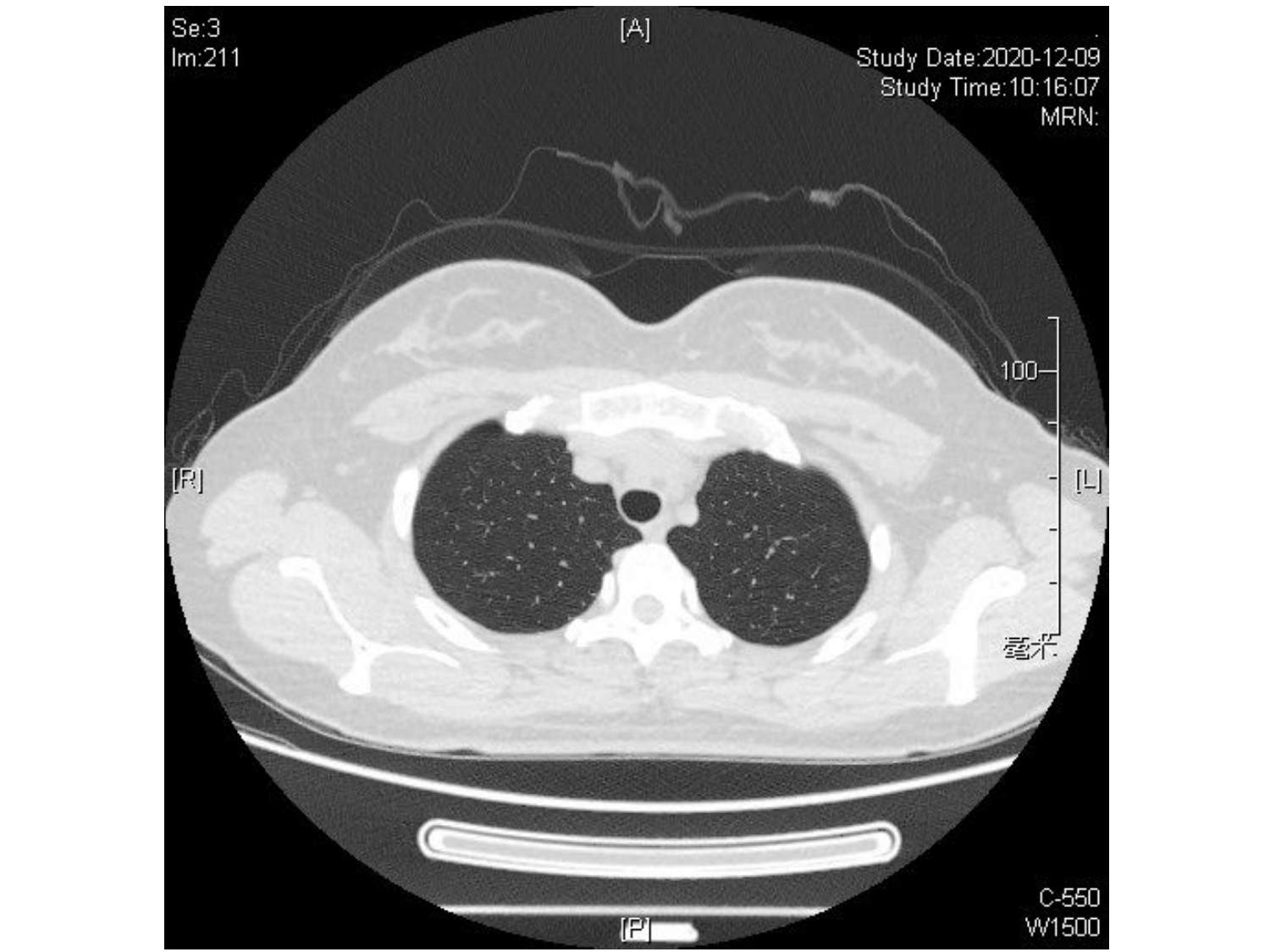

#

## Slide 77
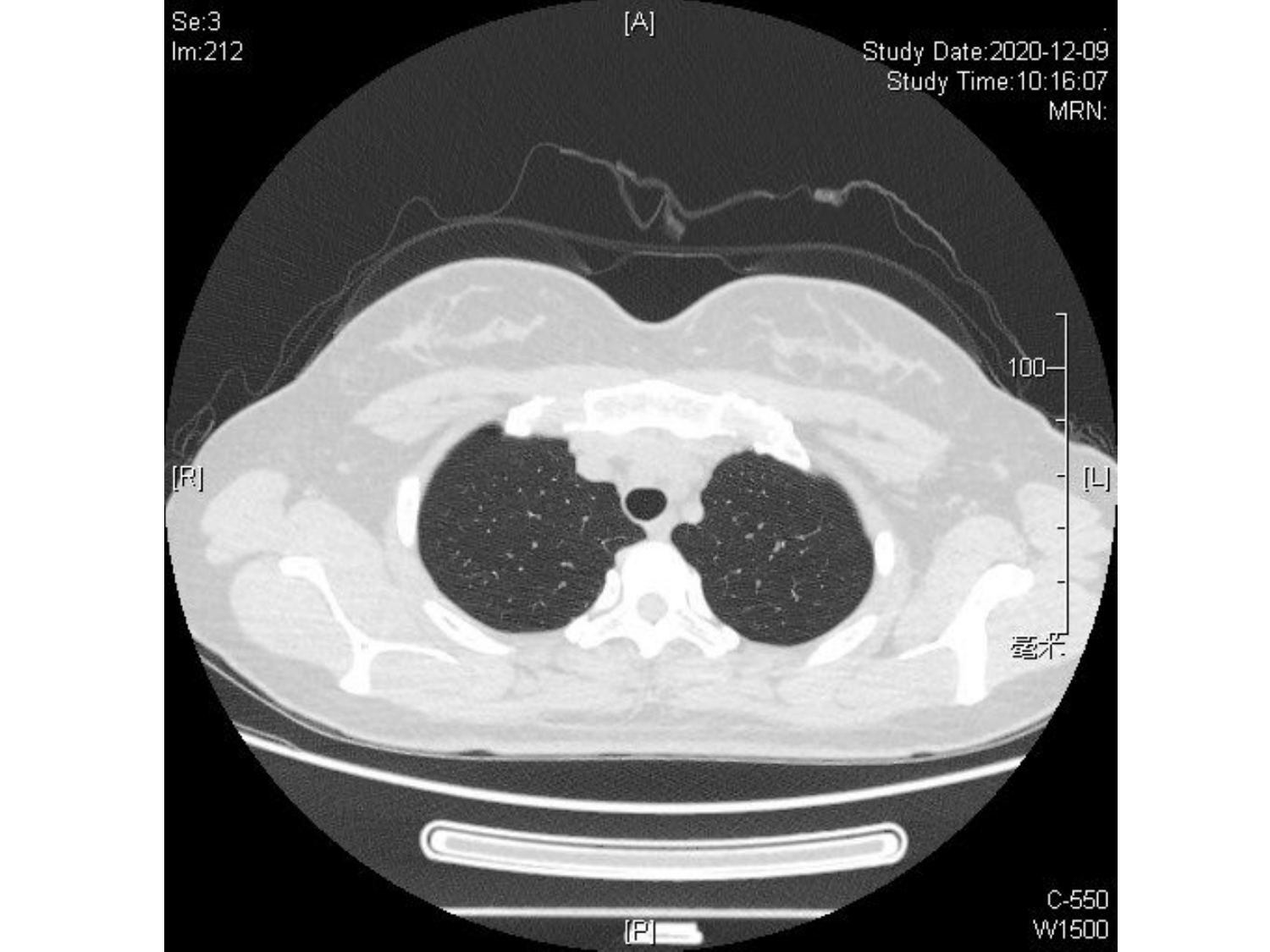

#

## Slide 78
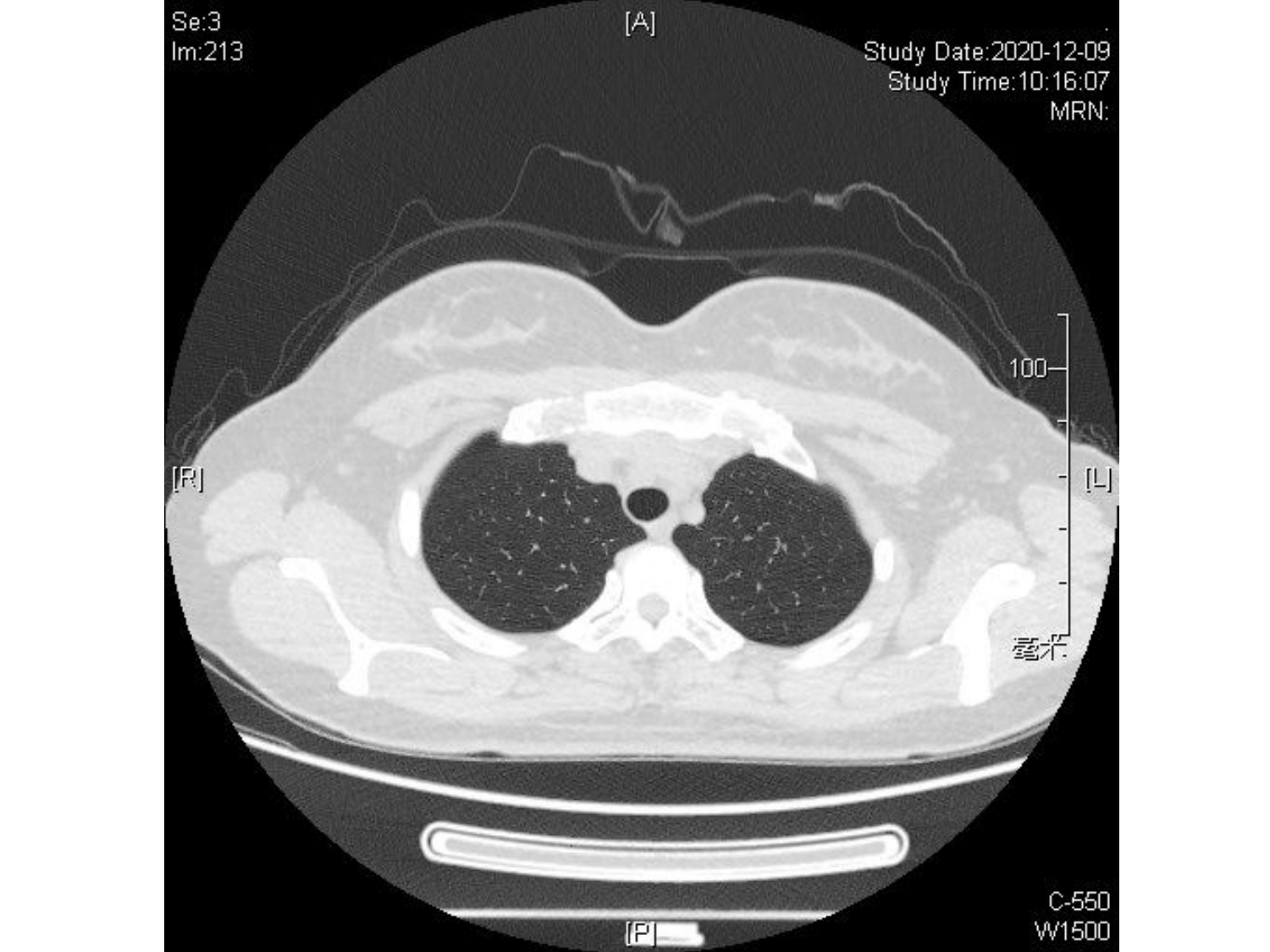

#

## Slide 79
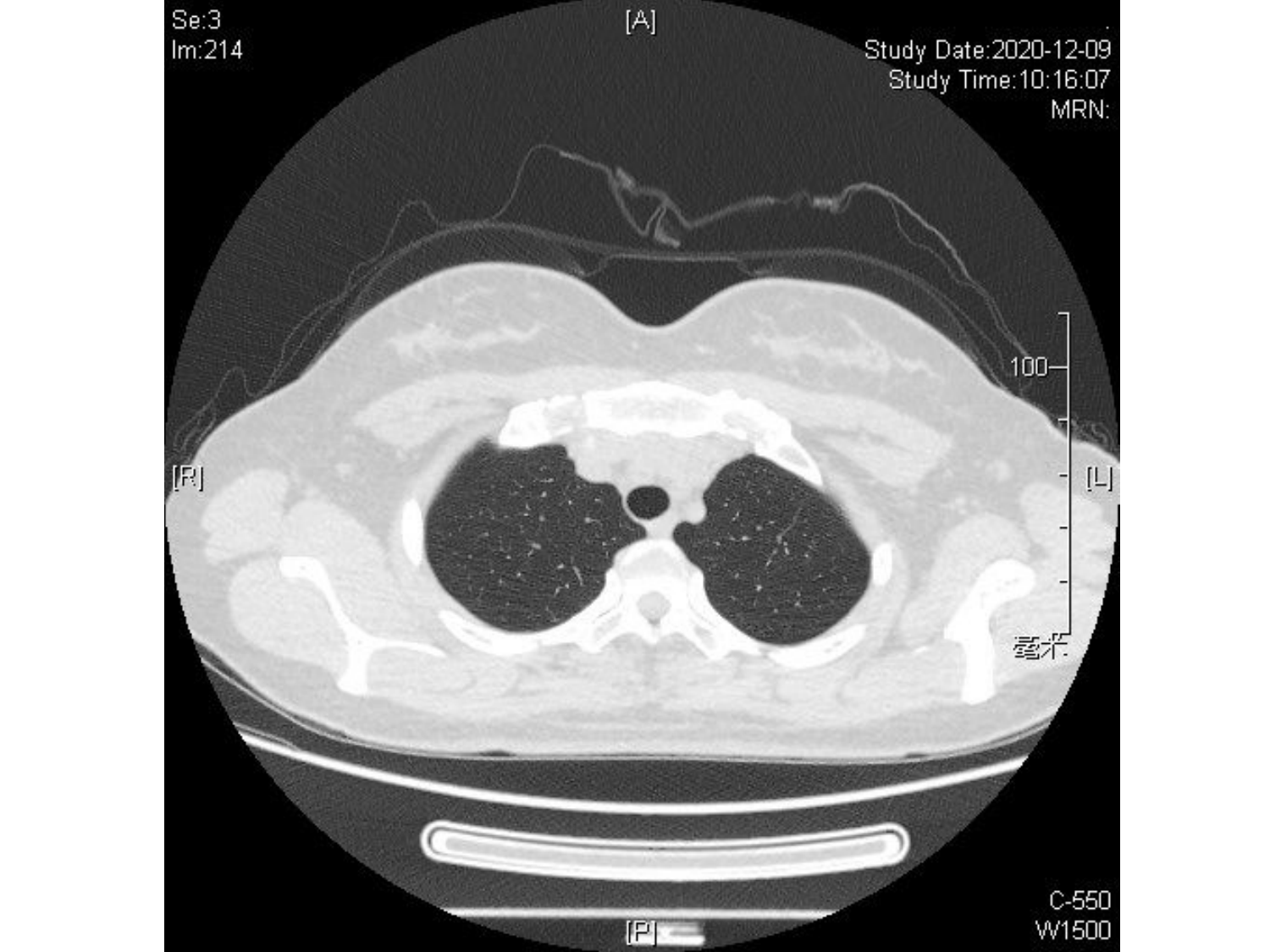

#

## Slide 80
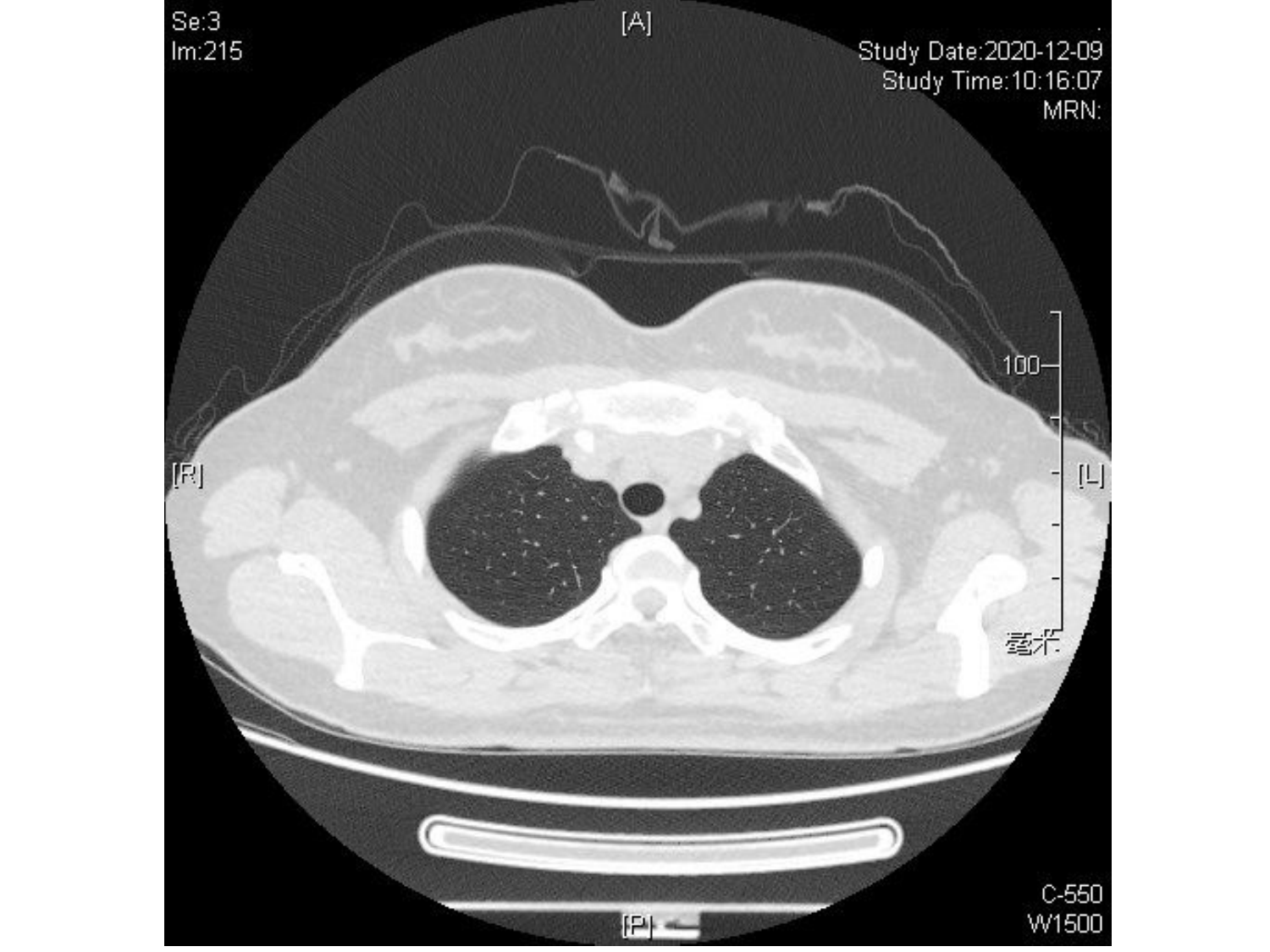

#

## Slide 81
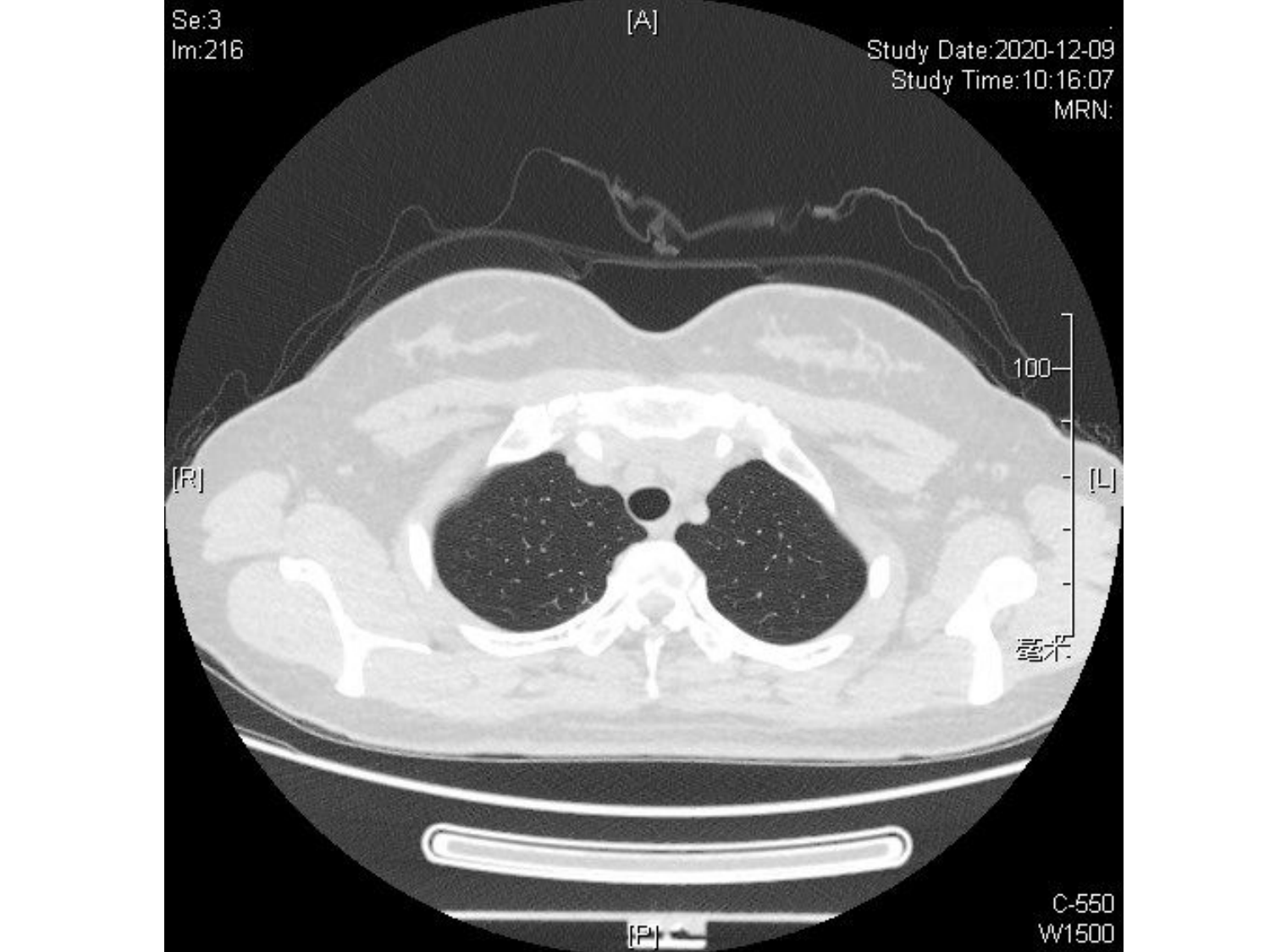

#
